# Dynamics of thyroid diseases and thyroid‐axis gland masses

**DOI:** 10.15252/msb.202210919

**Published:** 2022-08-08

**Authors:** Yael Korem Kohanim, Tomer Milo, Moriya Raz, Omer Karin, Alon Bar, Avi Mayo, Netta Mendelson Cohen, Yoel Toledano, Uri Alon

**Affiliations:** ^1^ Department of Molecular Cell Biology Weizmann Institute of Science Rehovot Israel; ^2^ Department of Computer Science and Applied Mathematics Weizmann Institute of Science Rehovot Israel; ^3^ Division of Maternal Fetal Medicine Helen Schneider Women's Hospital, Rabin Medical Center Petah Tikva Israel

**Keywords:** HPT, hysteresis, pituitary, systems biology, thyroid disorders, Computational Biology

## Abstract

Thyroid disorders are common and often require lifelong hormone replacement. Treating thyroid disorders involves a fascinating and troublesome delay, in which it takes many weeks for serum thyroid‐stimulating hormone (TSH) concentration to normalize after thyroid hormones return to normal. This delay challenges attempts to stabilize thyroid hormones in millions of patients. Despite its importance, the physiological mechanism for the delay is unclear. Here, we present data on hormone delays from Israeli medical records spanning 46 million life‐years and develop a mathematical model for dynamic compensation in the thyroid axis, which explains the delays. The delays are due to a feedback mechanism in which peripheral thyroid hormones and TSH control the growth of the thyroid and pituitary glands; enlarged or atrophied glands take many weeks to recover upon treatment due to the slow turnover of the tissues. The model explains why thyroid disorders such as Hashimoto's thyroiditis and Graves' disease have both subclinical and clinical states and explains the complex inverse relation between TSH and thyroid hormones. The present model may guide approaches to dynamically adjust the treatment of thyroid disorders.

## Graphical abstract

A new mathematical model for the thyroid axis shows that slow changes in thyrotroph mass are responsible for hormone delays in the treatment of thyroid disorders.

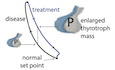

## Introduction

Thyroid disorders affect about 5% of the population and can cause severe symptoms (Vanderpump, 2011). Treating these disorders is complicated in a sizable fraction of cases because the hormone dynamics have fluctuations and delays of many weeks (Dietrich, 2015). These delays and fluctuations are not fully understood. Having a quantitative framework for understanding thyroid‐axis dynamics may help to develop improved treatment protocols and advance our basic understanding of hormone circuits.

Thyroid hormones (TH) T4 and T3 regulate metabolism, affecting almost every cell in the body (Chatzitomaris *et al*, 2017). The thyroid gland secretes T4 and a smaller amount of its active form T3. These hormones enter the circulation, and T4 is converted to T3 by tissue deiodinases. The concentration of TH is controlled by the hypothalamic–pituitary–thyroid axis (the HPT axis) (Dietrich *et al*, 2012). The thyroid gland secretes thyroid hormones when stimulated by thyroid‐stimulating hormone (TSH), which is secreted by the pituitary thyrotroph cells. TSH is secreted in response to thyrotropin‐releasing hormone (TRH) from the hypothalamus. Thyroid hormones inhibit the production and secretion of the upstream hormones TSH and TRH, thus forming a negative feedback loop circuit.

The thyroid axis maintains a TH and TSH set point, which differs slightly between individuals. The variation in the set point for a given individual over time is on the order of 50% of the variation between individuals (Andersen *et al*, 2002). Even small variations of T4 away from its set point trigger large deviations in TSH levels, making TSH a potent diagnostic tool to detect subclinical and clinical states of thyroid dysregulation. High TSH is a sign for hypothyroidism and low TSH for hyperthyroidism.

**Figure 1:**
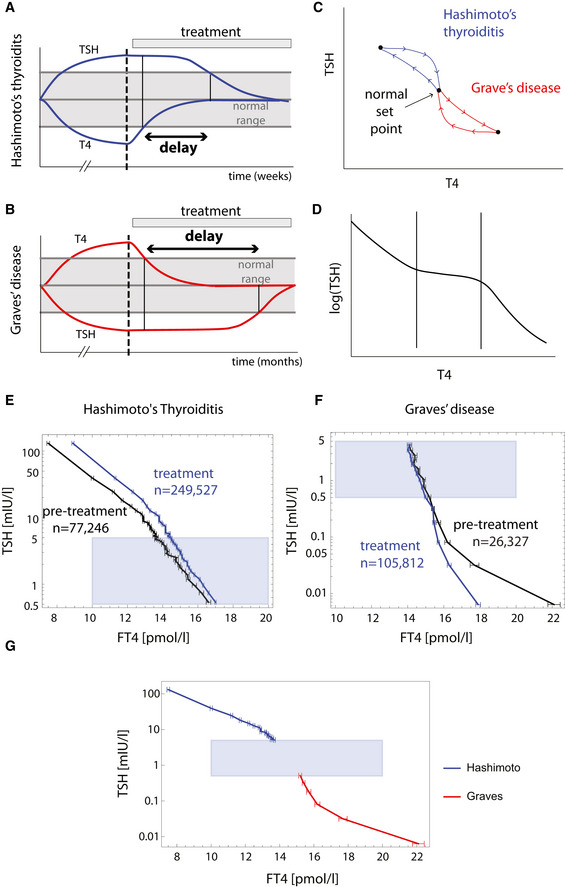
Hysteresis and three‐regime TSH‐T4 relation from a large‐scale medical record dataset A, B
Hysteresis is a delay of serum TSH level equilibration after thyroxine level is normalized. It occurs when thyroid disorders are treated, as schematically seen in Hashimoto's thyroiditis (A) and Graves' disease (B). Note that the disease develops on a much longer timescale than the treatment part of the Figure.C
Hysteresis can be visualized as a loop in the TSH‐T4 plane.D
Schematic of three‐regime TSH‐T4 relation.E
Mean FT4 in bins of TSH in a population before and after treatment of Hashimoto's thyroiditis from the Clalit dataset.F
Mean FT4 in bins of TSH in a population before and after treatment of Graves' disease from Clalit dataset. Gray regions in (E) and (F) are TSH and FT4 in their normal range. Error bars are standard errors.G
Pretreatment means and standard errors for Hashimoto (blue) and Graves (red) patients (same data as in E, F), together with the normal TSH, FT4 range for reference (gray rectangle), forming the inverse “log‐linear” TSH‐FT4 relation.

Treating either hyper‐ or hypothyroidism exposes a fascinating and troublesome delay phenomenon (Braverman & Cooper, 2013), also called hysteresis (Leow, 2007, 2016): it takes many weeks for serum TSH to return to normal after serum TH have been normalized (Fig 1A–C). In hypothyroidism, due to Hashimoto's thyroiditis or thyroidectomy, treatment usually involves supplementing thyroid hormone. It takes TSH about 6 weeks to recover after thyroid hormone normalizes (Fig 1A). In hyperthyroidism, such as in Graves' disease or toxic thyroid nodules, treatment involves the removal of the thyroid or antithyroid drugs that block TH production. Despite comparatively rapid normalization of T4 and T3 concentration, TSH can remain undetectable for months after T4 is normalized (Yu & Farahani, 2015; Leow, 2016) (Fig 1B). These long TSH delays can make it difficult for clinicians to determine drug doses to treat thyroid disorders (Dietrich, 2015).

The TSH delays are too long to be explained by the half‐life of the hormones, which is minutes for TRH, an hour for TSH, and a week for T4 (Mariotti & Beck‐Peccoz, 2000). Hypotheses for the delay in Graves' disease include thyrotroph atrophy, TSH suppression by autoantibodies acting on pituitary or hypothalamic TSH receptors, or TSH negative feedback on hypothalamic TRH secretion (Yu & Farahani, 2015). Temporal changes in treatment dosage were also suggested to play a role (Leow, 2007). However, we still lack a quantitative physiological explanation for the delay that is relevant across the many different conditions in which the delay is observed. Understanding the delay may help guide clinical protocols to optimally balance thyroid hormones.

Here, we provide evidence from large‐scale medical records for hysteresis in the TSH and T4 relation and explain these phenomena by developing a mathematical model of the HPT axis. The model adds to the classical negative feedback circuit (Dietrich *et al*, 2012; Goede *et al*, 2014) a new level of regulation: the effect of the hormones as growth factors for their downstream glands. TSH is a growth factor for the thyroid, and TH inhibits the growth of thyrotroph cells in the pituitary (Pawlikowski *et al*, 1975; Dumont *et al*, 1992). Thus, the circuit includes changes in total cell mass on the timescale of weeks, which affect the hormone secretion capacity of the glands. Such mass changes are well‐known in the case of goiter, an enlarged thyroid that can compensate for low iodine levels (Marine & Kimball, 1917; Braverman & Cooper, 2013). Mass changes also occur in the shrinkage of pituitary thyrotroph mass in hyperthyroidism (Scheithauer *et al*, 1992), and hypertrophy and hyperplasia of pituitary thyrotrophs in primary hypothyroidism (Scheithauer *et al*, 1985; Ahmed *et al*, 1989; Khawaja, 2006; Shukla *et al*, 2019). However, these gland‐mass changes have rarely been considered in a dynamical model of thyroid function (Berberich *et al*, 2018; Pandiyan *et al*, 2018).

The present model naturally gives rise to the delays and hysteresis, due to slow recovery of pituitary thyrotroph mass on the scale of many weeks. The ability of glands to vary in mass helps to compensate for physiological changes, which explains the robustness of each individual's TH set point. The breakdown of this compensation, due to a maximal secretory capacity of the glands, explains the transition between subclinical and clinical disorders.

In addition to hysteresis and delays, the model explains a second issue of clinical importance, namely the precise relation between T4 and its regulator TSH. TSH levels decline steeply with increasing T4 levels, both on the population level and in longitudinal studies (Fig 1D). This inverse relation, which is traditionally referred to as the “log‐linear TSH/T4 relation” (Spencer *et al*, 1990), is used to diagnose states of hypo and hyperthyroidism. Since the first descriptions of the inverse relation in the 1960s (Reichlin & Utiger, 1967), its exact nature has been debated (Leow, 2007, 2010; Hoermann *et al*, 2010; Hadlow *et al*, 2013; Fitzgerald & Bean, 2016; Rothacker *et al*, 2016). Midgley *et al* and others pointed out that the TSH/T4 relation seems to be composed of three distinct regimes corresponding to low, intermediate, and high T4 levels (Midgley *et al*, 2013; Fitzgerald & Bean, 2016). The gland‐mass model explains the complex inverse relation between TSH and T4, with three regimes that stem from distinct clinical states of hyper and hypothyroidism.

## Results

### Large‐scale medical records show hysteresis and a three‐regime TSH–TH relationship

To explore thyroid hormones with a large population sample, we used the Clalit HMO medical record database (Balicer & Afek, 2017). Clalit HMO is Israel's largest health‐care fund, and the dataset includes about half of Israel's population over a period of 18 years, for a total of 46 million person‐years. We obtained anonymized data for TSH and free T4 (FT4) blood tests performed in the morning. Each test is associated with ICD9 disease codes and drugs bought by the individual. These codes include diagnoses of Hashimoto's thyroiditis and Graves' disease.

We investigated hysteresis by analyzing data for individuals diagnosed with overt Hashimoto's thyroiditis, which typically causes decreased FT4 and increased TSH. We compared test results taken before the date of diagnosis (n = 77,246 tests, see Materials and Methods), to the test results after diagnosis and during treatment (n = 249,527 tests). Median FT4 was evaluated in bins of TSH. The relation between TSH and FT4 shows hysteresis, where for a given FT4 level, TSH is higher after diagnosis than before diagnosis (Materials and Methods) (Fig 1E).

We also analyzed data from individuals before and after diagnosis with overt Graves' disease (*n* = 26,327, *n* = 105,812, respectively), which causes increased FT4 and decreased TSH. TSH levels are lower for a given FT4 level after diagnosis with Graves' disease than before diagnosis (Fig 1F), showing hysteresis.

These findings generalize previous case studies of individual trajectories of hysteresis in patients with Graves' disease, Hashimoto’s thyroiditis and post‐thyroidectomy (Leow, 2016).

Clalit data also allowed us to explore the relation between TSH and FT4 values obtained in blood tests taken on the same day (Materials and Methods). We find an inverse relationship between TSH and T4 as previously reported. The relationship can be divided into three regimes. At TSH above its normal range (TSH >5 mIU/L), corresponding to individuals with Hashimoto's before diagnosis and treatment, TSH is a declining function of FT4, with a log‐linear slope of −0.228 ± 0.007. When TSH is below its normal range (TSH <0.5 mIU/L), corresponding to individuals before diagnosis and treatment of Graves' disease, the log‐linear slope is −0.23+/−0.04. Serum TSH level usually drops to undetectable levels when FT4 begins to exceed its upper normal range (FT4 > 20 pmol/L) (Fig 1G).

### Model for gland‐mass dynamics in the thyroid axis

In the next sections, we first present a mathematical model for the HPT axis. Second, we show how the model explains (i) delays and hysteresis, (ii) the ability of the HPT axis to compensate for physiological changes by means of gland‐mass growth and shrinkage, (iii) the transition between subclinical and clinical states of Hashimoto's and Graves' diseases, and (iv) the three‐regime TSH/T4 relationship.

**Figure 2:**
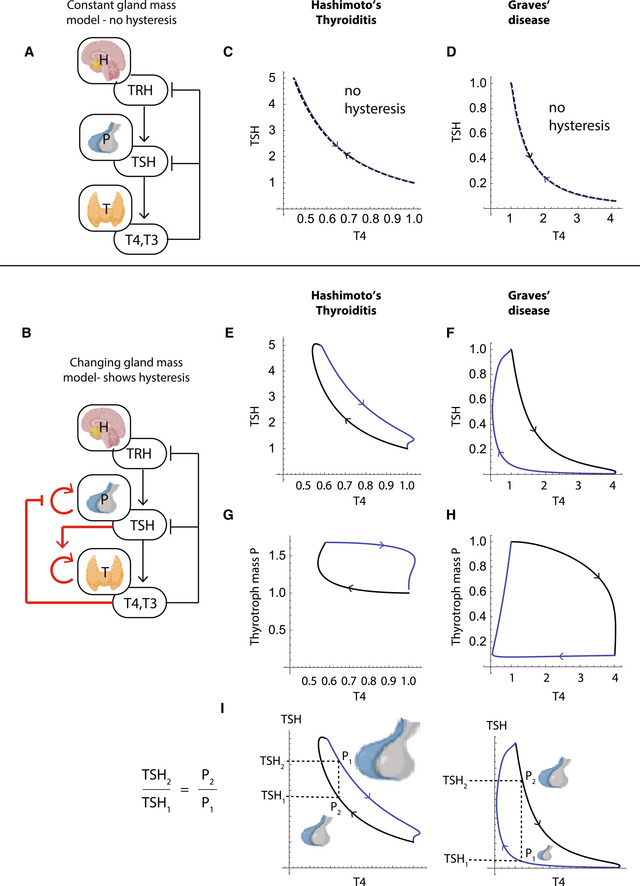
Gland‐mass model explains hysteresis by a slow recovery of thyrotroph mass A
HPT model with constant gland mass.B
HPT model with gland‐mass changes, with new interactions in red. Thyrotroph and thyrocyte turnover times are on the order of a month.C, D
The constant mass model does not show hysteresis in simulations of (C) Hashimoto's thyroiditis and (D) Graves' disease. TSH is uniquely determined by TH levels.E, F
TSH‐T4 trajectories in a model with gland‐mass changes show TSH delay in Hashimoto's thyroiditis (E) and in Graves' disease (F).G, H
TSH‐thyrotroph mass *P* trajectories show that the delay is due to an enlarged thyrotroph mass that takes many weeks to shrink back to baseline after T4 is normalized in Hashimoto's thyroiditis (G), or due to atrophied thyrotroph mass that takes months to regrow after T4 is normalized by treatment in Graves' disease (H).I
Model indicates that the ratio of thyrotroph masses at a given T4 level before and after treatment is equal to the ratio of TSH levels. Data information: For all panels, Black lines represent trajectories during disease, from the normal set point to hypo/hyperthyroidism. Blue lines represent trajectories after diagnosis and during treatment back to the normal set point.

To explore the mechanism for these phenomena, we developed a mathematical model of the HPT axis. The innovation in the model is the consideration of the slow timescale regulation of the functional mass of the thyroid and pituitary thyrotroph cells (Fig 2A and B). This regulation is due to the effect of TSH as a growth factor for the thyroid and of TH as inhibitor of thyrotroph cell growth. Similar gland‐mass models were developed for the beta‐cell‐insulin system (Topp *et al*, 2000; De Gaetano *et al*, 2008; Ha *et al*, 2016; Karin *et al*, 2016). Recent gland mass models also helped to explain slow timescales in cortisol dynamics (Karin *et al*, 2020, 2021; Maimon *et al*, 2020), hormone seasonality (Tendler *et al*, 2021) and to offer a theory of endocrine autoimmune disease (Korem Kohanim *et al*, 2020).

We begin with the main result and provide the model details below. We find that a model without gland‐mass changes cannot provide hysteresis (Fig 2C and D), because there is a unique value for TSH determined by the T4 level, both before and after treatment. In contrast, the present model, whose equations are provided below, shows delays and hysteresis (Fig 2E and F). The reason is changes in the thyrotroph functional mass, *P* (Fig 2G and H). During hypothyroidism, reduced T4 level releases the inhibition of *P* growth. Thyrotroph mass grows, as observed in clinical samples from patients with Hashimoto's thyroiditis (Scheithauer *et al*, 1985), and in radiological scans of hypothyroid patients (Khawaja, 2006; Shukla *et al*, 2019). When treatment starts, it takes weeks for *P* to decrease back to baseline due to the slow thyrotroph mass turnover (Fig 2G).

Similarly, during hyperthyroidism (Fig 2H), high T4 levels inhibit thyrotroph growth. Thyrotroph mass *P* atrophies, as seen in clinical samples of Graves' patients and of patients with toxic multinodular goiter (Scheithauer *et al*, 1992). Upon treatment, it takes *P* months to recover, leading to a delay in TSH normalization.

We now present the model equations and in the next sections use them to understand thyroid disorders and the T4/TSH relationship.

The model builds on seminal thyroid‐axis modeling work that described the fast timescale hormonal regulation (Leow, 2007; Dietrich *et al*, 2012, 2016; Goede *et al*, 2014; Fitzgerald & Bean, 2016; Goede & Leow, 2018) (Fig 2A). We add equations for the dynamics of total functional mass of the pituitary thyrotroph cells and thyroid thyrocyte cells (Fig 2B, red arrows).

The production and removal of TRH, TSH, and TH are described by three equations. Thyroid hormone TH is secreted by the thyroid gland, whose functional mass is *T*, when stimulated by TSH:

(1) $$\mathrm{dTH}/\mathrm{dt}=b_{\mathrm{TH}}T\;f\left(\mathrm{TSH}\right)-a_{\mathrm{TH}}\mathrm{TH}$$

where $a_{\mathrm{TH}}$ is the removal rate of TH, $b_{\mathrm{TH}}$ is the maximal TH secretion rate per unit thyroid mass, and $f\left(\mathrm{TSH}\right)$ is the TSH‐regulation function (Dietrich *et al*, 2012).

TSH is secreted by pituitary thyrotrophs, whose total mass is *P*, and this secretion is stimulated by TRH and inhibited by TH:

(2) $$\text{dTSH}/\mathrm{dt}=b_{\mathrm{TSH}}\text{P}\;\text{TRH}/\mathrm{TH}-a_{\mathrm{TSH}}\mathrm{TSH}$$

here, we use a 1/TH term for the inhibition, which approximates a Michalis–Menten‐like term $1/\left(1+\mathrm{TH}/K\right)$ when TH≫K.

TRH secretion is inhibited by TH and stimulated by a hypothalamic input *u* which represents the integrated effect of cues including temperature, illness, and nutritional states:

(3) $$\text{dTRH}/\mathrm{dt}=b_{\mathrm{TRH}}u/\mathrm{TH}\;-\;a_{\mathrm{TRH}}\mathrm{TRH}$$

The hormone removal rate parameters are the inverse of the hormone lifetimes (with the usual factor of log2 to convert turnover rates to half‐lives), to give half‐lives of 7d for TH, 1 h for TSH, and 6 min for TRH (Mariotti & Beck‐Peccoz, 2000; Dietrich *et al*, 2016).

Note the important assumption that the secretion rate of a hormone is proportional to the total gland mass: doubling thyroid mass, *T*, as occurs for example in goiter, is assumed to double the secretion rate of TH at a given level of TSH and secretion parameter $b_{\mathrm{TH}}$. Similarly, secretion of TSH is assumed to be proportional to the total mass of the pituitary thyrotroph cells *P*, at a given level of TRH and TH.

We added two equations for the effective thyrocyte mass *T* and pituitary thyrotroph mass *P*, which we call for brevity thyroid and pituitary functional masses. The equation for the thyroid functional mass has a removal/turnover term −$a_{T}T$ and a mass growth term activated by *TSH*, $Tb_{T}f\left(\mathrm{TSH}\right)$. We find below that in order to understand thyroid diseases, it is essential to also impose a limit to thyroid mass growth. This limit is known as a *carrying capacity*. We use a standard term for carrying capacity from ecology, which was experimentally tested in fibroblasts (Zhou *et al*, 2018), in which the growth rate goes to zero when *T* reaches carrying capacity $1/k_{T}$ as $\left(1\;-\;k_{T}\;T\right)$. Thus, the rate of change of thyroid mass is:

(4) $$\mathrm{dT}/\mathrm{dt}=T\left(b_{T}f\left(\mathrm{TSH}\right)\left(1\;-\;k_{T}\;T\right)\;-\;a_{T}\right)$$

The growth rate term here is meant to include both hypertrophy and hyperplasia.

The pituitary thyrotroph mass, *P*, follows a similar balance of growth and removal, where we assume that the main control of mass growth is a suppressive effect by TH (Kunert‐Radek & Pawlikowski, 1975):

(5) $$\mathrm{dP}/\mathrm{dt}=P\left(b_{P}\left(1\;-\;k_{P}\;P\right)/\mathrm{TH}\;-\;a_{P}\right)$$

where $a_{P}$ is the removal rate of thyrotrophs, and the carrying capacity (maximal mass) of thyrotrophs is $k_{P}$.

The growth of thyrotroph cells can occur by additional means (Nolan *et al*, 2004) such as trans‐differentiation from other pituitary cells (Horvath *et al*, 1990; Radian *et al*, 2003), progenitor cells, or de‐differentiated subpopulations (Wang *et al*, 2014) (Appendix Fig S1, See analysis of these cases in Appendix Supplementary Text).

The timescales of the mass Equations (4) and (5) are much slower than those of the hormone Equations (1–3): the turnover times of the hormones are minutes (TRH), hours (TSH), or days (TH), whereas the turnover time of cell functional mass is a month or more (Dumont *et al*, 1992; Mariotti & Beck‐Peccoz, 2000; Dietrich *et al*, 2016). In the present simulations, we use turnover times of one month for both tissues ($a_{T}=a_{P}=1/30\;\text{days}$). Slower turnover times show qualitatively similar effects.

### Dynamic compensation of physiological changes

**Figure EV1:**
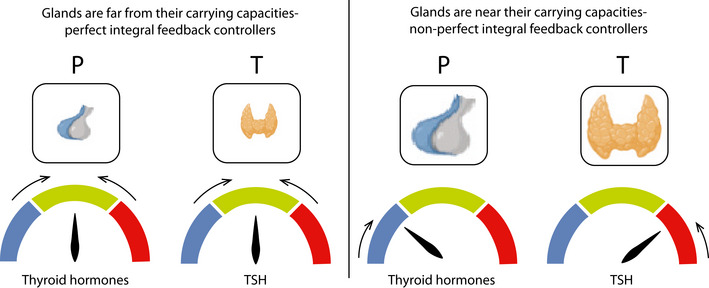
Thyrotroph mass *P* and thyrocyte mass *T* as integral feedback controllers for thyroid hormones and TSH, respectively Left: When the glands are far from their carrying capacities, they provide perfect integral feedback control, keeping the hormones at their homeostatic set points. Right: When the glands approach their carrying capacities, they serve as imperfect integral controllers and can only partially prevent the hormones deviation from their set points.

When the glands are far from their carrying capacity ($P\ll 1/k_{P},T\ll 1/k_{T}$), the gland masses can grow or shrink to provide compensation for physiological changes. For example, low TH levels lead to two complementary compensation mechanisms. The classic fast mechanism is that more TSH is secreted from the pituitary, increasing TH secretion from the thyroid gland to restore homeostasis. This rise of TSH in the model is due primarily to the release of inhibition of thyroid hormones on TSH production in the pituitary. A secondary effect, which is not essential for our conclusions, is due to the release of TRH inhibition. We note that the role of TRH in this context is debated in the literature (Samuels *et al*, 1993; Rabeler *et al*, 2004; Hoermann *et al*, 2015b). If this is not sufficient, and TH remains low on a longer timescale, the mass *P* of thyrotrophs increases, due to reduced inhibition by TH in equation (5). The enlarged thyrotroph mass enhances TSH secretion, gradually causing increased thyroid mass (equation 4), with both effects increasing TH back to baseline. The adaptation of TSH and TH results from the integral feedback nature of equations (4) and (5). When the glands are far from their carrying capacities, the only possibility to get a steady state with nonzero gland sizes is for hormone‐controlled proliferation to equal removal, and hence $\mathrm{TSH}=\frac{a_{T}}{b_{T}},\mathrm{TH}=\frac{b_{P}}{a_{P}}$ (Fig EV1).

**Figure 3:**
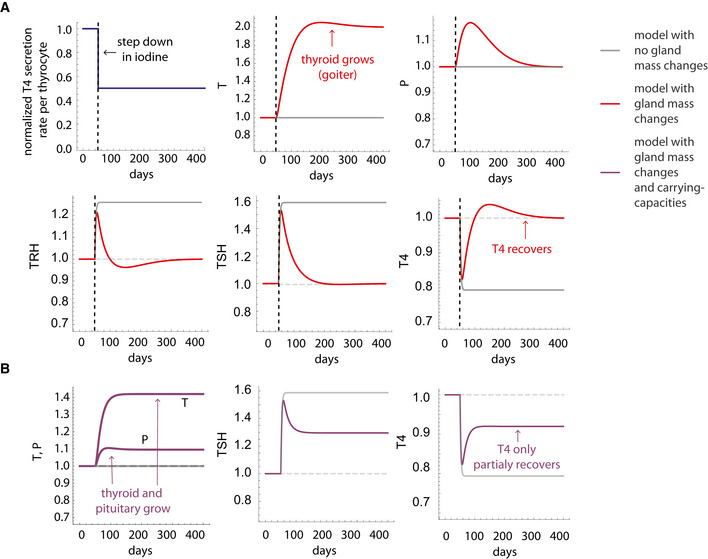
Gland‐mass model explains compensation for low iodine and its breakdown in goiter A
Simulation of a step reduction in maximal TH production per unit thyroid mass, as occurs in iodine deficiency, in the gland‐mass model (without carrying capacities) shows compensation to a euthyroid state: enlarged thyroid, a transient growth in thyrotroph mass and return of hormones to baseline. A model with no gland‐mass changes shows hypothyroidism for the same step change (gray lines).B
Adding carrying capacities to the gland‐mass model limits compensation. Simulations show hypothyroidism for a large step reduction in iodine that causes the enlarged thyroid and thyrotroph mass to approach their carrying capacity. Red lines—model with mass changes, purple lines—model with mass changes and carrying capacity, gray lines—model with no gland‐mass changes. For parameters see Materials and Methods.

To demonstrate this compensation, Fig 3A shows how the model responds to a step‐like reduction in thyroid secretion rate per thyrocyte $b_{\mathrm{TH}}$. Such a reduction is expected for example on low iodine. Low iodine activates rapid compensation mechanisms such as increased iodine uptake, and if these mechanisms saturate, one can expect the TH secretion rate per unit thyroid mass $b_{\mathrm{TH}}$ to drop. As a result, the model shows that the thyroid enlarges over months leading to increased TH secretion, restoring homeostasis (Fig 3A). Such thyroid growth occurs in goiter (Marine & Kimball, 1917; Mariotti & Beck‐Peccoz, 2000). The thyrotroph mass also grows but then returns to baseline over months. A model with no gland‐mass changes (equations (1), (2), (3) only, Fig 3A gray lines) does not show this compensation, and TH levels remain low indefinitely after the step change.

Analogous gland‐mass changes and compensation occur upon high TH levels over weeks. These hyperthyroid conditions lead to thyroid and pituitary mass shrinkage.

**Figure EV2:**
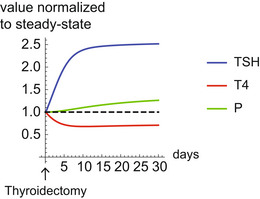
Model dynamics after thyroidectomy After thyroidectomy at time 0, T4 drops and TSH increases, reaching their minimal/maximal concentrations, respectively, after about 7 days. However, thyrotroph mass *P* does not reach its maximal value even after 30 days. All variables are normalized to their healthy steady‐state value.

The compensation paradigm breaks down in cases of extreme perturbations that take the gland masses near their carrying capacity. This can be seen upon a large drop in $b_{\mathrm{TH}}$, which causes the thyroid and pituitary to approach their carrying capacity, breaking the compensation and causing overt hypothyroidism (Figs 3B and EV2). Such perturbations also occur in other thyroid disorders, which we discuss next.

### Delays and hysteresis in thyroid disorders

We now provide more detail on the delays and hysteresis found when modeling thyroid disorders. We begin with Hashimoto's thyroiditis, which is caused by autoimmune attack on the thyroid. We model this by an increased thyrocyte removal rate *a*_*T*_. Hypothyroidism releases the inhibition on TSH secretion and also the inhibition of thyrotroph mass growth. Thus, *P* grows with time during the disease process (Scheithauer *et al*, 1985; Khawaja, 2006; Shukla *et al*, 2019) (Fig 2G). Treating hypothyroidism by supplying external T4 (levothyroxine) leads to normalization of T4 levels. However, the thyrotroph mass *P* takes many weeks to decrease back to baseline, due to the slow turnover rate of thyrotrophs, leading to delays and the hysteresis effect (Figs 1E and 2E).

Hysteresis in the model occurs also in the case of hyperthyroidism due to Graves' disease (Fig 2F). In Graves' disease, autoantibodies activate the TSH receptor. We model this by adding antibody Ab to *TSH* in equations (1) and (4). As a result of the antibody effect, TH levels rise, leading to thyrotroph mass shrinkage (Fig 2H) (Scheithauer *et al*, 1992; Yu & Farahani, 2015). The thyroid mass grows (Dumont *et al*, 1992). After diagnosis, the patient is often treated with antithyroid drugs such as methimazole and carbimazole, which block the production of T4 (reduced $b_{\mathrm{TH}}$ parameter). The drug causes a decline in T4 levels, resulting in increased TSH secretion per thyrotroph in the pituitary; however, for TSH to fully recover in the model, the thyrotroph mass needs to be renewed. This process is much slower, due to the slow turnover of thyrotrophs, explaining the TSH recovery delay after T4 is normalized.

We note that TSH delays in Graves' disease are typically longer than in Hashimoto's thyroiditis. The model can explain this difference. In extended untreated Graves' disease, thyrotroph mass can decline to very low levels, requiring a long recovery time. In contrast, in Hashimoto's thyroiditis the thyrotroph mass growth is limited by a specific carrying capacity, and thus recovery time back to normal *P* is more stereotyped. The delay in Hashimoto's thyroiditis, typically 6 weeks, is therefore rather constant between patients, whereas the delay in Graves' is more variable with about 45% of patients normalizing within 3 months, another 30% normalizing only after 3–6 month, and about 30% that do not normalize within a year (Yu & Farahani, 2015)—possibly because each patient starts treatment at a different thyrotroph mass *P*. The Graves patients who have TSH delays longer than a year often do not recover thyrotroph function, perhaps due to massive and irreparable atrophy of thyrotroph mass *P*.

The essence of hysteresis in the model is thus the changes in pituitary thyrotroph mass *P* during the disease process that take many weeks to recover after treatment begins. Having an estimate for the thyrotroph mass during treatment of thyroid disease can thus help to locate a patient on the hysteresis curve in order to guide treatment strategies. The model provides an estimate for relative changes in *P* based on the hysteresis trajectories. The ratio between the thyrotroph masses is predicted to equal the ratio between the TSH levels on the two trajectories at a given T4 level (Materials and Methods, Fig 2I):

$${\mathrm{TSH}}_{\text{recovery}}/{\mathrm{TSH}}_{\text{disease}}=P_{\text{recovery}}/P_{\text{disease}}$$

This estimate requires TSH tests during the disease process before treatment. If such pre‐illness tests are lacking, the model can be used to estimate the functional mass *P* based on a TRH stimulation test after diagnosis using the following formula:

$$P∼\Delta \mathrm{TSH}\left(1+\frac{T4}{k_{T4}}\right)$$

Where T4 is the FT4 level, and ΔTSH is the rise in TSH following a TRH test, when taking into account TSH turnover rate $a_{\mathrm{TSH}}$ (see Materials and Methods for more details). These two formulae, together with a formula by Dietrich on thyroid functional mass (Dietrich *et al*, 2016), can potentially be used to assess the position of the patient on the hysteresis curve in order to guide treatment.

### Transition between subclinical and clinical diseases

**Figure 4:**
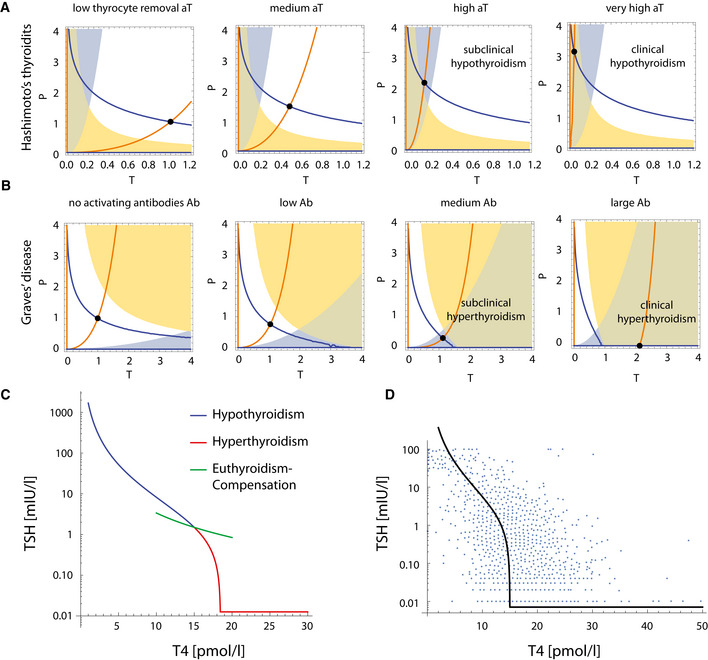
Three‐regime TSH‐T4 relationship can be explained by modeling thyroid disorders A
Hashimoto's thyroiditis transitions from a subclinical state when thyroid removal rate $a_{T}$ is raised moderately, to a clinical hypothyroid state when $a_{T}$ is very high. The blue line is steady‐state *P* at a given *T* (*dP*/*dt* = 0 nullcline), and the orange line is steady‐state *T* at a given *P* (*dT*/*dt* = 0 nullcline). Euthyroid (white), hypothyroid (green), subclinical (hyper‐TSH/normo‐T4, blue), and normo‐TSH/hypo‐T4 (yellow) regions are shown.B
Graves' disease transitions from a subclinical state when autoantibody effect *Ab* is moderate, to clinical hyperthyroidism when *Ab* is high. Nullclines are as in (A), euthyroid (white), hyperthyroid (green), subclinical (hypo‐TSH/normo‐T4, blue) and normo‐TSH/hyper‐T4 (yellow) regions are shown. Note that in B the colored regions change with parameters because they correspond to hormone levels for equations (1–3), not gland masses (Materials and Methods).C
The TSH‐T4 relation in the model shows three regimes. Blue/red curve—analytical solution from the model; Green line—TSH and T4 from the fast timescale relation $\mathrm{TSH}\propto 1/T4^{2}$, shown for T4 values in the normal range.D
Non‐parameterized TSH‐T4 relation assuming a healthy set point of T4 = 15 pmol/L, TSH = 1.5 mIU/L, and thyrotroph carrying capacity of fivefold (Khawaja, 2006), with data from Midgley *et al* (blue points, Materials and Methods) (Midgley *et al*, 2013).

We next use the model to study the TSH–TH relationship. To do so requires an analysis of the transition between subclinical and clinical thyroid disorders (Fig 4A–C).

**Figure EV3:**
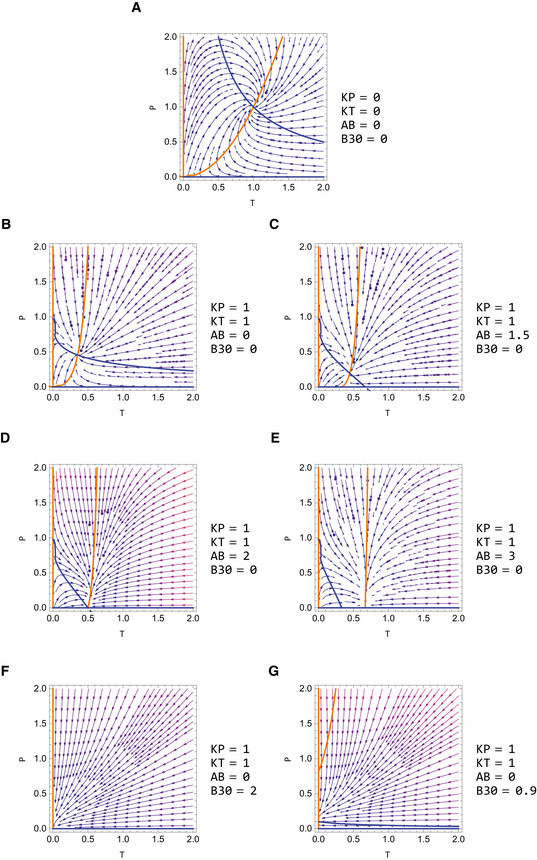
Nullclines and stream plots for the gland‐mass model in different parameter regimes Blue line: *dP*/*dt* = 0, orange line: *dT*/*dt* = 0. The scaled parameters shown here are $K_{T}=\frac{a_{\mathrm{TH}}b_{P}b_{T}}{b_{\mathrm{TH}}a_{P}a_{T}}k_{T},K_{P}=\frac{a_{\mathrm{TRH}}a_{\mathrm{TSH}}a_{T}b_{P}^{2}}{b_{\mathrm{TRH}}b_{\mathrm{TSH}}b_{T}a_{P}^{2}}k_{P}$, $\mathrm{AB}=\frac{b_{T}}{a_{T}}\mathrm{Ab}$, $B_{30}=\frac{a_{P}}{b_{P}a_{\mathrm{TH}}}b_{30}$. For all graphs $K_{x2}=0$. For details about the fixed points in the different regimes, see Appendix Supplementary Text.

To understand the dynamics of the system, we separate the problem into its fast timescale components (equations (1), (2), (3)) and slow timescale components (equations 4 and 5) (Appendix Supplementary Text). We use this separation of timescales to describe the system's slow dynamics by plotting the nullclines dP/dt = 0 and dT/dt = 0 (Materials and Methods), with the fast components at quasi‐steady state. The two nullclines meet at the fixed point of the system (Figs 4A and B, and EV3, Appendix Supplementary Text). This reduces the analysis to two dimensions, which can be readily understood.

We begin with hypothyroidism, the left part of the TSH/T4 relation with high TSH and low T4 (blue line in Fig 4C). The main cause of hypothyroidism is Hashimoto's thyroiditis. We modeled different stages of Hashimoto's thyroiditis by various values of the thyrocyte removal rate $a_{T}$, reflecting different strengths of autoimmune killing. Changing the thyroid removal rate $a_{T}$ only affects one of the nullclines, dT/dt = 0. The fixed point slides along the other nullcline, reducing the problem into a one‐dimensional problem. As the killing rate $a_{T}$ increases, the thyroid gland shrinks and the pituitary gland grows, approaching its carrying capacity (Fig 4A).

When the increase in killing rate is small, steady state TH levels remain in the normal range thanks to elevated TSH levels that precisely compensate for the loss of thyroid mass. This corresponds to *subclinical* Hashimoto's thyroiditis (Fig 4A, blue region). However, when removal rate $a_{T}$ rises above a threshold, the pituitary approaches its carrying capacity and thyroxine levels start to drop below the normal range, causing hypothyroidism (Fig 4A, green region).

We next consider hyperthyroidism (Fig 4B). One of the main causes of hyperthyroidism is Graves' disease. We simulated steady state at different values of the activating antibody term Ab added to TSH in the equations, where Ab is in units of equivalent TSH for its action on the TSH receptor. When antibody levels are low to moderate, the thyrotroph mass shrinks moderately. The effect of the antibodies is compensated by reduced TSH production, so that the thyroid gland mass and TH levels remain unchanged. This corresponds to *subclinical* Graves' disease (Fig 4B, blue region).

**Figure EV4:**
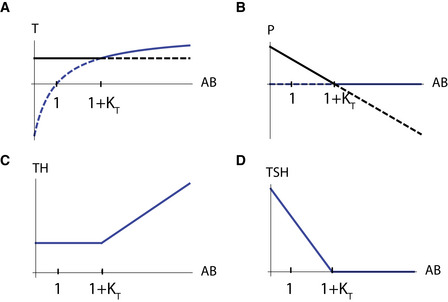
Graves' disease dynamics under thyroid autoantibodies perturbation A–D
Steady‐state values for thyroid gland size *T* (A), pituitary gland size *P* (B), thyroid hormone levels TH (C), and TSH levels (D), when perturbing the levels of the normalized thyroid autoantibodies in the system $\mathrm{AB}=\frac{b_{T}}{a_{T}}\mathrm{Ab}$. Black and blue lines in panels A, B are the two fixed points of the system (i) and (ii), respectively, see Appendix Supplementary Text section “Dependence on antibody parameter in Graves' disease”). When $\mathrm{AB}<1+K_{T}$ the black fixed point is stable (full line) while the blue fixed point is unstable (dashed line). Above this value the fixed point stability switches, in a transcritical bifurcation. When increasing AB up to $1+K_{T}$, *P* shrinks and TSH levels drop, compensating for the autoantibodies stimulatory effect, and allowing for *T* and TH to remain constant (subclinical hyperthyroidism, Figs 4B and EV3C). Crossing this threshold such that $\mathrm{AB}>1+K_{T}$, *P* and TSH become zero and cannot compensate anymore, and thus *T* and TH rise together with AB (clinical hyperthyroidism, Figs 4B and EV3E). $K_{T}$ is the scaled thyroid gland carrying capacity term $K_{T}=\frac{a_{\mathrm{TH}}b_{P}b_{T}}{b_{\mathrm{TH}}a_{P}a_{T}}k_{T}$.

However, when the antibody concentration crosses a threshold $\mathrm{Ab}>\frac{a_{T}}{b_{T}}+\frac{b_{P}a_{\mathrm{TH}}k_{T}}{a_{P}b_{\mathrm{TH}}}$ (Appendix Supplementary Text, Fig EV4), the thyrotroph mass shrinks to zero and compensation breaks down. The thyroid gland begins to grow and with it thyroid hormone levels rise. This corresponds to an L‐shaped TSH–TH relationship, with a sharp decrease in TSH, until it reaches zero, and only then a subsequent rise in TH levels (Fig 4C, red line). A qualitatively similar relation is seen in Fig 1G, as well as in previous studies of the TSH‐T4 relationship (Reichlin & Utiger, 1967; Spencer *et al*, 1990; Midgley *et al*, 2013), where TSH is suppressed to below its detection threshold.

**Figure EV5:**
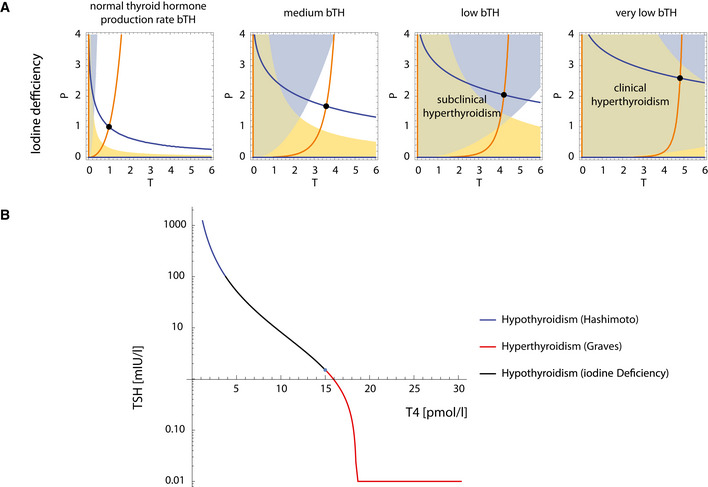
Transitions to hypothyroidism in iodine deficiency conditions A
When thyroid hormone production rate per thyrocyte $b_{\mathrm{TH}}$ is reduced, the thyroid and pituitary gland grow to compensate. However, when $b_{\mathrm{TH}}$ is reduced to extreme values, the system transitions to a subclinical and then a clinical hypothyroid state (Materials and Methods). The blue line is steady‐state *P* at a given *T* (*dP/dt* = 0 nullcline), and the orange line is steady‐state *T* at a given *P* (*dT/dt* = 0 nullcline). Euthyroid (white), hypothyroid (green), subclinical (hyper‐TSH/normo‐T4, blue), and normo‐TSH/hypo‐T4 (yellow) regions are shown.B
The TSH‐T4 relation with TSH and T4 fixed points for Hashimoto's thyroiditis and Graves' disease (blue, red, respectively, same as in Fig 4) and in iodine deficiency conditions, determined for a range of $b_{\mathrm{TH}}$ values (Materials and Methods).

In the case of iodine deficiency, both the thyroid and the pituitary gland grow to compensate for the reduced production rate of thyroid hormones per thyrocyte (reduced $b_{\mathrm{TH}}$). When compensation breaks, the result is first subclinical and then clinical hypothyroidism (Fig EV5).

### The three‐regime TSH‐T4 relationship

Analysis of the model explains why different thyroid disorders, such as Hashimoto's thyroiditis, iodine deficiency, Graves' disease, and toxic nodules, cause changes in TSH and T4 that fall on the same predicted curve. The reason for this is that the formula for the curve does not depend on many of the model parameters and specifically those affected by these disorders. The TSH‐T4 curve is a decreasing rational function that reaches zero at high TH and stays there (see Appendix Supplementary Text for details):

$$\mathrm{TSH}\left(T4\right)=\left\{\begin{array}{cc} A\frac{\left(1-\frac{1}{B}T4\right)}{T4^{2}} & \text{for}\;T4\leq B\\ 0 & \text{for}\;T4>B \end{array}\right.$$

where $A=\frac{b_{\mathrm{TRH}}b_{\mathrm{TSH}}u}{a_{\mathrm{TRH}}a_{\mathrm{TSH}}k_{P}}$ and $B=\frac{b_{P}}{a_{P}}$.

This relationship does not depend on the production and removal rates of thyroid hormone $b_{\mathrm{TH}},a_{\mathrm{TH}}$, on thyrocyte mass growth rate and removal rates $b_{T},a_{T}$, on thyroid gland carrying capacity $k_{T}$ or on antibody level Ab.

Therefore, the model predicts that the same curve for the relation between TSH and T4 remains valid in the conditions that affect any of these parameters. This includes Hashimoto thyroiditis that reduces $a_{T}$; Graves' disease which increases Ab; Changing iodine supply which affects $b_{\mathrm{TH}}$, including iodine deficiency in which $b_{\mathrm{TH}}$ is low; hypersecreting nodules which affect $b_{\mathrm{TH}}$ and $b_{T}$; etc. In all of these conditions, simultaneous measurements of T4 and TSH should fall on the same curve. These parameters would dictate the location of the individual on the curve.

However, conditions which change the pituitary parameters, such as thyrotroph adenoma which affects $b_{P}$ and $b_{\mathrm{TSH}}$, result in a different curve, and hence measurements from these conditions should fall outside the TSH(T4) curve. The same goes for change in the hypothalamic signal *u*, for example, following change in temperature, nutritional state, or health condition.

The relation between TSH and T4 can also be formulated in terms of the steady‐state value of these hormones and *P* in the simple model without carrying capacities (see Appendix Supplementary Text). Using this formulation, we were able to derive a non‐parameterized curve for TSH(T4). To do this, we assumed hormone healthy set point of FT4=15pmol/L, and TSH=1.5mIU/L, and a carrying capacity for the pituitary of 5 times its healthy mass following pituitary measurements from Khawaja (2006). Comparing this curve with data from Midgley *et al* (2013) yields a reasonable agreement (Fig 4D).

In the middle section of the TSH‐T4 relation (Fig 4C), which corresponds to hormones in their normal range, and gland masses far from their carrying capacities, the slow timescale of the glands may play only a secondary role. Recalling the timescale separation between TSH and TH dynamics (hours vs. days), an inverse relation can be explained if TH levels change following tissue demands (Luongo *et al*, 2019), and TSH reacts accordingly. In this case, $\mathrm{TSH}\propto 1/TH^{2}$ (Fig 4C, green line). The slope of this relation should be parallel to the intra‐individual slope of TSH dynamics following T4 treatment, unlike the slopes in the low and high TH regimes, in line with the findings of (Rothacker *et al*, 2016).

## Discussion

This study provides evidence from large‐scale medical records for thyroid hysteresis and for a three‐regime relationship between TSH and T4, and an explanation of these effects by a model of gland‐mass changes in the thyroid axis. The model framework clarifies the dynamics of thyroid disorders on the timescale of months. It explains the phenomena of delays and hysteresis based on changes in pituitary thyrotroph mass during chronic conditions, because such mass changes take many weeks to recover upon treatment. The model also explains the three‐regime relation between TSH and T4 in terms of failed dynamic compensation when gland‐masses approach their carrying capacity, leading to hyperthyroidism or hypothyroidism.

The gland‐mass model also explains the existence of subclinical thyroid disorders, in which TSH is abnormal but T3 and T4 levels are normal, and how these subclinical disorders transition into clinical ones. Subclinical disorders exist because the glands change their mass to fully compensate for disrupted thyroid function. This is an example of the general principle of dynamic compensation (Karin *et al*, 2016). In Hashimoto's thyroiditis, the transition from subclinical to clinical disease occurs when the autoimmune killing rate is so high that thyrotroph mass approaches its carrying capacity, and can no longer compensate by increased TSH secretion for the loss of thyroid function. Such a carrying capacity might be due to the confined anatomical position of the pituitary, together with the need for other pituitary cell types. Some experimental reports show a fivefold increase in pituitary volume in hypothyroidism (Khawaja, 2006), while others show a milder increase. The thyrotroph carrying capacity requires further study; it may not result in observable volume growth if carrying capacity is imposed by other cell types. Alternatively, a low level of thyroid hormones may be required for thyrotroph proliferation, in addition to their effect on other pituitary cell types (Friedrichsen *et al*, 2004).

An additional explanation of subclinical disorders was provided by Dietrich and colleagues, in a model without dynamic compensation (Dietrich *et al*, 2012). Subclinical hypothyroidism resulted from the nonlinear shape of the involved nullclines. However, in the Dietrich model the range of subclinical hypothyroidism was rather small. In a model with dynamic compensation, this effect occurs over a much larger range of parameters, potentially reflecting the high prevalence of subclinical hypothyroidism. Both mechanisms can coexist.

In the case of Graves' disease, the model predicts that the pituitary thyrotroph mass shrinks in order to make less TSH and compensate for the autoantibody activation of TSH receptors. Such a degeneration of thyrotroph mass is seen in pituitary histological samples (Scheithauer *et al*, 1992). This dynamic compensation fails when the antibody effect rises across a threshold at which thyrotroph mass drops near zero, resulting in clinical hyperthyroidism. We predict such a transition also in the case of toxic thyroid nodules. An alternative or complementary explanation for the delay in Graves' disease is the stimulation of an ultra‐short negative feedback loop in which TSH receptor autoantibodies stimulate TSH receptors in the pituitary to inhibit TSH secretion (Prummel *et al*, 2004).

The present model for thyroid disorders provides a basis for the observed three‐regime “log‐linear” TSH–T4 relation. The two extreme regimes correspond to clinical hypothyroidism (Hashimoto's thyroiditis and iodine deficiency) and clinical hypothyroidism (Graves' disease and toxic nodules). The middle regime corresponds to compensated thyroid hormones, composed of a distribution of thyroid set points within a healthy population. The mean slope of the middle region can be explained in the model by two types of effects (i) fast timescale effects such as individual differences in the production and removal rates of hormones, and (ii) slow timescale effects such as differences in the growth and removal rates of the endocrine cells, as may occur during aging. Both sources of variation provide similar predictions for the mid‐regime slope and may both occur physiologically.

The model also explains compensation and its breakdown in the case of goiter due to iodine deficiency. Fast timescale compensation occurs by increased avidity to iodine and other physiological processes. On the slow timescale, once these fast compensation processes have saturated, the model indicates that the thyroid and thyrotroph functional masses both grow, as clinically observed (Marine & Kimball, 1917; Mariotti & Beck‐Peccoz, 2000). Thyroid hormones remain normal until both thyroid and thyrotroph masses approach their carrying capacities. When these carrying capacities are reached, thyroid hormone levels drop to clinical hypothyroidism. This transition to clinical condition occurs when the parameter $b_{\mathrm{TH}}$ in the model, the maximal thyroid hormone secretion rate per thyrocyte, drops below a critical value.

It would be important to measure thyrotroph and thyrocyte masses as well as TSH levels as a function of time after thyroid perturbations in humans and rodents (Pohlenz *et al*, 1999; Nolan *et al*, 2004; Turgeon *et al*, 2017) in order to test the model predictions, such as delays in thyrotroph mass increase after an initial TSH rise. For example, Turgeon *et al* show a reduction in T4 and an elevation of TSH in mice under low iodine diet. It would be interesting to measure the delay in TSH normalization after returning to normal iodine diet and test whether high TSH levels correlate with increased thyrotroph mass.

The dynamic compensation mechanism by changes in mass in equations (4) and (5) is an example of a more general feedback‐control strategy known in engineering as integral feedback, as in the pioneering work of El‐Samad and Khammash (El‐Samad *et al*, 2002; Alon, 2019). When the glands are far from their carrying capacity, equations (4) and (5) guarantee that steady state can only be achieved at certain values of TH and TSH, namely $\mathrm{TSH}=f^{-1}\left(a_{T}/b_{T}\right)$ and $\mathrm{TH}=b_{P}/a_{P}$. These set points are independent of all of the fast timescale parameters in equations (1–3). Therefore, changes in hormone production and degradation parameters, such as changes in iodine supply, are expected to be compensated. On the long timescale of weeks‐months, TH and TSH are guaranteed to reach a set point that is defined only by the proliferation and death rate of the thyrocytes and thyrotrophs, respectively.

This compensation explains, at least in part, how an individual's set point of TSH and TH is kept within a relatively limited range (Andersen *et al*, 2002; Wartofsky & Dickey, 2005), even in face of sizable changes in the parameters. For example, iodine nutritional intake can drop or exceed the recommended level by an order of magnitude before there is a clinical effect (Trumbo *et al*, 2001; National Research Council *et al*, 2005). The model also predicts full compensation, after a transient of a few months, for moderate changes in hypothalamic input such as temperature and nutritional states. Compensation should also occur to changes in blood volume, which can be described as changes in the hormone secretion parameters (Karin *et al*, 2016). The model can explore changes during gestation. Interestingly, the finding of coordinated *P* and *T* volumes changes (Otani *et al*, 2021) sets constraints on which parameter group might change during gestation (Appendix Supplementary Text).

The present study focused on the dynamics of free T4; future work can address the dynamics of free T3 (FT3) concentration. Dietrich and colleagues observed increased FT3 concentration and increased calculated deiodinase capacity (SPINA‐GD) in beginning hypothyroidism, but this was dependent on the amount of thyroid tissue (Hoermann *et al*, 2014, 2015a, 2016; Midgley *et al*, 2015). Therefore, it may be mediated by dynamic compensation.

The complex dynamics and hysteresis of thyroid hormones challenge the treatment of thyroid disorders, especially in a fraction of the population where the dynamics seem not to easily converge (Dietrich, 2015). The present model might, therefore, guide future studies on improved treatment. One way that the model might help is to provide estimates of the effective thyrotroph mass of a patient, the “hidden variable” at the root of hysteresis. This is perhaps analogous to the benefits in the field of diabetes gained by estimates of insulin resistance and beta‐cell function from insulin and glucose test results using models such as HOMA (Matthews *et al*, 1985). Such a formula for thyroid total function was developed by Dietrich *et al* (Dietrich *et al*, 2016). The present study suggests a formula for relative pituitary thyrotroph mass based on TRH tests. One can, therefore, envisage using the present model, perhaps together with additional measurements, to design optimal treatment over time for returning a given patient to the euthyroid condition. For example, estimating thyrotroph mass accumulation rate during treatment in Graves' disease can help to predict the time to recovery and thus help to choose between a conservative to an ablative approach for treatment.

## Materials and Methods

### Clalit medical record dataset

The Clalit dataset contains the anonymized electronic health records of 3.45 million individuals per year, on average (Balicer & Afek, 2017). Diagnosis codes were acquired from both primary care and hospitalization records and were mapped to the International Classification of Diseases, 9th revision (ICD9) coding system. The full study protocol was approved by the Clalit Helsinki Committee RMC‐1059‐20.

### Population‐level hysteresis curves

Hysteresis curves (Fig 1E and F) were acquired by studying simultaneous TSH and FT4 blood tests from individuals above 20 years old with Hashimoto's thyroiditis (ICD9 code 245.2) or Graves' disease (ICD9 code 242.0), which purchased medications (levothyroxine sodium for Hashimoto's thyroiditis or Thiamazole/Propylthiouracil/Propranolol for Graves' disease) within 90 days prior or up to 3‐year post‐diagnosis. For each individual, all the available simultaneous TSH and FT4 measurements were collected. Measurements were categorized as “pre‐treatment” or “post‐treatment” where the onset of treatment was set to the earliest time that diagnosis was found or medication was bought, up to 90 days prior to diagnosis. Mean FT4 was evaluated for semi‐equal‐sized bins of TSH—bin size varied due to the discrete nature of measurement values. Overall, we acquired 77,246 pre‐treatment and 249,527 post‐treatment (TSH, FT4) measurements of Hashimoto patients, in bins of size 724–2,357 tests per bin, and 26,327 pre‐treatment and 105,812 post‐treatment measurements of Graves patients in bins of size 1,028–2001 tests per bin. In Fig 1E (hysteresis in the context of Hashimoto thyroiditis), we show measurements in the normal/hypothyroid range (TSH > 0.5). In Fig 1F (hysteresis in the context of Graves' disease), we show measurements in the normal/hyperthyroid range (TSH < 5). Error bars in Fig 1E and F represent the standard error of the mean. In Fig 1G, we show the pre‐treatment curves that appear in Fig 1E and F together with the normal FT4/TSH range that together create the inverse FT4–TSH relation.

### Gland‐mass model simulations

For simulations, we used the following parameter set based on the experimental literature. Hormone removal rates are 1/6 min for TRH, 1/1 h for TSH, and 1/7 days for T4, thyrotroph mass turnover = 1/30 days, and thyrocyte mass turnover = 1/30 days, (Dumont *et al*, 1992; Nolan *et al*, 1998; Mariotti & Beck‐Peccoz, 2000; Dietrich *et al*, 2016). Hormone and gland production rates and carrying capacity terms were chosen to give a steady state equal to 1. To simulate a step down in iodine (Fig 3), normalized secretion rate per thyrocyte ($b_{\mathrm{TH}}$) was reduced by half.

Hysteresis in Hashimoto's thyroiditis (Fig 2E and G) was simulated by increasing $a_{T}$ by fivefold from ${a_{T}}_{\text{basal}}=\frac{1}{30}\text{days}$ to $a_{T}=5{a_{T}}_{\text{basal}}$ for 200 days until reaching a new steady state. The treatment state was simulated by adding external T4 for 100 days to equation (1) to reach the original steady state. The external T4 added was calibrated to yield the original steady state. Carrying capacity terms for the glands were set to be $k_{T}=0,k_{P}=1$, reflecting the assumption that in this case, the thyroid gland is far from its carrying capacity and the pituitary approaches its carrying capacity. Hysteresis in Graves’ disease (Fig 2F and H) was simulated by adding *Ab* to TSH in equations (3) and (5) for 100 days, with *Ab* = 5 in units of the steady‐state TSH concentration and then reducing $b_{\mathrm{TH}}$ and setting *Ab* = 0 during the treatment phase for an additional 500 days, to simulate the effect of antithyroid medications. The new $b_{\mathrm{TH}}$ value in the treatment period was calibrated to yield the original steady state. In this case, carrying capacity terms were $k_{T}=1,k_{P}=0$, reflecting the assumption that here the pituitary gland is far from its carrying capacity and the thyroid gland approaches its carrying capacity.

Since we aimed to formulate an easily interpretable and analytically solvable model to allow understanding of the basic principles of the system, we ignore several aspects such as the regulation of deiodinase cellular expression levels and the TSH‐T3 shunt (Berberich *et al*, 2018; Luongo *et al*, 2019). Detailed description of the model can be found in the Appendix Supplementary Text.

### Computation of nullcline curves for the gland‐mass model

To compute the nullclines in Figs 4A and B, and in Figs EV3 and EV5, we used the separation of timescales in the model between the hormone turnover times and the slower gland turnover times. We thus assumed that the hormones are at steady state and computed the nullclines by setting dT/dt=0 or dP/dt=0. This gives the following nullclines:

*T*' = 0:

$$\left\{P=\frac{{\left(T+B_{30}\left(1-K_{T}T\right)\right)}^{2}}{{\left(1-K_{T}T\right)}^{2}}\left(\frac{1}{1-K_{T}T-K_{X2}}-\mathrm{AB}\right)\;\text{OR}\;T=0\right\}$$

*P*′ = 0:

$$\left\{T=\left(1-B_{30}-K_{P}P\right)\frac{\left(1+\mathrm{AB}\;K_{X2}\left(1+\frac{P}{{\left(1-K_{P}P\right)}^{2}}\right)\right)}{\mathrm{AB}+\frac{P}{{\left(1-K_{P}P\right)}^{2}}}\;\text{OR}\;P=0\right\}$$

where $K_{T}=\frac{a_{\mathrm{TH}}\;b_{P}\;b_{T}}{b_{\mathrm{TH}}\;a_{P}\;a_{T}}k_{T},K_{P}=\frac{a_{\mathrm{TRH}}\;a_{\mathrm{TSH}}\;a_{T}{\;b}_{P}^{2}}{\;b_{\mathrm{TRH}}\;b_{\mathrm{TSH}}\;b_{T}{\;a}_{P}^{2}}k_{P}$, $\mathrm{AB}=\frac{b_{T}}{a_{T}}\mathrm{Ab}$, and $B_{30}=b_{30}\frac{a_{P}}{a_{3}b_{P}}$ where *b*_30_ is the external levothyroxine supply.

In the simple case of $\mathrm{AB}=B_{30}=K_{X2}=0$, (no autoantibodies, no external thyroid hormone supply, and a linear response function for TH *f*(*TSH*) = *TSH*), the nullclines take the simple form of:

$$\left\{P=\frac{T^{2}}{{\left(1\;-\;K_{T}\;T\right)}^{3}}\;\text{OR}\;T=0,T=\frac{{\left(1\;-\;K_{P}\;P\right)}^{3}}{P}\;\text{OR}\;P=0\right\}$$

For details, see Appendix Supplementary Text.

We used the following parameters: Removal rates for the hormones and glands as indicated above. Hormone production rates were chosen to give steady‐state hormone values of FT4 = 15 pmol/l, TSH = 1.5 mIU/l and steady‐state glands masses of $P_{\text{st}}=1$, $T_{\text{st}}=1$. Thyroid and pituitary gland carrying capacities were estimated as 5.5, 5.3 times their normal volume following data from Liu *et al* (2013) and Khawaja (2006), respectively. The hypo‐TSH/hyper‐TSH regions were defined as the range of *P*, *T* values that result in TSH <0.5, TSH >5, respectively. The hypo‐T4/ hyper‐T4 regions were defined as the range of *P*, *T* values that result in T4 < 10, T4 > 20, respectively. The healthy (T4, TSH) st. st. point was defined as (15 pmol/l, 1.5mIU/l). In Fig 4A, thyroid removal rate $a_{T}$ was varied as follows: $a_{T}$=[${a_{T}}_{\text{basal}}$, $2{a_{T}}_{\text{basal}}$, $5{a_{T}}_{\text{basal}}$, $15{a_{T}}_{\text{basal}}$] where ${a_{T}}_{\text{basal}}=\frac{1}{30}\;\text{days}$. In Fig 4B, thyroid antibodies level Ab was varied as follows: $\mathrm{Ab}=\left[0,0.5{\mathrm{TSH}}_{\mathrm{st}},1.2{\mathrm{TSH}}_{\mathrm{st}},2{\mathrm{TSH}}_{\mathrm{st}}\right]$ where ${\mathrm{TSH}}_{\mathrm{st}\;-\;\mathrm{st}}$ is the healthy TSH level. In Fig EV5A, $b_{\mathrm{TH}}$ values were taken to be $\left[b_{\mathrm{TH}},\;0.1b_{\mathrm{TH}},\;0.04b_{\mathrm{TH}},\;0.02b_{\mathrm{TH}}\right]$, where $b_{\mathrm{TH}}$ is the thyroid hormone production rate per thyrocyte under normal iodine conditions. All simulations were performed using Wolfram Mathematica 12.2. Code is provided online at GitHub https://github.com/yaelkorem-weizmann/HPT_glandmass_model.

### Analytical TSH‐T4 relation and comparison to data

The TSH‐T4 curve in Fig 4C and D was computed from solving the steady state of the model equations for TRH, TSH, and *P* (equations 2, 3, 5) (Appendix Supplementary Text). This gives the following piecewise rational function:

$$\mathrm{TSH}\left(T4\right)=\left\{\begin{array}{cc} \frac{b_{\mathrm{TRH}}b_{\mathrm{TSH}}u}{a_{\mathrm{TRH}}a_{\mathrm{TSH}}k_{P}}\frac{\left(1-\frac{a_{P}}{b_{p}}T4\right)}{T4^{2}} & \text{for}\;T4\leq \frac{b_{P}}{a_{P}}\\ \mathrm{TSH}\left(T4\right)=0 & \text{for}\;T4>\frac{b_{P}}{a_{P}} \end{array}\right.$$

This relation can also be formulated in terms of the steady‐state values of T4, TSH, and *P* in a simple model without carrying capacities, $T4_{0},\mathrm{TS}H_{0},P_{0}$ (Appendix Supplementary Text):

$$\mathrm{TSH}\left(T4\right)=\left\{\begin{array}{cc} \alpha \frac{\left(1-\mathrm{\beta T}4\right)}{T4^{2}} & \text{for}\;T4\leq \frac{1}{\beta }\\ 0 & \text{for}\;T4>\frac{1}{\beta } \end{array}\right.$$

where $\alpha =\frac{\mathrm{TS}H_{0}\;T4_{0}^{2}}{P_{0}\;k_{P}},\beta =\frac{1}{T4_{0}}$ and $\mathrm{TS}H_{0}=\frac{a_{T}}{b_{T}},T4_{0}=\frac{b_{P}}{a_{P}},P_{0}=\frac{a_{\mathrm{TRH}}\;a_{\mathrm{TSH}}\;a_{T}\;{b_{P}}^{2}}{{a_{P}}^{2}\;\;b_{\mathrm{TRH}}\;b_{\mathrm{TSH}}\;b_{T}\;u}$.

To compare the analytical solution to data, we calculated α,β using typical parameters from the literature. The healthy set point of the hormones is $\mathrm{TS}H_{0}=1.5\;\mathrm{mIU}/L$, $T4_{0}=15\;\text{pmol}/L$. We use a thyrotroph carrying capacity of 5 times the healthy thyrotroph mass $P_{0}=5\;\mathrm{Kp}$, following pituitary measurements from Khawaja *et al* that showed reduction of up to 80% in pituitary volume in hypothyroid patients following treatment (Khawaja, 2006).

Data in Fig 4D were graphically extracted from Midgley *et al* (2013) using WebPlotDigitizer software. The density of the data points was adjusted for visual clarity.

### Formula for functional thyrotroph mass

To derive a formula for the functional mass of the pituitary P based on a TRH test, we use equation (2), with Michaelis–Menten terms for TSH stimulation by TRH and for TSH suppression by T4:

$$\text{dTSH}/\mathrm{dt}=b_{\mathrm{TSH}}P\frac{\mathrm{TRH}}{\mathrm{TRH}+k_{\mathrm{TRH}}}\frac{k_{T4}}{k_{T4}+T4}\;-\;a_{\mathrm{TSH}}\mathrm{TSH}$$

To estimate *P* requires three measurements (i) basal TSH levels $\mathrm{TS}H_{\text{basal}}$, (ii) TSH after TRH stimulation (usually after Δt ∼ 30 min) $\mathrm{TS}H_{\text{stimulated}}$, and (iii) basal T4 levels $T4_{\text{basal}}$. Given that the TRH test uses a saturating amount of TRH, $\mathrm{TRH}\gg k_{\mathrm{TRH}}$, we approximate the temporal derivative using the difference divided by Δt:

$$\frac{\mathrm{TS}H_{\text{stimulated}}\;-\;\mathrm{TS}H_{\text{basal}}}{\Delta t}=b_{\mathrm{TSH}}P\left(1+\frac{k_{T4}}{T4}\right)\;-\;a_{\mathrm{TSH}}\mathrm{TS}H_{\text{basal}}$$

We define ΔTSH, the rise in TSH following the TRH test when taking into account TSH turnover rate $a_{\mathrm{TSH}}$, as:

$$\Delta \mathrm{TSH}=\mathrm{TS}H_{\text{stimulated}}\;-\;\mathrm{TS}H_{\text{basal}}\left(1+a_{\mathrm{TSH}}\Delta t\right),$$

Therefore: $P=\frac{1}{b_{\mathrm{TSH}}\mathrm{\Delta t}}\text{ΔTSH}\left(1+\frac{T4}{k_{T4}}\right)$

Note that $b_{\mathrm{TSH}}$, the maximal TSH secretion rate per thyrotroph per unit TRH and unit T4, is a characteristic of the system that we assume is constant, and Δt is a test parameter, the time for the TSH measurement after TRH stimulation, which can be calibrated to improve the accuracy of this formula for practical use.

Therefore, the formula $P∼\text{ΔTSH}\left(1+\frac{T4}{k_{T4}}\right)$ is an estimate for the thyrotroph functional mass (i.e., the secretory capacity of *P*) that can be used to estimate the change in thyrotroph mass of an individual during the treatment of thyroid diseases.

### Ratio between thyrotroph masses on two hysteresis trajectories at a given T4 level is equal to the ratio between the TSH levels

Consider a hysteresis loop that is composed of two trajectories: (1) before diagnosis and (2) after diagnosis and during treatment. We define TSH levels at a given T4 level on these trajectories, $\mathrm{TS}H_{1}$ and $\mathrm{TS}H_{2}$, respectively (Fig 2I). In this section, we provide a mathematical proof that the ratio between these TSH values ${\mathrm{TSH}}_{1}/{\mathrm{TSH}}_{2}$ is equal to the thyrotroph mass ratio at these points $P_{1}/P_{2}$.

From equation (3), for a constant environmental signal u, TRH is uniquely defined by TH:

$$\mathrm{TRH}=\frac{b_{\mathrm{TRH}}u}{a_{\mathrm{TRH}}}\frac{1}{\mathrm{TH}}$$

From equation (2), at steady state:

$$\mathrm{TSH}=\frac{b_{\mathrm{TSH}}}{a_{\mathrm{TSH}}}P\frac{\mathrm{TRH}}{\mathrm{TH}}=\frac{b_{\mathrm{TSH}}}{a_{\mathrm{TSH}}}P\frac{1}{{\mathrm{TH}}^{2}}$$

Therefore, for a constant thyroid hormone level $TH_{0}$:

$${\mathrm{TSH}}_{1}=\frac{b_{\mathrm{TSH}}}{a_{\mathrm{TSH}}}P_{1}\frac{1}{{{\mathrm{TH}}_{0}}^{2}},\;\mathrm{TS}H_{2}=\frac{b_{\mathrm{TSH}}}{a_{\mathrm{TSH}}}P_{2}\frac{1}{{{\mathrm{TH}}_{0}}^{2}}$$

Hence: $\frac{{\mathrm{TSH}}_{1}}{{\mathrm{TSH}}_{2}}=\frac{P_{1}}{P_{2}}$

## Author contributions

**Uri Alon:** Conceptualization; supervision; funding acquisition; methodology; writing – original draft; writing – review and editing. **Yael Korem Kohanim:** Conceptualization; software; investigation; visualization; methodology; writing – original draft; writing – review and editing. **Tomer Milo:** Conceptualisation; writing – review and editing. **Moryia Raz:** Conceptualisation; writing – review and editing. **Omer Karin:** Conceptualisation; writing – review and editing. **Alon Bar:** Conceptualisation; writing – review and editing. **Avi Mayo:** Conceptualisation; software; investigation; writing – review and editing. **Netta Mendelson Cohen:** Data curation; conceptualisation; writing – review and editing. **Yoel Toledano:** Conceptualisation; writing – review and editing.

In addition to the CRediT author contributions listed above, the contributions in detail are:

Conceptualization: YKK, UA, TM, MR, OK, AB, AM, NMC, YT; Methodology: YKK, UA; Investigation: YKK, AM; Software: YKK, NMC, AM; Writing—original Draft: YKK, UA; Writing—review & editing: YKK, TM, MR, OK, AB, AM, NMC, YT, UA; Visualization: YKK; Data curation: NMC; Funding Acquisition: UA; Supervision: UA.

## Disclosure and competing interests statement

The authors declare that they have no conflict of interest.

## Supporting information



Appendix

Expanded View Figures PDF

## Data Availability

Modeling computer scripts: GitHub (https://github.com/yaelkorem‐weizmann/HPT_glandmass_model).
