# Supplementary material for: Dynamics of thyroid diseases and thyroid‐axis gland masses
Source: Mol Syst Biol. 2022 Aug 8;18(8):e10919. doi: 10.15252/msb.202210919 (PMC9358402; doi:10.15252/msb.202210919)
Supplement: Supplementary file 1 — Appendix [file MSB-18-e10919-s003.pdf]

Appendix to “Dynamics of thyroid diseases and thyroid-axis gland masses”

Yael Korem Kohanim, Tomer Milo, Moriya Raz, Omer Karin, Alon Bar, Avi Mayo, Netta Mendelson Cohen, Yoel Toledano, Uri Alon

Contents

Appendix Supplementary Text: Mathematical Model for gland-mass dynamics in the thyroid axis 2

Model definition 2

Steady state of the simple model 3

Model rescaling 4

The model shows exact adaptation only when glands are far from their carrying capacities.... 4

Computation of nullclines 5

Stability analysis 6

Dependence on antibody parameter in Graves' disease 8

The relation between the steady-states of TSH and T4 9

Changes in fetal thyroid and pituitary volume during gestation 10

Trans-differentiation from other cell types into thyrotrophs 10

Rise of TSH mRNA over days following thyroidectomy 12

Figure S1 13

Appendix Supplementary Text: Mathematical Model for gland-mass dynamics in the thyroid axis

Model definition

To Model HPT dynamics, we use the following equations:

$$(1)\ \frac{dx_1(t)}{dt} = \frac{b_1\, u}{x_3(t)} - a_1\, x_1(t)$$

$$(2)\ \frac{dx_2(t)}{dt} = \frac{b_2\, P(t) x_1(t)}{x_3(t)} - a_2\, x_2(t)$$

$$(3)\ \frac{dx_3(t)}{dt} = b_{30} + b_3\, T(t) \frac{(Ab + x_2(t))}{1 + k_{x2}(Ab + x_2(t))} - a_3\, x_3(t)$$

$$(4)\ \frac{dT(t)}{dt} = T(t)(b_T \frac{(Ab + x_2(t))}{1 + k_{x2}(Ab + x_2(t))}(1 - k_T\, T(t)) - a_T)$$

$$(5)\ \frac{dP(t)}{dt} = P(t)(\frac{b_P}{x_3(t)}(1 - k_P\, P(t)) - a_P)$$

Where the variables are:

Table S1: Model variables

x_1	Thyrotropin-releasing hormone (TRH) concentration
x_2	Thyroid-stimulating hormone (TSH) concentration
x_3	Thyroid hormone (TH) concentration, T4 and T3
T	Thyrocyte mass
P	Pituitary thyrotroph mass

We summarize the model parameters in the following table:

Table S2: Model parameters

u	Environmental input
a_1, a_2, a_3	TRH, TSH, TH removal rate respectively
a_T, a_P	Thyroid/pituitary cell removal rate, respectively
b_1, b_2, b_3	TRH, TSH, TH production rate, respectively
b_T, b_P	Thyroid thyrocyte/ Pituitary thyrotroph mass growth rate, respectively
$k_T\ , k_P$	Carrying capacity terms for the thyroid/pituitary gland respectively. Note that when $k_T = 0, k_P = 0$, this mean that the glands do not have carrying capacities and can grow indefinitely. Otherwise, T,P maximal masses are $1/k_T$, $1/k_P$ respectively.
Ab	TSH-receptor stimulating antibodies
b_{30}	External thyroid hormone supply.

k_{x2}	Michaelis-Menten coefficient for the response function of TH

Equation (1) describes TRH (x_1) dynamics. TRH production is stimulated by hypothalamic input *u* which represents the integrated effect of cues including temperature, illness and nutritional states, and is inhibited by thyroid hormones T3 and T4 (x_3).

Equation (2) describes TSH (x_2), which is secreted by pituitary thyrotrophs *P*, stimulated by TRH (x_1) and inhibited by TH (x_3).

Equation (3) describes thyroid hormone dynamics (x_3), which is secreted by the thyroid gland, whose functional mass is T, when stimulated by TSH (x_2).
b_{30} represents external thyroid hormone supply. We use this term when simulating levothyroxine treatment of Hashimoto's thyroiditis. Under normal conditions $b_{30} = 0$. Ab represents TSH-receptor stimulating antibodies. We use this term when modeling Grave's disease. Under normal conditions Ab = 0. k_{x2} is a Michaelis-Menten coefficient for the response function of TH. We use this parameter to explore the effect of using a MM response function for TH, which is more realistic than a linear function. We found that this choice does not affect the qualitative results of the model.

x_1, x_2, x_3 are removed at rates a_1, a_2, a_3 respectively.

Equation (4) for the thyroid functional mass has a removal/turnover term – $a_T\, T$ and a mass growth term activated by TSH (x2). $(1 - k_T\, T)$ is a carrying capacity term, that allows positive growth rate term only for $T < 1/k_T$. The thyroid growth rate is also affected by activating antibodies Ab if they exist, and is generally described by a MM function with MM coefficient k_{x2}.

Equation (5) describes the dynamics of pituitary thyrotroph mass *P*, which follows a similar balance of growth and removal, where we assume that the main control of mass growth is a suppressive effect by TH (x_3). a_P is the removal rate of thyrotrophs, and the carrying capacity (maximal mass) of thyrotrophs is $1/k_P$.

Under normal conditions, both glands are far from their carrying capacities, $kT \cong kP \cong 0$. When modeling Hashimoto's thyroiditis, the relevant carrying capacity is that of the pituitary gland (because the thyroid gland is destructed and is thus small), therefore we approximate $kT \cong 0$. When modeling Graves' disease, the relevant carrying capacity is that of the thyroid gland (the pituitary is small due to the negative TH feedback), therefore we approximate $kP \cong 0$.

Steady state of the simple model

In general, the steady-state of the model cannot be computed explicitly. However, we can compute it for a simple model that represents the healthy state, in which there are no TSHR antibodies and no external thyroid hormone supply ($Ab = 0, b_{30} = 0$), the glands are far from their carrying capacities (i.e. infinite carrying capacity $kT = kP = 0$), and x_3 depends approximately linearly on x_2 ($k_{x2} = 0$). In this case the steady-state is:

$$\{x_1 = \frac{a_P\, b_1\, u}{a_1\, b_P}, x_2 = \frac{a_T}{b_T}, x_3 = \frac{b_P}{a_P}, P = \frac{a_1\, a_2\, a_T\, {b_P}^2}{a_P^2\, b_1\, b_2\, b_T\, u}, T = \frac{a_3\, b_P\, b_T}{a_P\, a_T\, b_3}\}$$

Model rescaling

For the sake of clarity, we begin by a scaling the variables by their steady-state values from the simple model (i.e. $Ab = k_{x2} = b_{30} = k_T = k_P = 0$):

$$\{x_1 \to \frac{a_P\, b_1\, u}{b_P\, a_1}, x_2 \to \frac{a_T}{b_T} x_2, x_3 \to \frac{b_P}{a_P} x_3, P \to \frac{b_P^2\, a_1\, a_2\, a_T}{{a_P}^2\, b_1\, b_2\, b_T\, u} P, T \to \frac{a_3\, b_P\, b_T}{b_3\, a_P\, a_T} T\}$$

In addition, we redefine the parameters:

$$Ab = \frac{a_T}{b_T} AB, \qquad k_{x2} = \frac{b_T}{a_T} K_{X2}, \qquad k_T = \frac{a_P\, a_T\, b_3}{b_P\, b_T\, a_3} K_T,$$

$$k_P = \frac{{a_P}^2\, b_1\, b_2\, b_T}{{b_P}^2 a_1\, a_2\, a_T} K_P, \qquad b_{30} = \frac{a_3\, b_P}{a_P} B_{30}$$

Therefore, the model equations transform to the dimensionless form:

$$(6)\ \frac{1}{a_1} x_1'(t) = \frac{1}{x_3(t)} - x_1(t)$$
$$(7)\ \frac{1}{a_2} x_2'(t) = P(t) \frac{x_1(t)}{x_3(t)} - x_2(t)$$
$$(8)\ \frac{1}{a_3} x_3'(t) = B_{30} + \left(T(t) \frac{AB + x_2(t)}{1 + K_{X2}(AB + x_2(t))} - x_3(t)\right)$$
$$(9)\ \frac{1}{a_T} T'(t) = T(t) \left(\frac{AB + x_2(t)}{1 + K_{X2}(AB + x_2(t))} (1 - K_T\, T(t)) - 1\right)$$
$$(10)\ \frac{1}{a_P} P'(t) = P(t) \left(\frac{1}{x_3(t)} \left(1 - K_P\, P(t)\right) - 1\right)$$

Note that with the choice $AB = K_{X2} = B_{30} = K_P = K_T = 0$ we recover the simple model.

The model shows exact adaptation only when glands are far from their carrying capacities

Note that in this simple model, there is exact adaptation for x2 and for x3: the values of these variables depend only on the production and removal rates of the glands. This happens since the glands P and T function as integral feedback controllers, as can be seen in the equations for the simple model below:

$$\frac{dT(t)}{dt} = T(t)(x_2 - 1)$$
$$\frac{dP(t)}{dt} = P(t) \left(\frac{1}{x_3(t)} - 1\right)$$

The gland masses change so that they compensate for any change in the model parameters, except for the production and removal rates of the gland, guaranteeing that $x_2 = x_3 = 1$. Note that x_2 and x_3 were rescaled, so that in terms of the original variables $x_2 = \frac{a_T}{b_T}$ and $x_3 = \frac{b_P}{a_P}$. Therefore, this simple model cannot account for cases of hypo- or hyperthyroidism.

Adding carrying capacity terms break this perfect adaptation, since the glands can no longer fully compensate for change in the hormone secretion or removal parameters (Fig EV1). In this case, the glands function as non-ideal integral controllers, where the non-ideal aspect comes naturally from the finite carrying capacity.

Adding a carrying capacity term for the pituitary gland breaks the adaptation of x2 (TSH). This can be seen by solving the model steady-state with carrying capacity only for the pituitary, $Ab = AB = K_{X2} = B_{30} = K_P = 0\,, KT \neq 0$:

$$\{x_2 = 1 + K_T, x_3 = 1\}$$

Note that K_T depends on many of the model parameters- $K_T = \frac{a_3\, b_P b_T}{b_3 a_P a_T} K_T$, so that x2 depends now not only on thyrotroph production and removal rates, but also on TH production and removal rates, thyrocyte production and removal rates, and the carrying capacity of the thyroid gland.

Adding a carrying capacity term for the thyroid gland breaks the adaptation of x3 (TH). This can be seen by solving the model steady-state with carrying capacity only for the thyroid, $Ab = B30 = KX2 = AB = KT = 0, KP \neq 0$:

$$\{x_2 = 1, x_3 = \frac{-1 + \sqrt{1 + 4K_P}}{2K_P}\}$$

Again, KP depends on the model parameters, $K_P = \frac{a_1\, a_2\, a_T\, {b_P}^2}{b_1\, b_2\, b_T\, a_P^2} k_P$, making x3 sensitive to change in these parameters.

Computation of nullclines

To compute the nullclines $dT/dt = 0, dP/dt = 0$ we use separation of time scales, using the fact that hormone turnover times are much faster than the gland turnover times. We separate the model to the hormone "fast" equations (6)-(8), and the gland "slow" equations (9)-(10).

We first solve the steady-states for the hormone equations. The solution as a function of P and T can be represented as the roots of a third degree polynomial. For the sake of clarity, we express x1 and x3 using x2, and write an implicit equation for x2:

$$x_1 = \frac{\sqrt{x_2}}{\sqrt{P}}$$

$$x_3 = \frac{\sqrt{P}}{\sqrt{x_2}}$$

$$B_{30} + \left(T \frac{AB + x_2}{1 + K_{X2}(AB + x_2)} - \sqrt{\frac{P}{x_2}} \right) = 0$$

With this, the "slow time scale" system is composed of two ODE's for P and T and one implicit algebraic equation for x2:

(11) $$\frac{1}{a_T}T'(t) = T(t)\left(\frac{AB+x_2(t)}{1+K_{X2}(AB+x_2(t))}(1-K_T\,T(t))-1\right)$$

(12) $$\frac{1}{a_P}P'(t) = P(t)\left(\sqrt{\frac{x_2(t)}{P(t)}}(1-K_P\,P(t))-1\right)$$

(13) $$B_{30}+\left(T\frac{AB+x_2}{1+K_{X2}(AB+x_2)}-\sqrt{\frac{P}{x_2}}\right)=0$$

In order to find the nullclines we need to solve the equations dT/dt=0 and dP/dt=0. One trivial solution is T=0, P=0.

In order to find the non-trivial solution we solve the equations T'=0 and P'=0 for x2, and substitute the solution into the implicit equation for x2. This gives the following nullclines:

T'=0:

(14) $$\left\{P=\frac{(T+B_{30}(1-K_T\,T))^2}{(1-K_T\,T)^2}\left(\frac{1}{1-K_T\,T-K_{X2}}-AB\right)\quad OR\quad T=0\right\}$$

P'=0:

(15) $$\left\{T=(1-B_{30}-K_PP)\frac{\left(1+AB\,K_{X2}\left(1+\frac{P}{(1-K_PP)^2}\right)\right)}{AB+\frac{P}{(1-K_PP)^2}}\quad OR\quad P=0\right\}$$

The nullclines in the simple case (Fig. EV3A), i.e. $B_{30}=K_{X2}=AB=0$, takes the simple form of:

$$\left\{\boldsymbol{P=\frac{T^2}{(1-K_T\,T)^3}\quad OR\quad T=0},\qquad \boldsymbol{T=\frac{(1-K_P\,P)^3}{P}\quad OR\quad P=0}\right\}$$

Stability analysis

In order to validate the stability of the slow system we need to find the signs of the eigenvalues of the Jacobian of the reduced system (P,T) (equations (11)-(13)) at the steady state.

The system has four fixed points: (i) one at T>0, P>0, (ii) one at T>0, P=0, and (iii) one at T=0, P=0, and (iv) one at T=0, P>0.

We illustrate the different stability regimes in Figure EV3.

(i) **We first inspect the solution at T>0, P>0** (Fig EV3B,C). To calculate the stability of this fixed point we need to calculate the partial derivative of x2 with respect to P and T.

$$x_2^{(1,0)}(P,T)=\frac{1}{\frac{P}{x_2}+\frac{2\sqrt{P}\,T\sqrt{x_2}}{(1+K_{X2}(AB+x_2))^2}}$$

$$x_2^{(0,1)}(P,T)=-\frac{2x_2^{3/2}(AB+x_2)(1+K_{X2}(AB+x_2))}{2T\,x_2^{\ 3/2}+\sqrt{P}(1+K_{X2}(AB+x_2))^2}$$

Calculating the Jacobian and substituting the above, we find that the Jacobian is:

$$J = \begin{pmatrix} at\left(-1+\frac{(1-KT\,T)(AB+x2)}{1+KX2(AB+x2)}-\frac{T(AB+x2)(2x2^{3/2}+KT\sqrt{P}(1+KX2(AB+x2))^2)}{(1+KX2(AB+x2))(2T\,x2^{3/2}+\sqrt{P}(1+KX2(AB+x2))^2)}\right) & -\frac{at\,T(-1+KT\,T)x2}{2\sqrt{P}T\,x2^{3/2}+P(1+KX2(AB+x2))^2} \\ \frac{ap\sqrt{P}(-1+KP\,P)x2(AB+x2)(1+KX2(AB+x2))}{2Tx2^{3/2}+\sqrt{P}(1+KX2(AB+x2))^2} & ap\left(-1-KP\sqrt{P}\sqrt{x2}+\frac{(1-KP\,P)\sqrt{x2}}{\sqrt{P}}+\frac{(-1+KP\,P)Tx2^2}{2\sqrt{P}T\,x2^{3/2}+P(1+KX2(AB+x2))^2}\right) \end{pmatrix}$$

It is difficult to find the steady-state solution of P,T and x2. However, from (11),(12) we can find a simple expression that connects the steady-state solution of P, T and x2 in this system:

$$\left\{\frac{(1-K_T\,T)(AB+x_2)}{1+K_{X2}(AB+x_2)}=1, \qquad (1-K_P\,P)\sqrt{\frac{x_2}{P}}=1\right\}$$

Note that from this we learn that a non-negative solution exists only for (1-KT T)>0 and (1-KP P)>0. Substituting this into the Jacobian *J* we find:

$$J = \begin{pmatrix} -\frac{at\,T(AB+x2)(2x2^{3/2}+KT\sqrt{P}(1+KX2(AB+x2))^2)}{(1+KX2(AB+x2))(2T\,x2^{3/2}+\sqrt{P}(1+KX2(AB+x2))^2)} & -\frac{at\,T(-1+KT\,T)x2}{2\sqrt{P}T\,x2^{3/2}+P(1+KX2(AB+x2))^2} \\ \frac{ap\sqrt{P}(-1+KP\,P)x2(AB+x2)(1+KX2(AB+x2))}{2Tx2^{3/2}+\sqrt{P}(1+KX2(AB+x2))^2} & ap\left(-KP\sqrt{P}\sqrt{x2}+\frac{(-1+KP\,P)Tx2^2}{2\sqrt{P}T\,x2^{3/2}+P(1+KX2(AB+x2))^2}\right) \end{pmatrix}$$

For this steady state solution to be stable, the eigenvalues must all have negative real part. A necessary and sufficient condition for this in 2D systems is that the trace is negative and the determinant is positive. Inspection of *J* shows that this is indeed the case.

Therefore, when this fixed point exists it is stable.

(ii) **We next inspect the stability of the second fixed point at P=0, T>0** (Fig EV3D,E)**.** This fixed point can be computed explicitly:

$$\left\{x_1=\frac{K_T(1+AB\,K_{X2})}{-1+AB+B_{30}\,K_T-AB\,K_{X2}+AB\,B_{30}\,K_T\,K_{X2}}, \qquad x_2=0, \right.$$
$$x_3=\frac{-1+AB+B_{30}\,K_T-AB\,K_{X2}+AB\,B_{30}\,K_T\,K_{X2}}{K_T(1+AB\,K_{X2})}, \qquad P=0,$$
$$\left. T=\frac{-1+AB-AB\,K_{X2}}{AB\,K_T}\right\}$$

This fixed point is positive only if $AB > \frac{1}{1-K_{X2}}$. If KX2=0 this condition is reduced to **AB>1**.

The eigenvalues of the Jacobian at this fixed point are:

$$\left\{-1,-1,-1,at+AB\,at(-1+2KX2), \frac{a_P(1+K_T-B_{30}K_T+AB(-1+(1+K_T-B_{30}K_T)K_{X2}))}{-1+AB+B_{30}K_T+AB(-1+B_{30}K_T)K_{X2}}\right\}$$

The eigenvalues are negative provided that:

$$AB > \frac{1+K_T-B_{30}\,K_T}{1-K_{X2}-K_T\,K_{X2}+B_{30}\,K_T\,K_{X2}}$$

In the simple case when there is no external thyroid hormone supply B30=0, and KX2=0, this condition is reduced to **AB>1+KT**.

(iii) **Third, we inspect the fixed point at (P=0, T=0)** (Fig EV3F)**.** This fixed point can be explicitly computed:

$$\{x_1 = \frac{1}{B_{30}}, x_2 = 0, x_3 = B_{30}, P = 0, T = 0\}$$

Note that this fixed point exists only if there is an external thyroid hormone supply B30>0. The eigenvalues in this case are:

$$\{a_P(-1 + \frac{1}{B_{30}}), -1, -1, -1, (-1 + AB)a_T\}$$

The eigenvalues are negative providing that **AB<1 and B30>1**.

(iv) Finally, we inspect the stability of the fourth fixed point at **T=0, P>0** (Fig EV3G). This point can be computed explicitly as:

$$\{x_1 = \frac{1}{B_{30}}, x_2 = \frac{1 - B_{30}}{B_{30}^2 K_P}, x_3 = B_{30}, P = \frac{1 - B_{30}}{K_P}, T = 0\}$$

Note that this fixed point exists only if there is an external thyroid hormone supply B30>0 and that the pituitary gland has a finite carrying capacity $K_P > 0$.
In this case, the eigenvalues of the Jacobian are:

$$\{\frac{a_P(-1 + B_{30})}{B_{30}}, -1, -1, -1, a_T(-1 + AB + \frac{1 - B_{30}}{B_{30}^2\, K_P})\}$$

They are negative providing that **B30<1 and** $\boldsymbol{AB < 1 - \frac{1 - B_{30}}{B_{30}^2 K_P}}$

Dependence on antibody parameter in Graves' disease

In this section we analytically explore the model dynamics under perturbation of the Ab parameter in Graves' diseases. We use the results of the stability analysis done above. In Graves' disease, the pituitary gland is far from its carrying capacity $P \ll \frac{1}{k_P}$, and there is no external thyroid hormone supply $b_{30} = 0$. We also assume for simplicity that $kx2 = 0$.

Therefore, the system has two fixed points in the P-T plane (Fig EV4A,B, Fig 4B):

i. $\left\{x_1 = 1, x_2 = 1 - AB + K_T, x_3 = 1, P = 1 - AB + K_T, T = \frac{1}{1+K_T}\right\}$

ii. $\left\{x_1 = \frac{K_T}{AB-1}, x2 = 0, x3 = \frac{AB-1}{K_T}, P = 0, T = \frac{1}{K_T}(1 - \frac{1}{AB})\right\}$

The black lines in Fig EV4A,B represent fixed point (i), blue lines represent fixed point (ii).

The parameter AB determines the sign and the stability of these two fixed points. When $AB < 1$, we get that only fixed point (i) is positive and stable (black full line in Fig EV4A,B). Above this value, when $1 < AB < 1 + KT$, another unstable positive fixed point arises - fixed point (ii) (blue dashed line in Fig EV4A,B). Up to this point, the thyroid gland T has a constant value: $T =$

$\frac{1}{1+KT}$, which does not depend on AB. The value of thyroid hormones TH (x3) does not depend on AB as well (Fig EV4C). This is possible since the values of P and TSH (x2) do depend on AB - when AB is increased, P shrinks and TSH levels drop (Fig EV4D), compensating for the stimulatory effect of AB. However, when $AB > 1 + KT$, fixed point (i) becomes negative and unstable (black dashed line in Fig EV4A,B) while fixed point (ii) becomes stable (blue full line in Fig EV4A,B), a situation called a transcritical bifurcation. At this point, the pituitary gland has shrunk to zero, TSH=0, and the stimulatory effect of autoantibodies Ab cannot be compensated anymore. From this point on, thyroid gland size T and thyroid hormone level TH rise gradually with Ab: $T = \frac{1}{K_T}(1 - \frac{1}{AB}), x_3 = \frac{AB-1}{K_T}$. These dynamics explain the occurrence of subclinical hyperthyroidism, in which TSH levels are low though thyroid hormone levels are kept normal (Fig 4B).

The relation between the steady-states of TSH and T4

In this section we compute the relation between TSH and T4, here denoted x2 and x3, that appears in Figure 4. Note that in this model, we ignore the complexity of T4 and T3 relation, and treat them as one variable x3.

From the steady-state of the equations for x1, x2 and P (1),(2),(5) we get:

$$x_1 = \frac{b_1 u}{a_1 x_3}$$

$$\begin{cases} P = \frac{b_P - a_P\, x_3}{b_P\, k_P} & x_3 < \frac{b_P}{a_P} \\ P \;=\; 0 & x_3 > \frac{b_P}{a_P} \end{cases}$$

Substituting x1 and P in x2 we get the relation x2(x3):

$$\begin{cases} x_2(x_3) = \frac{b_1\, b_2\, u}{a_1\, a_2\, k_P} \frac{(1 - \frac{a_P}{b_P} x_3)}{x_3^2} & x_3 \le \frac{b_P}{a_P} \\ x_2(x_3) \;=\; 0 & x_3 > \frac{b_P}{a_P} \end{cases}$$

Note that the relation between x2 and x3 can also be written in terms of the steady-state value of these variables and the variable P in the simple model without carrying capacities, (see Appendix section "Steady state of the simple model"):

In terms of these values, we can write:

$$\begin{cases} x_2(x_3) = \alpha \frac{(1 - \beta\, x_3)}{x_3^2} & x_3 \le \frac{1}{\beta} \\ x_2(x_3) \;=\; 0 & x_3 > \frac{1}{\beta} \end{cases}$$

Where $\alpha = \frac{x2_0\, x3_0{}^2}{P_0\, k_P}, \beta = \frac{1}{x30}$, and $x2_0 = \frac{a_T}{b_T}, x3_0 = \frac{b_P}{a_P}, P_0 = \frac{b_P{}^2 a_1\, a_2\, a_T}{a_P{}^2\, b_1\, b_2\, b_T u}$.

Note that $x2_0, x3_0$ are not the steady-state for the full system with carrying capacities.

Assuming that in the normal healthy set-point the glands are far from their carrying capacities, we can thus compute α, β. We consider a normal set-point of FT4 $x3_0 = 15pmol/L$, and of TSH $x2_0 = 1.5mIU/L$, and we normalize the pituitary mass units so that in the normal set point $K_P = \frac{P_0}{5}$ following measurements from Khawaja et al. that showed reduction of up to 80% in pituitary volume in hypothyroid patients following treatment. We thus derived a non-parameterized curve for x2(x3). Comparing this curve with data from Midgley et al (Midgley et al., 2013) yield a reasonable agreement (Fig 4D).

Changes in fetal thyroid and pituitary volume during gestation

A recent study found both pituitary and thyroid volume to correlate positively to gestational age in preterm and term infants but to correlate negatively to chronological age (Otani et al., 2021). Changes in volume during gestation may result from many different physiological effects. If we assume that the main change is due to the thyroid axis modeled here, the coordinated changes in P and T offer interesting constraints on which parameter group changes during gestation.

The thyroid and pituitary set points in the model, assuming they are not close to their carrying capacities, are:

$$T = \frac{a_{TH} b_P b_T}{b_{TH} a_P a_T}, P = \frac{a_{TRH} a_{TSH} a_T {b_P}^2}{b_{TRH} b_{TSH} b_T {a_P}^2 u}$$

The change in T,P set-point before and after birth suggested by Otani et al. can thus reflect change in parameters in utero and after birth. Interestingly, P and T are observed to change together: they grow together before birth, and shrink together after birth. This fact sets a constraint on which parameter is responsible for this transition, because this parameter should affect P and T in the same direction. Therefore, the model combined with the findings of Otani et al. suggests that the parameter responsible for the opposite trends in gland volume change before and after birth is either b_P and/or a_P: the pituitary thyrotroph production and removal rates. This would be the case if thyrotroph proliferation rate is higher in utero than after birth. Note that these pituitary parameters dictate the volume accumulation of both glands – pituitary and thyroid. Another interesting prediction is the pituitary volume is more sensitive to changes in bp and ap due to the quadratic dependence on b_P and a_P, compared to the linear dependency of T on these parameters. This is in line with Otani et al that showed larger relative volume changes for P than for T for the same time period.

Trans-differentiation from other cell types into thyrotrophs

In this section, we consider other mechanisms for thyrotroph mass accumulation other than thyrotroph proliferation or growth. This includes trans-differentiation from other pituitary cell types (Horvath et al., 1990; Radian et al., 2003) and/or redifferentiation from dedifferentiated thyrotrophs (Wang et al., 2014).

Mathematically, we account for these effects by adding to the equation that describes the pituitary thyrotroph mass dynamics a source term inhibited by thyroid hormone levels.

(16) $\frac{dP(t)}{dt} = \frac{b_{Pcross}}{x3(t)}(1 - k_P\,P(t)) + P(t)(\frac{b_P}{x_3(t)}(1 - k_P\,P(t)) - a_P)$

The other model equations (1)-(4) remain the same.

We find that adding this term leaves the results of this analysis qualitatively similar.

As before, we rescale the variables by their steady-state value in the simple case in which the glands are far from their carrying capacities (i.e. infinite carrying capacity kT=kP=0), there are no TSHR antibodies and no external thyroid hormone supply (Ab=0, b30=0), and x3 depends approximately linearly on x2 (kx2=0). However here we keep the parameter that describes the thyrotroph proliferation/growth rate bp in the equations, so latter it can be compared to bp_{cross}, the rate of thyrotroph mass accumulation from non-thyrotroph cells:

$$\{x_1 \to \frac{a_P\, b_1\, u}{a_1}\,, x_2 \to \frac{a_T}{b_T}x_2, x_3 \to \frac{1}{a_P}x_3, P \to \frac{a_1\, a_2\, a_T}{{a_P}^2\, b_1\, b_2\, b_T\, u}P, T \to \frac{a_3\, b_T}{b_3\, a_P\, a_T}T\}$$

Rescaling the variables, we define new variables:

$$Ab = \frac{a_T}{b_T}AB, \qquad k_{x2} = \frac{b_T}{a_T}K_{X2}, \qquad k_T = \frac{a_P\, a_T\, b_3}{b_T\, a_3}K_T,$$

$$kP = \frac{ap^2\, b1\, b2\, bt}{a1\, a2\, at}KP, \qquad b30 = \frac{a3}{ap}B30, \qquad bpcross \to \frac{a1\, a2\, at\, BPcross}{ap^2\, b1\, b2\, bt\, u}$$

The new scaled equations are:

(17) $\frac{1}{a_1}x_1'(t) = \frac{1}{x_3(t)} - x_1(t)$

(18) $\frac{1}{a_2}x_2'(t) = P(t)\frac{x_1(t)}{x_3(t)} - x_2(t)$

(19) $\frac{1}{a_3}x_3'(t) = B_{30} + \left(T(t)\frac{AB + x_2(t)}{1 + K_{X2}(AB + x_2(t))} - x_3(t)\right)$

(20) $\frac{1}{a_T}T'(t) = T(t)\left(\frac{AB + x_2(t)}{1 + K_{X2}(AB + x_2(t))}(1 - K_T\,T(t)) - 1\right)$

(21) $\frac{1}{a_P}P'(t) = \frac{B_{Pcross} + b_p\,P(t)}{x_3(t)}\left(1 - K_P\,P(t)\right) - P(t)$

Computing the steady-state for this system in the simple case described above, we find that the steady-state of x3 (thyroid hormone) is now dependent on the thyrotroph mass growth rate b_P, and on the trans-differentiation term bp_{cross} (Figure S1A) . However, if the sum of rates $bp + b_{Pcross}$ remains constant, x3 does not change. In other words, the overall rate of thyrotroph mass accumulation matters, but not their specific source.

When P approaches its carrying capacity, i.e. $K_P > 0$, the symmetry between the trans-differentiation model to the original model starts to break (Figure S1B). In this case, replacing thyrotroph proliferation bp with thyrotroph synthesis from non-thyrotroph source b_{Pcross}, has a similar effect on thyroid hormone levels but with scaling: Higher b_{Pcross} values map to lower bp values. Therefore, higher trans-differentiation rate is required to maintain a similar thyrotroph mass.

Next, we tested the effect of adding a trans-differentiation term on the TSH(TH) relationship (Figure S1C). The models differ in their prediction for the TSH(TH) relation in hyperthyroidism. The original model predicts a sharp drop to very low TSH values even in subclinical hyperthyroidism with thyroid hormone levels that are only slightly above normal. However, the new model predicts a gradual drop in TSH with the rise in TH levels.

This difference stems from the behaviors of the two models in the regime of small thyrotroph mass. In the original model, when thyrotroph mass is equal to zero it cannot be restored again. Therefore, in Graves' disease, for high enough antibodies level, the model has a fixed point at zero thyrotroph mass: $\left\{x_1 = \frac{K_T}{AB-1}, x_2 = 0, x_3 = \frac{AB-1}{K_T}, P = 0, T = \frac{1}{K_T}\left(1 - \frac{1}{AB}\right)\right\}$ (See Appendix section “Dependence on antibody parameter in Graves' disease” and Figure EV4). In this regime, the thyrotroph mass and TSH levels cannot compensate for the antibody stimulation, leading to increased TH levels and enlarged of thyroid mass. However, in the trans-differentiation model, thyrotrophs mass is generated even when it is low, due to the non-thyrotroph sources. Therefore, the fixed point at P=0 is lost. More data is needed to conclusively differentiate between these predictions.

Nullcline analysis and stream plot simulations reveal that the full dynamics, and not only the steady-state, remain qualitatively similar (Fig S1D-F). The nullclines for the model when $K_{X2} = B_{30} = AB = 0$ are:

$$\{T^2 = P(1 - K_T\, T)^3 \quad \text{OR} \quad T = 0, \qquad P^2 = \frac{(B_{Pcross} + b_P P)^3 (1 - K_P\, P)^3}{T}\}$$

However, a major difference between the models is that the original model has a nullcline at P=0, while the new model doesn't. This leads to the difference described above, in the regime of high thyroid mass T and small thyrotroph mass P.

Rise of TSH mRNA over days following thyroidectomy

Nolan et al provide evidence of a rise in TSH mRNA over days after thyroidectomy (Nolan et al., 2004). The present model captures this effect, based on the lifetime of TH. This lifetime is seven days in humans and is thought to be on the order of a few days in mice. Thus, after thyroidectomy, thyroid hormone levels fall gradually, reaching halfway down after its half-life. In this timescale of days, TSH mRNA expression in the thyrotrophs will be gradually de-repressed, and their levels would rise (Fig EV2). In contrast, thyrotroph mass will grow on a longer time scale of about a month. Figure EV2 illustrates this point: After thyroidectomy at time 0, T4 drops and TSH increases, reaching their minimal/maximal concentrations respectively after about 7 days. However, thyrotroph mass P does not reach its maximal point even after 30 days.

Figure S1

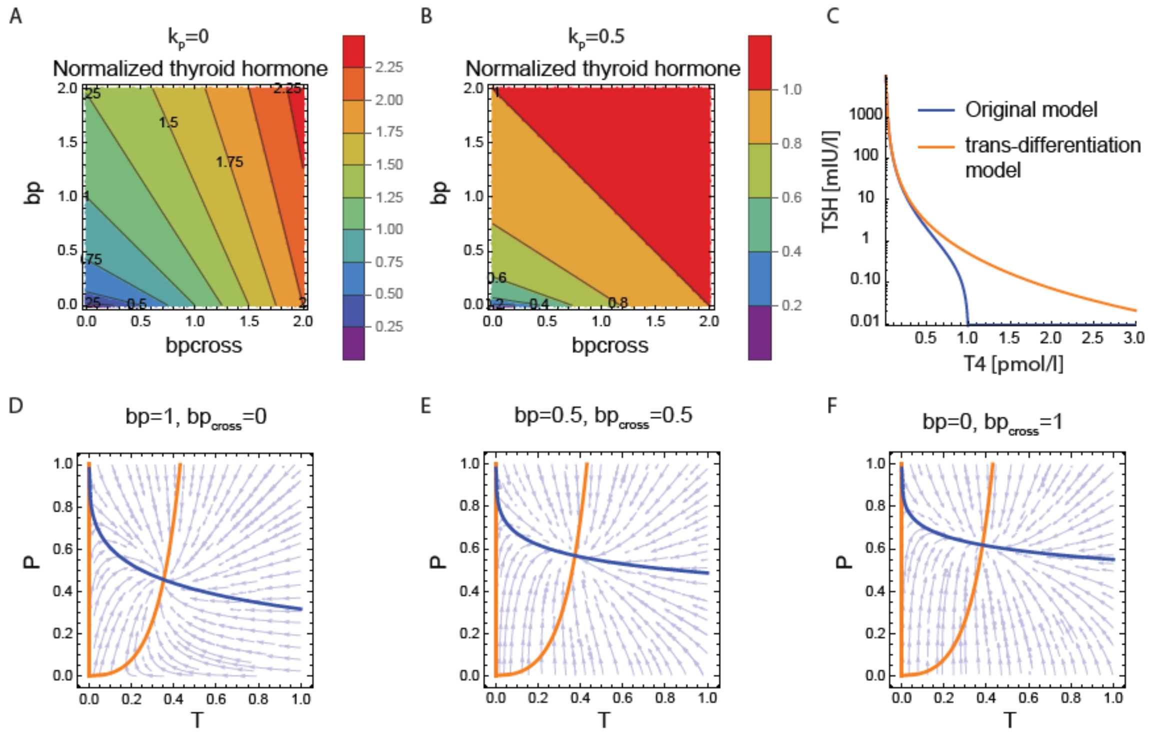

Figure S1: Model with trans-differentiation term has similar dynamics in the euthyroid and hypothyroid regimes but not in hyperthyroidism. (A,B) Steady-state thyroid hormone levels in a model with trans-differentiation to thyrotrophs from non-thyrotroph cell types for a range of thyrotroph proliferation/growth rates b_p and trans-differentiation rates $b_{p_{cross}}$. When $b_{p_{cross}} = 0$ the original model is restored. (A) P is far from its carrying capacity $k_P = 0$ (B) $k_P = 0.5$. (C) TSH(TH) relation in the trans-differentiation model compared to the original model. (D,E,F) Nullclines and stream plots for the trans-differentiation model for different b_p,$b_{p_{cross}}$ values.
