# Supplementary material for: Dynamics of thyroid diseases and thyroid‐axis gland masses
Source: Mol Syst Biol. 2022 Aug 8;18(8):e10919. doi: 10.15252/msb.202210919 (PMC9358402; doi:10.15252/msb.202210919)
Supplement: Supplementary file 2 — Expanded View Figures PDF [file MSB-18-e10919-s001.pdf]

Expanded View Figures

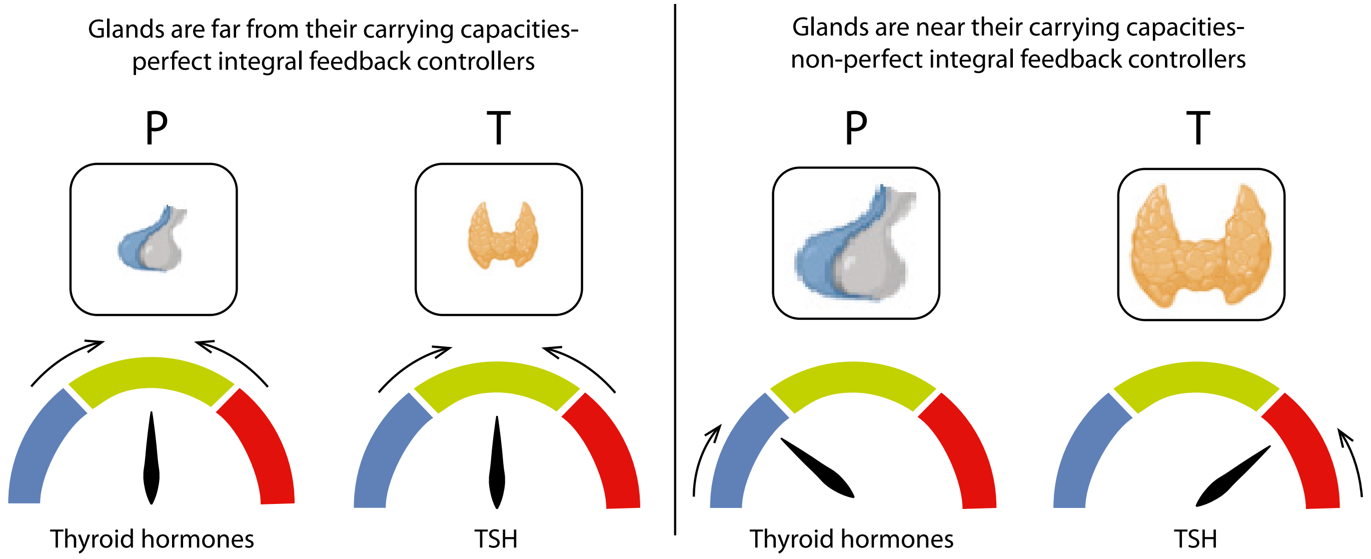

Figure EV1. Thyrotroph mass *P* and thyrocyte mass *T* as integral feedback controllers for thyroid hormones and TSH, respectively.

Left: When the glands are far from their carrying capacities, they provide perfect integral feedback control, keeping the hormones at their homeostatic set points. Right: When the glands approach their carrying capacities, they serve as imperfect integral controllers and can only partially prevent the hormones deviation from their set points.

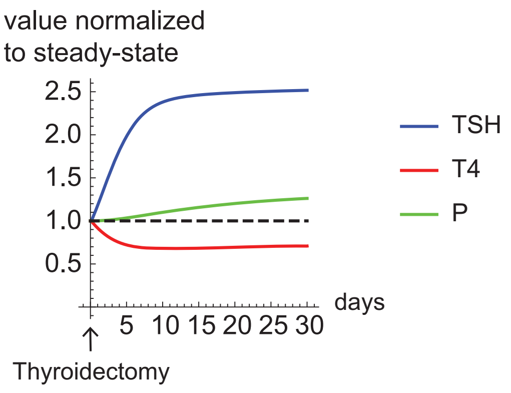

Figure EV2. Model dynamics after thyroidectomy.

After thyroidectomy at time 0, T4 drops and TSH increases, reaching their minimal/maximal concentrations, respectively, after about 7 days. However, thyrotroph mass *P* does not reach its maximal value even after 30 days. All variables are normalized to their healthy steady-state value.

Figure EV3. Nullclines and stream plots for the gland-mass model in different parameter regimes.

Blue line: $dP/dt = 0$, orange line: $dT/dt = 0$. The scaled parameters shown here are $K_T = \frac{a_{TH} b_P b_T}{b_{TH} a_P a_T} k_T$, $K_P = \frac{a_{TRH} a_{TSH} a_T b_P^2}{b_{TRH} b_{TSH} b_T a_P^2} k_P$, $AB = \frac{b_T}{a_T} Ab$, $B_{30} = \frac{a_P}{b_P a_{TH}} b_{30}$. For all graphs $K_{x2} = 0$. For details about the fixed points in the different regimes, see Appendix Supplementary Text.

▶

© 2022 The Authors

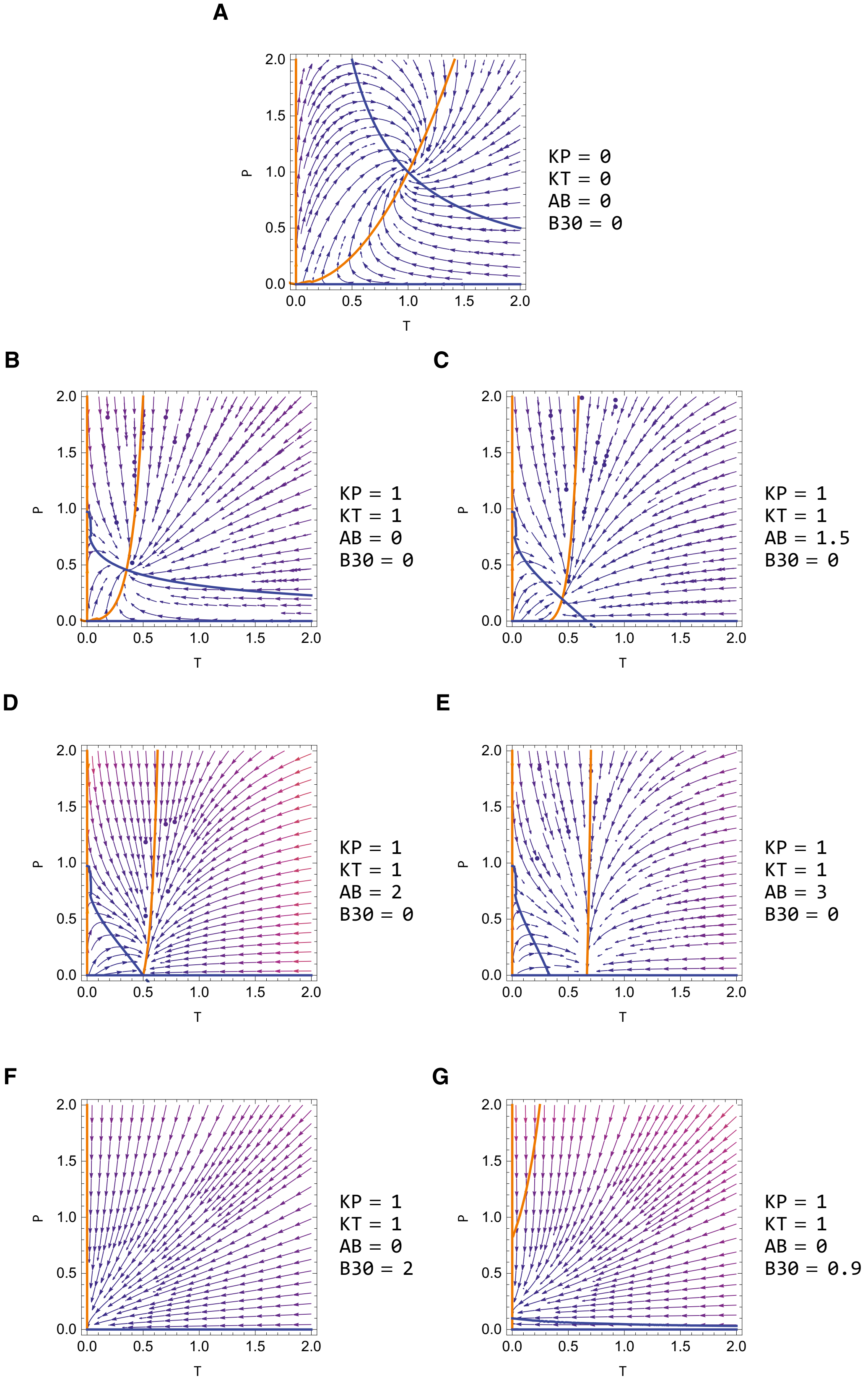
A
KP = 0
KT = 0
AB = 0
B30 = 0
B
KP = 1
KT = 1
AB = 0
B30 = 0
C
KP = 1
KT = 1
AB = 1.5
B30 = 0
D
KP = 1
KT = 1
AB = 2
B30 = 0
E
KP = 1
KT = 1
AB = 3
B30 = 0
F
KP = 1
KT = 1
AB = 0
B30 = 2
G
KP = 1
KT = 1
AB = 0
B30 = 0.9
P
T
0.0
0.5
1.0
1.5
2.0

Figure EV3.

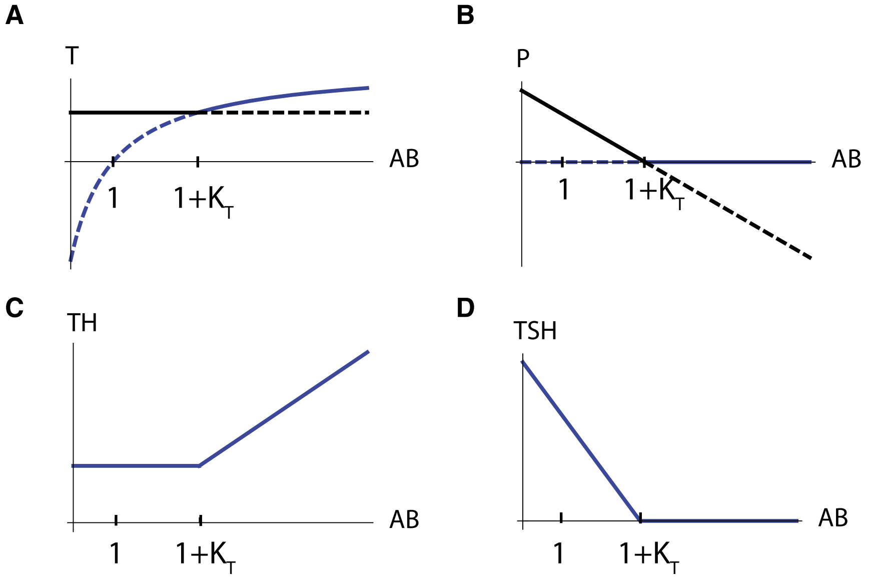

Figure EV4. Graves' disease dynamics under thyroid autoantibodies perturbation.

A–D Steady-state values for thyroid gland size *T* (A), pituitary gland size *P* (B), thyroid hormone levels TH (C), and TSH levels (D), when perturbing the levels of the normalized thyroid autoantibodies in the system $AB = \frac{b_T}{a_T}Ab$. Black and blue lines in panels A, B are the two fixed points of the system (i) and (ii), respectively, see Appendix Supplementary Text section "Dependence on antibody parameter in Graves' disease"). When $AB < 1 + K_T$ the black fixed point is stable (full line) while the blue fixed point is unstable (dashed line). Above this value the fixed point stability switches, in a transcritical bifurcation. When increasing AB up to $1 + K_T$, *P* shrinks and TSH levels drop, compensating for the autoantibodies stimulatory effect, and allowing for *T* and TH to remain constant (subclinical hyperthyroidism, Figs 4B and EV3C). Crossing this threshold such that $AB > 1 + K_T$, *P* and TSH become zero and cannot compensate anymore, and thus *T* and TH rise together with AB (clinical hyperthyroidism, Figs 4B and EV3E). K_T is the scaled thyroid gland carrying capacity term $K_T = \frac{a_{TH} b_P b_T}{b_{TH} a_P a_T} k_T$.

© 2022 The Authors

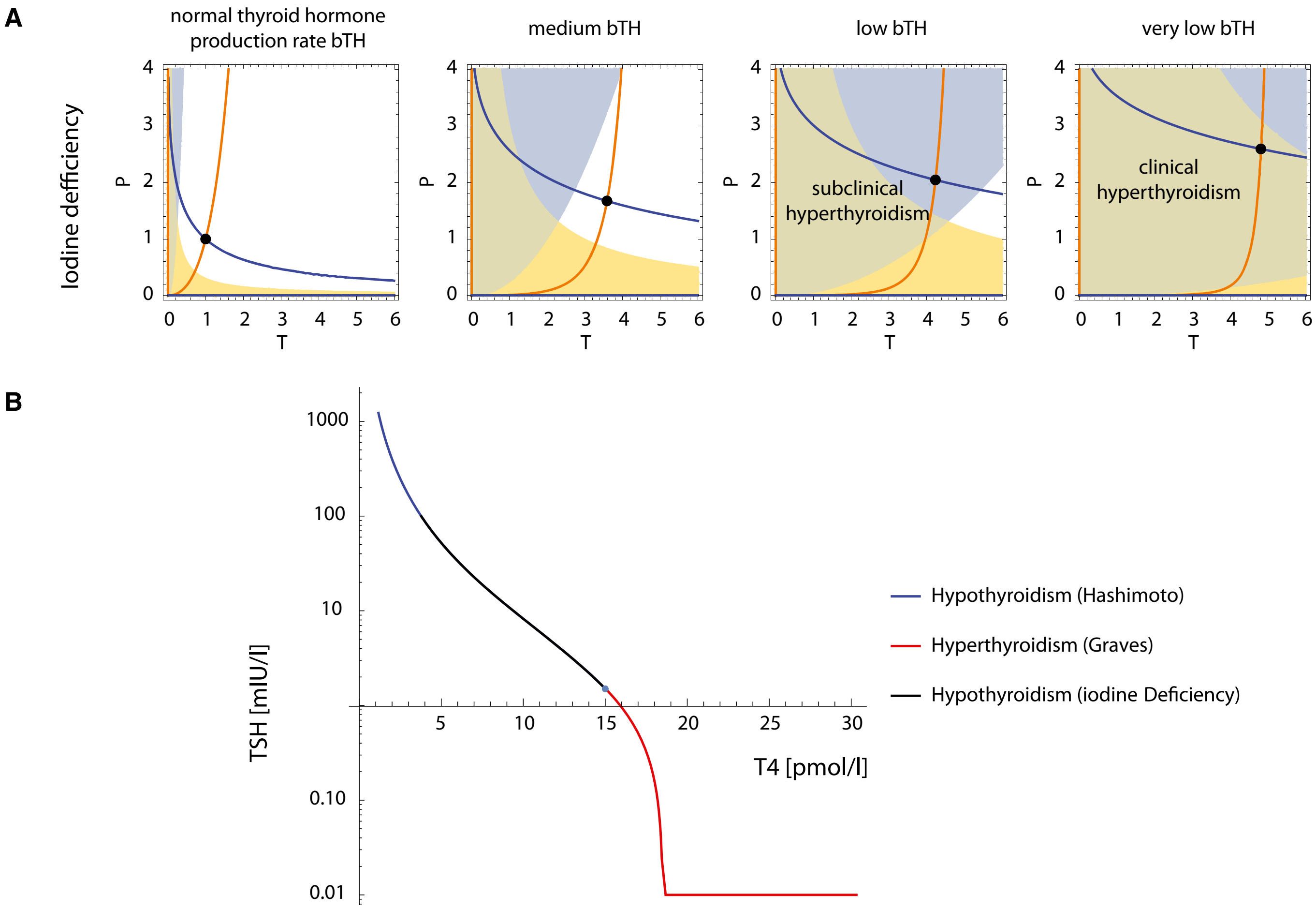

Figure EV5. Transitions to hypothyroidism in iodine deficiency conditions.

A When thyroid hormone production rate per thyrocyte b_{TH} is reduced, the thyroid and pituitary gland grow to compensate. However, when b_{TH} is reduced to extreme values, the system transitions to a subclinical and then a clinical hypothyroid state (Materials and Methods). The blue line is steady-state P at a given T ($dP/dt = 0$ nullcline), and the orange line is steady-state T at a given P ($dT/dt = 0$ nullcline). Euthyroid (white), hypothyroid (green), subclinical (hyper-TSH/normo-T4, blue), and normo-TSH/hypo-T4 (yellow) regions are shown.

B The TSH-T4 relation with TSH and T4 fixed points for Hashimoto's thyroiditis and Graves' disease (blue, red, respectively, same as in Fig 4) and in iodine deficiency conditions, determined for a range of b_{TH} values (Materials and Methods).

© 2022 The Authors
