# Supplementary material for: Dynamics of thyroid diseases and thyroid‐axis gland masses
Source: Mol Syst Biol. 2022 Aug 8;18(8):e10919. doi: 10.15252/msb.202210919 (PMC9358402; doi:10.15252/msb.202210919)
Supplement: Supplementary file 3 [file MSB-18-e10919-s002.pdf]

molecular systems biology

Dynamics of thyroid diseases and thyroid-axis gland masses

Yael Korem Kohanim, Tomer Milo, Moryia Raz, Omer Karin, Alon Bar, Avi Mayo, Netta Mendelson Cohen, Yoel Toledano, Uri Alon

DOI: 10.15252/msb.202210919

Corresponding author: Uri Alon (uri.alon@weizmann.ac.il)

Review Timeline:

Submission Date:	18th Jan 22
Editorial Decision:	21st Feb 22
Revision Received:	17th May 22
Editorial Decision:	17th Jun 22
Revision Received:	30th Jun 22
Accepted:	30th Jun 22

Editor: Jingyi Hou

Transaction Report:

(Note: With the exception of the correction of typographical or spelling errors that could be a source of ambiguity, letters and reports are not edited. Depending on transfer agreements, referee reports obtained elsewhere may or may not be included in this compilation. Referee reports are anonymous unless the Referee chooses to sign their reports.)

21st Feb 2022

Manuscript Number: MSB-2022-10919
Title: Dynamics of thyroid diseases and thyroid-axis gland masses
Author: Uri Alon
Yael Korem Kohanim
Tomer Milo
Moryia Raz
Omer Karin
Alon Bar
Avi Mayo
Netta Mendelson Cohen
Yoel Toledano

Thank you for submitting your work to Molecular Systems Biology. We have now heard back from the three reviewers who agreed to evaluate your manuscript. As you will see from the reports below, the reviewers acknowledge the potential interest of the study. They raise, however, a series of concerns, which we would ask you to address in a major revision.

Without reiterating all the points listed below, some of the more fundamental issues are the following:

1. Reviewer #1 raised concerns about data interpretation and several assumptions used in the model, which need to be addressed and/or discussed.

2. Reviewer #3 was mainly concerned about data presentation (especially the mathematical modeling part), which need to be addressed to improve the clarity of the study.

All other issues raised by the reviewers need to be satisfactorily addressed as well. As you may already know, our editorial policy allows in principle a single round of major revision, and it is therefore essential to provide responses to the reviewers' comments that are as complete as possible.

On a more editorial level, we would ask you to address the following issues:

- Please provide a .docx formatted version of the manuscript text (including legends for main figures, EV figures and tables). Please make sure that the changes are highlighted to be clearly visible.

- Please provide individual production quality figure files as .eps, .tif, .jpg (one file per figure).

-Please provide a .docx formatted letter INCLUDING the reviewers' reports and your detailed point-by-point responses to their comments. As part of the EMBO Press transparent editorial process, the point-by-point response is part of the Review Process File (RPF), which will be published alongside your paper.

-Please note that all corresponding authors are required to supply an ORCID ID for their name upon submission of a revised manuscript.

-We replaced Supplementary Information with Expanded View (EV) Figures and Tables that are collapsible/expandable online (see examples in http://msb.embopress.org/content/11/6/812). A maximum of 5 EV Figures can be typeset. EV Figures should be cited as 'Figure EV1, Figure EV2" etc... in the text and their respective legends should be included in the main text after the legends of regular figures.

Additional Tables/Datasets should be labeled and referred to as Table EV1, Dataset EV1, etc. Legends have to be provided in a separate tab in case of .xls files. Alternatively, the legend can be supplied as a separate text file (README) and zipped together with the Table/Dataset file.

For the figures and tables that you do NOT wish to display as Expanded View figures, they should be bundled together with their legends in a single PDF file called *Appendix*, which should start with a short Table of Content. Each legend should be below the corresponding Figure/Table in the Appendix. Appendix figures and tables should be referred to in the main text as: "Appendix Figure S1, Appendix Figure S2, Appendix Table S1" etc. See detailed instructions regarding expanded view here: https://www.embopress.org/page/journal/17444292/authorguide#expandedview.

-Before submitting your revision, primary datasets (and computer code, where appropriate) produced in this study need to be deposited in an appropriate public database (see https://www.embopress.org/page/journal/17444292/authorguide#dataavailability).

Please remember to provide a reviewer password if the datasets are not yet public.

The accession numbers and database should be listed in a formal "Data Availability" section (placed after Materials & Method) that follows the model below (see also https://www.embopress.org/page/journal/17444292/authorguide#dataavailability). Please note that the Data Availability Section is restricted to new primary data that are part of this study.

Data availability

The datasets (and computer code) produced in this study are available in the following databases:

- RNA-Seq data: Gene Expression Omnibus GSE46843 (https://www.ncbi.nlm.nih.gov/geo/query/acc.cgi?acc=GSE46843)
- [data type]: [name of the resource] [accession number/identifier/doi] ([URL or identifiers.org/DATABASE:ACCESSION])

*** Note - All links should resolve to a page where the data can be accessed. ***

- We would encourage you to include the source data for figure panels that show essential quantitative information. Additional information on source data and instruction on how to label the files are available at <
https://www.embopress.org/page/journal/17444292/authorguide#sourcedata
>.

- Our journal encourages inclusion of *data citations in the reference list* to directly cite datasets that were re-used and obtained from public databases. Data citations in the article text are distinct from normal bibliographical citations and should directly link to the database records from which the data can be accessed. In the main text, data citations are formatted as follows: "Data ref: Smith et al, 2001". In the Reference list, data citations must be labeled with "[DATASET]". A data reference must provide the database name, accession number/identifiers and a resolvable link to the landing page from which the data can be accessed at the end of the reference. Further instructions are available at .

- We updated our journal's competing interests policy in January 2022 and request authors to consider both actual and perceived competing interests. Please review the policy https://www.embopress.org/competing-interests and update your competing interests if necessary.

Please use the heading "Disclosure statement and competing interests".

- All Materials and Methods need to be described in the main text. We would encourage you to use 'Structured Methods', our new Materials and Methods format. According to this format, the Material and Methods section should include a Reagents and Tools Table (listing key reagents, experimental models, software and relevant equipment and including their sources and relevant identifiers) followed by a Methods and Protocols section in which we encourage the authors to describe their methods using a step-by-step protocol format with bullet points, to facilitate the adoption of the methodologies across labs. More information on how to adhere to this format as well as downloadable templates (.doc or .xls) for the Reagents and Tools Table can be found in our author guidelines: < https://www.embopress.org/page/journal/17444292/authorguide#researcharticleguide>. An example of a Method paper with Structured Methods can be found here: .

-Regarding data quantification:
Please ensure to specify the name of the statistical test used to generate error bars and P values, the number (n) of independent experiments (please specify technical or biological replicates) underlying each data point and the test used to calculate p-values in each figure legend. Discussion of statistical methodology can be reported in the materials and methods section, but figure legends should contain a basic description of n, P and the test applied.
Graphs must include a description of the bars and the error bars (s.d., s.e.m.).
Please also include scale bars in all microscopy images.

- Please provide a "standfirst text" summarizing the study in one or two sentences (approximately 250 characters, including space), three to four "bullet points" highlighting the main findings and a "synopsis image" (550px width and max 400px height, PNG format) to highlight the paper on our homepage.
Here are a couple of examples:
https://www.embopress.org/doi/10.15252/msb.20199356
https://www.embopress.org/doi/10.15252/msb.20209475
https://www.embopress.org/doi/10.15252/msb.209495

When you resubmit your manuscript, please download our CHECKLIST (http://bit.ly/EMBOPressAuthorChecklist) and include the completed form in your submission.

Please note that the Author Checklist will be published alongside the paper as part of the transparent process (https://www.embopress.org/page/journal/17444292/authorguide#transparentprocess).

If you feel you can satisfactorily deal with these points and those listed by the referees, you may wish to submit a revised version of your manuscript. Please attach a covering letter giving details of the way in which you have handled each of the points raised by the referees. A revised manuscript will be once again subject to review and you probably understand that we can give you no guarantee at this stage that the eventual outcome will be favorable.

I look forward to receiving your revised manuscript soon.

Sincerely,
Jingyi

Jingyi Hou
Editor
Molecular Systems Biology

Reviewer #1:

Summary
Korem's manuscript describes a new model to analyse and predict thyroid recovery upon treatment of either hypothyroidism or hyperthyroidism. The novelty of the model is to take into consideration changes in cell mass and their limits ("carrying capacities") at both pituitary (TSH-secreting thyrotrophs) and thyroid levels in order to model how TSH lags behind thyroid hormones (freeT4) upon treatment.
General remarks
The most convincing conclusion of the paper deals with the role of thyrotroph mass plasticity in modelling the TSH-TH setpoint in diseases since these cell mass changes, although reported in both human and animal models upon disease, were not considered as key units of TSH-TH setpoints in other models from Dietrich and Leow. Although the origin and exact biological role of these changes in cell mass are still matter of interpretation (see major points below), the concept raised in the model is highly relevant for both clinicians, modellers and basic researchers for better understanding of thyroid endocrinopathies which affect about 5% of the population worldwide.
Major points
Although I thank the authors for taking into account in their model the impressive changes in thyrotroph mass which have been reported from decades in distinct thyroid diseases, the authors should consider other published reports which would lead them to revise their interpretation of non-linear TSH-TH relationships as exemplified in the Hashimoto's hypothyroidism:

- From the authors' proposal, the initial sign of thyrotroph responsiveness in response to fT4 decrease would be the fast enhancement of TSH secretion. From what I understand in Fig3, this would be due to acute involvement of hypothalamic TRH release in the pituitary portal vessels. What are the experimental data supporting this proposal? There are conflicting data about the role of TRH inputs in TSH pulsatility such as the continuous TSH pulsatility in human beings receiving a sustained TRH delivery for 48 hours (Samuels and Ridgway doi:10.1089/thy.1993.3.201), the weak/moderate consequences of TRH/TRH-R1 gene deletion in animal models (PNAS 94, pp. 10862-10867, 1997; doi: 10.1210/me.2004-0017), the contribution of TRH inputs in TSH secretion has been considered as stochastic nature in former HPT models by Dietrich et al (doi: 10.3389/fendo.2015.00177 , Fig 3)
- The authors propose that increase in thyrotroph mass is delayed after the initial increase in TSH secretion. Is there any experimental data to support this?
- The key assumption of the model is that hypothyroidism induction causes an inhibition of TH-driven inhibition on thyrotroph cell proliferation. There is also an assumption that "secretion of TSH is assumed to be proportional to the total mass of the pituitary thyrotroph cells P, at a given level of TRH and TH". It is important to note that there are multiple mechanisms that could lead to an increase in cell number. Seminal studies from Levy's lab showed that 3 week thyroidectomy in rats triggered the expected increase in the number of TSH+ve thyrotrophs despite mitotic and apoptotic activity remaining unchanged (Journal of Endocrinology (2004) 180, 35-43). Would the authors also consider other mechanisms about changes in thyrotroph cell mass like transdifferentiation from other cell types to thyrotrophs (Lab Invest. 1990;63(4):511-520; J Cell Mol Med. 2003;7(3):297-306) and/or redifferentiation from dedifferentiated thyrotrophs (http://dx.doi.org/10.1016/j.cmet.2014.03.010)? The existence of different sub-sets of thyrotrophs was shown years ago in ultra-structural studies (Okajimas I-olia Anat. Jpn., 67(2-3): 175 12, August, 1990) and more recently identified in single-cell RNA-Seq data from human pituitary cells (doi: 10.1038/s41467-020-19012-4).
- In addition to a change in cell number, the functional mass of a cell type can be altered by increased hormone gene expression or release of stored hormone in response to stimulus. The Levy paper cited above, for example, described increases in TSHb mRNA that occur over a period of 3-5 days that are far higher than could be accounted for by an increased number of thyrotrophs. Whist there may be differences in axis regulation between humans and rodents (in particular the half-life of thyroid hormone), these other changes resulting in functional mass should be considered. These do not suggest the model is incorrect, as long as these changes, in addition to that of cell proliferation implied throughout the paper, are slow.
- The paucity of data in humans other than the size of the pituitary as disease progresses restricts testing of the model. In rodent models the changes occurring in particular in hypothyroidism are much more clearly defined- it would be valuable for the authors to test their model in these species using published data.
- The authors proposed that the switch from sub-clinical to overt hypothyroidism is due to both thyroid and pituitary mass that approach their carrying capacities. To my knowledge, this increase in pituitary mass upon overt hypothyroidism is smaller than the massive (x2-fold and more) increase in pituitary size upon lactation that is physiologically relevant and fully reversible after weaning. An alternative mechanism is the requirement for a low level of thyroid hormone for cell proliferation. As the level of thyroid hormone approaches zero, there will be a loss of proliferative response of thyrotrophs.

Reviewer #2:

In their manuscript titled "Dynamics of thyroid diseases and thyroid-axis gland masses" Yael Korem Kohanim and co-authors describe a new theory of thyroid hormone homeostasis that includes long-term effects of pathological conditions on the

respective functional masses of the pituitary and the thyroid gland. The model extends the theory of dynamic compensation that had been introduced by Karin et al. Parameters for the used equations were derived from a large dataset (Clalit database for medical records). With their new approach, the authors can explain a plethora of poorly understood phenomena in thyroid endocrinology, including hysteresis, the three-regime relationship between free T4 and TSH concentrations and the high prevalence of subclinical thyroid disorders.

This is a well-written manuscript that provides new insights and multiple long-awaited explanations for various phenomena. A few minor issues should be addressed, however, before acceptance can be considered.

In Figure 1, it is unclear why a delay (with consecutive hysteresis) results from treatment (i.e. after the black dashed line in panels 1A and 1B) but not from the onset of disease (origin of the curves in the left). If hysteresis were a universal phenomenon, one would expect that TSH rises with delay in ensuing hypothyroidism as well (panel 1A) and that it decreases in early Graves' disease with a delay as well. Are the time scales of the figure's two parts different?

The legend of figure 2 should explain the difference between panels E and G and between F and H, respectively.

A recent study found both pituitary and thyroid volume to correlate positively to gestational age in preterm and term infants but to correlate negatively to chronological age [Sayo Otani et al. 2021, PMID 33067897]. The authors should comment on this finding. Could it reflect a changing set point of thyroid homeostasis during and after fetal life? Of note, concentrations of TSH, T3 and T4 are low and rise slowly during the fetal period and peak at birth [see e.g. Morreale de Escobar et al. 1987, PMID 3297961 and Burrow et al. 1994, PMID 8090169].

Several typos should be fixed (e.g. "thyrotorpe" should be replaced in multiple locations by "thyrotrope").

It would be easier to review this manuscript if it contained line numbers. Therefore, continuous line numbering should be added in order to facilitate subsequent reviewing rounds.

Reviewer #3:

Overview

This paper proposes a refined mathematical model that captures certain features in the dynamics of thyroid diseases. In particular, an existing three dimensional model is augmented with two additional dimensions to incorporate the dynamics of the gland masses. The augmented dynamics evolve on a slower time scale and act as additional feedback control regulating the hormone levels. The slow time scale appended to the original model gave the possibility of capturing the hysteresis phenomenon that is observed in medical records. It also suggested that one biologically possible source of the observed hysteresis is the mass evolution of the glands. The basic idea of the paper is useful in advancing the mathematical modeling efforts in thyroid diseases. The paper is mostly well written except in the sections where mathematical arguments were brought up. In principle, I believe that the paper should be acceptable for publication at MSB after improving the mathematical presentation.
Here are some comments and suggestions:

Major Points

• The section "Delays and hysteresis in thyroid disorders" is a little vague. The intuitive explanation is clear; however, the math (in combination with the relevant Methods section) is unclear. In particular, the statement: "The ratio between the thyrotroph masses is predicted to equal the ratio between the TSH levels on the two trajectories at a given T4 level" does not appear to be supported by any type of analysis. Mathematically, this seems to be a strong conclusion that should be supported by analytical reasoning or at least extensive simulations if analytical derivations are not possible. Furthermore, the main message of the ratio equality is not delivered properly. I got the impression that this is useful to locate a patient on the hysteresis curve, but the idea is not conveyed properly and should be explained with more elaborations. Also, the math carried out in the Methods Section "Formula for functional thyrotroph mass" is not clear. How is the time derivative approximated? There should be some sort of 'Delta t' in the approximation. What is the role of the functional mass? What is P^? Why is it useful? More explanations and elaborations are required here.

• The authors exploit time-scale separation to invoke a quasi-steady state approximation which reduces the dynamics to two dimensions governed by the slow variables P and T. The steady-state analysis of the obtained two dimensional dynamical system is carried out by examining the nullclines. Since this paper is essentially a modeling paper, these mathematical steps should be clearly shown somewhere (as supplementary material for example). In particular, the equations of the nullclines are useful to look at

so that one can immediately see the e ects of a_T and Ab on the nullclines.

• It is particularly interesting that the appended dynamics are basically two integral feedback controllers realized (when the carrying capacities are infinite). This was touched upon very briefly in the discussion section. I believe that this should be emphasized more in the paper. This is especially the case, when the authors show simulations exhibiting adaptation without mentioning anything about integral control. It would be nice to include in the figure a cartoon explaining that the appended dynamics are non-ideal integral controllers where the non-ideal aspect comes naturally from the finite carrying capacity.

• It is important to emphasize the use of the proposed model: explain how "locating a patient on the hysteresis curve" can indeed help in the treatment procedure.

• Suggestion: It would be nice to see a figure where the data and the model results are overlaid. Perhaps some model fitting can be useful here.

• Overall, the paper requires some rewriting whenever mathematical arguments are brought up. The detailed derivations should be shown, if not in the main text or methods section, perhaps as supplementary material. It is unfortunate that the authors didn't use a latex-based environment for equations which made them cumbersome to read. The work flow for example can be rewritten as: (1) Write ODEs, (2) show adaptation in ideal cases (KT = KP = infinity), (3) clearly present the assumptions used to make the math easier, (4) divide the fast and slow dynamics and apply quasi-steady state approximation (of course one should prove that the slow manifold is stable to be rigorous), (4) write down the reduced model, (5) write down the nullclines in two dimensions showing the perturbed parameters (aT and Ab), (6) state the new assumptions, compute fixed points, prove stability to explicitly show the bifurcation diagram. It is easy to do all of these rigorously and write them down, and not resort to hand-wavy arguments that leave room for ambiguity and vagueness.

Minor Points

• Why aren't the data falling in the normal ranges not shown in Figure 1(G)? Also in the main text, no comment is given on that range and how it compares conceptually to Figure 1(D). The data in Figure 1(G) give the impression that the variation is steeper within the normal range (gray box). Is this correct?

• The causality of these statements: "The one-dimensionality of the problem provides the relation between TSH and T4. This relation has a steep slope at low T4 and high TSH values (Fig 4C, blue line)." is not clear. It is true that as aT (a single parameter) is varied, the fixed point slides on a one-dimensional manifold given by one of the two nullclines that is independent of aT . What does this have to do with providing the relation between TSH and T4? Also, how was the conclusion drawn regarding the slope at low and high TSH values? Statements like these should be proved.

• When the authors presented equation (2), they used the approximation TH >> K to get the inverse function model for inhibition. I don't understand why do that if most of the analysis presented is simulation-based and not analytical. This approximation is also inconsistent with the Michaelis-Menten terms used the Methods Section "Formula for functional thyrotroph mass". If approximations are not needed, it is better not to invoke them.

Dear Editor,
Thank you for your positive consideration and for the reviewer comments. We have now addressed all of the reviewer comments in the revised manuscript. We added a detailed SI with complete mathematical derivations, we added discussion and evidence for our assumptions and interpretations, including models for trans-differentiation, fetal gland mass changes and post-thyrodectomy dynamics, new comparisons to TSH and T4 measurements, and clarified the text throughout. The revised manuscript is more rigorous, comprehensive and clear.
We detail our point-by-point revisions below.
Sincerely,
Uri Alon

Reviewer #1:

Summary
Korem's manuscript describes a new model to analyse and predict thyroid recovery upon treatment of either hypothyroidism or hyperthyroidism. The novelty of the model is to take into consideration changes in cell mass and their limits ("carrying capacities") at both pituitary (TSH-secreting thyrotrophs) and thyroid levels in order to model how TSH lags behind thyroid hormones (freeT4) upon treatment.

General remarks
The most convincing conclusion of the paper deals with the role of thyrotroph mass plasticity in modelling the TSH-TH setpoint in diseases since these cell mass changes, although reported in both human and animal models upon disease, were not considered as key units of TSH-TH setpoints in other models from Dietrich and Leow. Although the origin and exact biological role of these changes in cell mass are still matter of interpretation (see major points below), the concept raised in the model is highly relevant for both clinicians, modellers and basic researchers for better understanding of thyroid endocrinopathies which affect about 5% of the population worldwide.

We thank the reviewer for this endorsement.

Major points
Although I thank the authors for taking into account in their model the impressive changes in thyrotroph mass which have been reported from decades in distinct thyroid diseases, the authors should consider other published reports which would lead them to revise their interpretation of non-linear TSH-TH relationships as exemplified in the Hashimoto's hypothyroidism:
- From the authors' proposal, the initial sign of thyrotroph responsiveness in response to fT4 decrease would be the fast enhancement of TSH secretion. From what I understand in Fig3, this would be due to acute involvement of hypothalamic TRH release in the pituitary portal vessels. What are the experimental data supporting this proposal? There are conflicting data about the

role of TRH inputs in TSH pulsatility such as the continuous TSH pulsatility in human beings receiving a sustained TRH delivery for 48 hours (Samuels and Ridgway doi:10.1089/thy.1993.3.201), the weak/moderate consequences of TRH/TRH-R1 gene deletion in animal models (PNAS 94, pp. 10862-10867, 1997; doi: 10.1210/me.2004-0017), the contribution of TRH inputs in TSH secretion has been considered as stochastic nature in former HPT models by Dietrich et al (doi: 10.3389/fendo.2015.00177 , Fig 3)

We thank the reviewer for this comment, which helped us clarify the mechanism in the main text and add the cited references for TRH effects. Indeed, FT4 decrease is assumed to enhance TSH secretion. However, we now clarify that this is mainly due to the release of TSH suppression by FT4 action on the pituitary- regardless of the involvement of TRH in the process.
In the model, we consider two main effects on TSH secretion: 1. TRH drives TSH secretion, and 2. Thyroid hormones (TH) T4 and T3 suppress both TRH secretion and TSH secretion of the pituitary. The main effect for the phenomena in this paper is the direct effect on the pituitary.
To clarify this point, we now added the following new sentences to the revised results section "Dynamic compensation of physiological changes":

> *"The rise of TSH in the model is due primarily to the release of inhibition of thyroid hormones on TSH production in the pituitary. A secondary effect, which is not essential for our conclusions, is due to release of TRH inhibition. We note that the role of TRH in this context is debated in the literature (Hoermann et al., 2015a; Rabeler et al., 2004; Samuels et al., 1993)."*

- The authors propose that increase in thyrotroph mass is delayed after the initial increase in TSH secretion. Is there any experimental data to support this?

We thank the reviewer for this comment which helped us seek experimental evidence for TSH rise and increase in thyrotroph mass after abrupt change in TH levels. We found experimental evidence that TSH levels start to rise a few days after thyroidectomy in humans (e.g. Kim et al., 2005 DOI 10.3803/jkes.2005.20.5.460). Thyrotroph mass was shown to rise three weeks after thyroidectomy in mice (Nolan et al., 2004 DOI 10.1677/joe.0.1800035), and this might be equivalent to a longer period in humans, due to the longer half-life times of the hormones. The difference in time scales make sense due to the much slower timescale of cell growth compared to hormone secretion changes. However, we didn't find measurements of thyrotroph mass at earlier times after thyroidectomy. We suggest this experiment in the revised discussion:

> *"It would be important to measure thyrotroph and thyrocyte masses as well as TSH levels as a function of time after thyroid perturbations in humans and rodents (Nolan et al., 2004) in order to test the model predictions, such as delays in thyrotroph mass increase after an initial TSH rise."*

- The key assumption of the model is that hypothyroidism induction causes an inhibition of TH-driven inhibition on thyrotroph cell proliferation. There is also an assumption that "secretion of TSH is assumed to be proportional to the total mass of the pituitary thyrotroph cells P, at a given level of TRH and TH". It is important to note that there are multiple mechanisms that could lead to an increase in cell number. Seminal studies from Levy's lab showed that 3 week thyroidectomy in rats triggered the expected increase in the number of TSH+ve thyrotrophs despite mitotic and apoptotic activity remaining unchanged (Journal of Endocrinology (2004) 180, 35-43). Would the authors also consider other mechanisms about changes in thyrotroph cell mass like transdifferentiation from other cell types to thyrotrophs (Lab Invest. 1990;63(4):511-520; J Cell Mol Med. 2003;7(3):297-306) and/or redifferentiation from dedifferentiated thyrotrophs (http://dx.doi.org/10.1016/j.cmet.2014.03.010)? The existence of different sub-sets of thyrotrophs was shown years ago in ultra-structural studies (Okajimas I-olia Anat. Jpn., 67(2-3): 175 12, August, 1990) and more recently identified in single-cell RNA-Seq data from human pituitary cells (doi: 10.1038/s41467-020-19012-4).

We thank the reviewer for this important comment, which helped us to add a new model with transdifferention, to make the text and SI more rigorous and to add these key references. We now write in the main text:

> *"The growth of thyrotroph cells can occur by additional means (Nolan et al., 2004) such as trans-differentiation from other pituitary cells (Horvath et al., 1990; Radian et al., 2003), progenitor cells or de-differentiated subpopulations (Wang et al., 2014)* (Appendix Figure S2*, See analysis of these cases in Appendix Supplementary Text)."*

In the new SI section "Trans-differentiation from other cell types into thyrotrophs", we explore the ramification of thyrotroph mass changes from non-thyrotroph cells. This adds to the equation a source term inhibited by TH. We find that the trans-differentiation model has qualitatively similar dynamics to the original model in the healthy and the hypothyroid regimes. The models differ in their predictions for severe hyperthyroidism such as in extreme cases of Graves' disease. In this regime, the original model predicts that the thyrotroph mass shrinks to zero, causing TSH levels to drop to very low levels. In the trans-differentiation model this effect is less drastic, with a gradual decrease in thyrotroph mass and TSH when thyroid hormone levels rise.

The new SI section reads as follows:

Trans-differentiation from other cell types into thyrotrophs

In this section, we consider other mechanisms for thyrotroph mass accumulation other than thyrotroph proliferation or growth. This includes trans-differentiation from other pituitary cell types (Horvath et al., 1990; Radian et al., 2003) and/or redifferentiation from dedifferentiated thyrotrophs (Wang et al., 2014).
Mathematically, we account for these effects by adding to the equation that describes the pituitary thyrotroph mass dynamics a source term inhibited by thyroid hormone levels.

$$(1)\ \frac{dP(t)}{dt} = \frac{b_{Pcross}}{x3(t)}(1 - k_P\,P(t)) + P(t)(\frac{b_P}{x_3(t)}(1 - k_P\,P(t)) - a_P)$$

The other model equations (1)-(4) remain the same.
We find that adding this term leaves the results of this analysis qualitatively similar.
As before, we rescale the variables by their steady-state value in the simple case in which the glands are far from their carrying capacities (i.e. infinite carrying capacity kT=kP=0), there are no TSHR antibodies and no external thyroid hormone supply (Ab=0, b30=0), and x3 depends approximately linearly on x2 (kx2=0). However here we keep the parameter that describes the thyrotroph proliferation/growth rate bp *in the equations, so latter it can be compared to* bp_{cross}*, the rate of thyrotroph mass accumulation from non-thyrotroph cells:*

$$\{x_1 \to \frac{a_P\,b_1\,u}{a_1}, x_2 \to \frac{a_T}{b_T}x_2, x_3 \to \frac{1}{a_P}x_3, P \to \frac{a_1\,a_2\,a_T}{a_P{}^2\,b_1\,b_2\,b_T\,u}P, T \to \frac{a_3\,b_T}{b_3\,a_P\,a_T}T\}$$

Rescaling the variables, we define new variables:

$$Ab = \frac{a_T}{b_T}AB, \qquad k_{x2} = \frac{b_T}{a_T}K_{X2}, \qquad k_T = \frac{a_P\,a_T\,b_3}{b_T\,a_3}K_T,$$

$$kP = \frac{ap^2\,b1\,b2\,bt}{a1\,a2\,at}KP, \qquad b30 = \frac{a3}{ap}B30, \qquad bpcross \to \frac{a1\,a2\,at\,BPcross}{ap^2\,b1\,b2\,bt\,u}$$

The new scaled equations are:

$$(2)\ \frac{1}{a_1}x_1'(t) = \frac{1}{x_3(t)} - x_1(t)$$

$$(3)\ \frac{1}{a_2}x_2'(t) = P(t)\frac{x_1(t)}{x_3(t)} - x_2(t)$$

$$(4)\ \frac{1}{a_3}x_3'(t) = B_{30} + \left(T(t)\frac{AB+x_2(t)}{1+K_{X2}(AB+x_2(t))} - x_3(t)\right)$$

$$(5)\ \frac{1}{a_T}T'(t) = T(t)\left(\frac{AB+x_2(t)}{1+K_{X2}(AB+x_2(t))}(1 - K_T\,T(t)) - 1\right)$$

$$(6)\ \frac{1}{a_P}P'(t) = \frac{B_{Pcross}+b_p\,P(t)}{x_3(t)}\left(1 - K_P\,P(t)\right) - P(t)$$

Computing the steady-state for this system in the simple case described above, we find that the steady-state of x3 (thyroid hormone) is now dependent on the thyrotroph mass growth rate b_P*, and on the trans-differentiation term* bp_{cross} *(Figure S1A) . However, if the sum of rates* $bp + b_{Pcross}$ *remains constant, x3 does not change. In other words, the overall rate of thyrotroph mass accumulation matters, but not their specific source.*
When P approaches its carrying capacity, i.e. $K_P > 0$*, the symmetry between the trans-differentiation model to the original model starts to break (Figure S1B). In this case, replacing thyrotroph proliferation bp with thyrotroph synthesis from non-thyrotroph source* b_{Pcross}*, has a similar effect on thyroid hormone levels but with scaling: Higher* b_{Pcross} *values map to lower bp values. Therefore, higher trans-differentiation rate is required to maintain a similar thyrotroph mass.*
Next, we tested the effect of adding a trans-differentiation term on the TSH(TH) relationship (Figure S1C). The models differ in their prediction for the TSH(TH) relation in hyperthyroidism. The original model predicts a sharp drop to very low TSH values even in subclinical hyperthyroidism with thyroid hormone levels that are only slightly above normal. However, the new model predicts a gradual drop in TSH with the rise in TH levels.
This difference stems from the behaviors of the two models in the regime of small thyrotroph mass. In the original model, when thyrotroph mass is equal to zero it cannot be restored again. Therefore, in Graves' disease, for high enough antibodies level, the model has a fixed point at zero thyrotroph mass: $\left\{x_1 = \frac{K_T}{AB-1}, x_2 = 0, x_3 = \frac{AB-1}{K_T}, P = 0, T = \frac{1}{K_T}(1 - \frac{1}{AB})\right\}$ *(See Appendix section "Dependence on antibody parameter in Graves' disease" and Figure EV4). In this*

regime, the thyrotroph mass and TSH levels cannot compensate for the antibody stimulation, leading to increased TH levels and enlarged of thyroid mass. However, in the trans-differentiation model, thyrotrophs mass is generated even when it is low, due to the non-thyrotroph sources. Therefore, the fixed point at P=0 is lost. More data is needed to conclusively differentiate between these predictions.

Nullcline analysis and stream plot simulations reveal that the full dynamics, and not only the steady-state, remain qualitatively similar (Fig S1D-F). The nullclines for the model when $K_{X2} = B_{30} = AB = 0$ *are:*

$$\{T^2 = P(1 - K_T\, T)^3 \quad OR \quad T = 0, \qquad P^2 = \frac{(B_{Pcross} + b_P P)^3 (1 - K_P\, P)^3}{T}\}$$

However, a major difference between the models is that the original model has a nullcline at P=0, while the new model doesn't. This leads to the difference described above, in the regime of high thyroid mass T and small thyrotroph mass P.

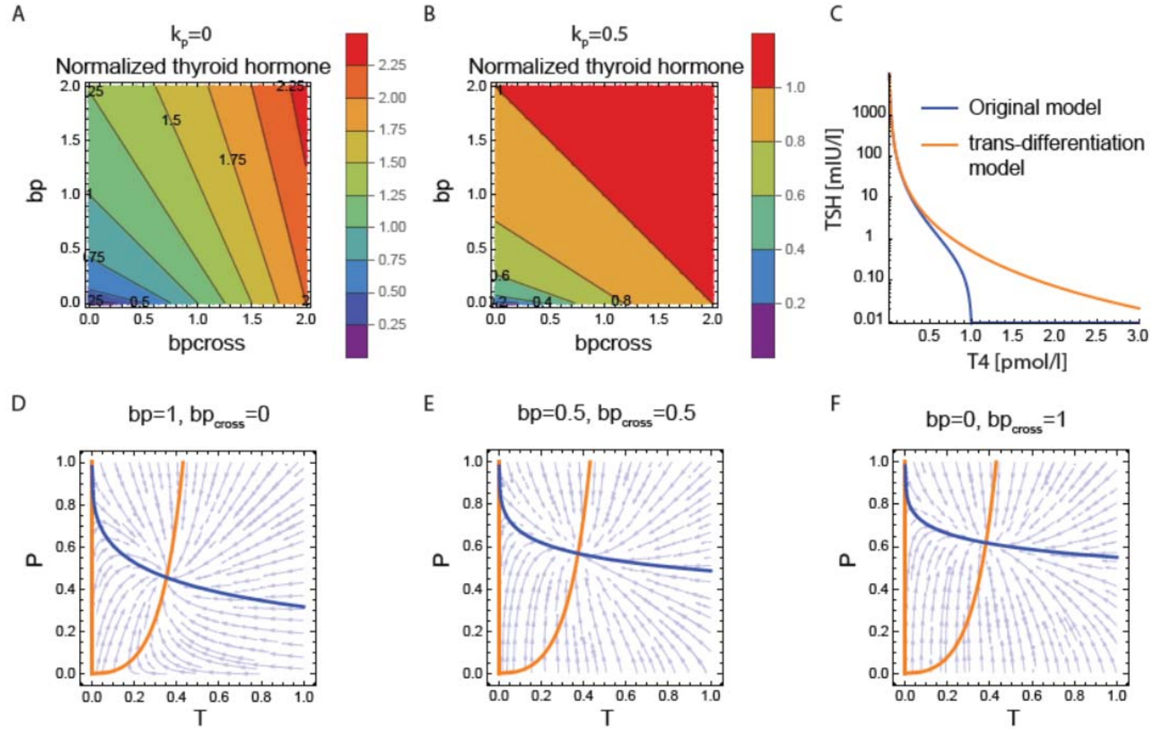

Figure S1: Model with trans-differentiation term has similar dynamics in the euthyroid and hypothyroid regimes but not in hyperthyroidism. (A,B) Steady-state thyroid hormone levels in a model with trans-differentiation to thyrotrophs from non-thyrotroph cell types for a range of thyrotroph proliferation/growth rates b_p and trans-differentiation rates $b_{p_{cross}}$. When $b_{p_{cross}} = 0$ the original model is restored. (A) P is far from its carrying capacity $k_P = 0$ (B) $k_P = 0.5$. (C) TSH(TH) relation in the trans-differentiation model compared to the original model. (D,E,F) Nullclines and stream plots for the trans-differentiation model for different b_p,$b_{p_{cross}}$ values.

- In addition to a change in cell number, the functional mass of a cell type can be altered by

increased hormone gene expression or release of stored hormone in response to stimulus. The Levy paper cited above, for example, described increases in TSHb mRNA that occur over a period of 3-5 days that are far higher than could be accounted for by an increased number of thyrotrophs. Whist there may be differences in axis regulation between humans and rodents (in particular the half-life of thyroid hormone), these other changes resulting in functional mass should be considered. These do not suggest the model is incorrect, as long as these changes, in addition to that of cell proliferation implied throughout the paper, are slow.

We believe that the increase over days in TSH mRNA that is higher than explainable by thyrotroph mass increase is captured by our model. The time of days comes from the half-life of T4 (7 days in human and probably 1-2 days in mouse). Thus, a drop in T4 production would result in a drop of T4 levels over days, increasing TSH mRNA expression by relieved suppression. We now added a new SI section to discuss this point: Appendix supplementary Text: "Rise of TSH mRNA over days following thyroidectomy"

"Rise of TSH mRNA over days following thyroidectomy
Nolan et al provide evidence of a rise in TSH mRNA over days after thyroidectomy (Nolan et al., 2004). The present model captures this effect, based on the lifetime of TH. This lifetime is seven days in humans and is thought to be on the order of a few days in mice. Thus, after thyroidectomy, thyroid hormone levels fall gradually, reaching halfway down after its half-life. In this timescale of days, TSH mRNA expression in the thyrotrophs will be gradually de-repressed, and their levels would rise (Fig EV2). In contrast, thyrotroph mass will grow on a longer time scale of about a month. Figure EV2 illustrates this point: After thyroidectomy at time 0, T4 drops and TSH increases, reaching their minimal/maximal concentrations respectively after about 7 days. However, thyrotroph mass P does not reach its maximal point even after 30 days."

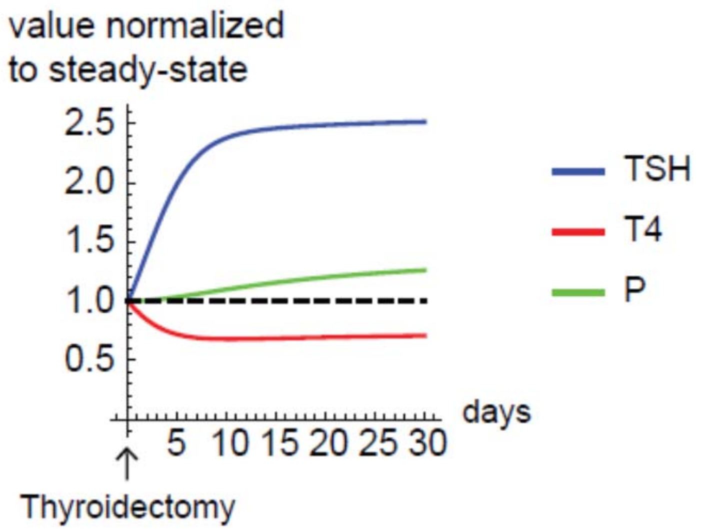

Figure EV2: Model dynamics after thyroidectomy. After thyroidectomy at time 0, T4 drops and TSH increases, reaching their minimal/maximal concentrations respectively after about 7 days. However, thyrotroph mass P does not reach its maximal value even after 30 days. All variables are normalized to their healthy steady-state value.

- The paucity of data in humans other than the size of the pituitary as disease progresses restricts testing of the model. In rodent models the changes occurring in particular in hypothyroidism are much more clearly defined- it would be valuable for the authors to test their model in these species using published data.

We agree and unfortunately could not find TSH measurements in mice. We now add this to the suggested experiments in the discussion:

> *"It would be important to measure thyrotroph and thyrocyte masses as well as TSH levels as a function of time after thyroid perturbations in humans and rodents (Nolan et al., 2004) in order to test the model predictions, such as delays in thyrotroph mass increase after an initial TSH rise."*

- The authors proposed that the switch from sub-clinical to overt hypothyroidism is due to both thyroid and pituitary mass that approach their carrying capacities. To my knowledge, this increase in pituitary mass upon overt hypothyroidism is smaller than the massive (x2-fold and more) increase in pituitary size upon lactation that is physiologically relevant and fully reversible after weaning. An alternative mechanism is the requirement for a low level of thyroid hormone for cell proliferation. As the level of thyroid hormone approaches zero, there will be a loss of proliferative response of thyrotrophs.

We now add this elegant alternative mechanism to the discussion:

"In Hashimoto's thyroiditis, the transition from subclinical to clinical disease occurs in the model when the autoimmune killing rate is so high that thyrotroph mass approaches its carrying capacity, and can no longer compensate by increased TSH secretion for the loss of thyroid function. Such a carrying capacity might be due to the confined anatomical position of the pituitary, together with the need for other pituitary cell types. Some experimental reports show a 5-fold increase in pituitary volume in hypothyroidism (Khawaja, 2006) while others show a smaller increase. The thyrotroph carrying capacity requires further study; it may not result in observable volume growth if carrying capacity is imposed by other cell types. Alternatively, a low level of thyroid hormones may be required for thyrotroph proliferation."

Reviewer #2:

In their manuscript titled "Dynamics of thyroid diseases and thyroid-axis gland masses" Yael Korem Kohanim and co-authors describe a new theory of thyroid hormone homeostasis that includes long-term effects of pathological conditions on the respective functional masses of the pituitary and the thyroid gland. The model extends the theory of dynamic compensation that had been introduced by Karin et al. Parameters for the used equations were derived from a large dataset (Clalit database for medical records). With their new approach, the authors can explain a plethora of poorly understood phenomena in thyroid endocrinology, including hysteresis, the three-regime relationship between free T4 and TSH concentrations and the high prevalence of subclinical thyroid disorders.

This is a well-written manuscript that provides new insights and multiple long-awaited explanations for various phenomena. A few minor issues should be addressed, however, before acceptance can be considered.

We thank the reviewer for this endorsement.

In Figure 1, it is unclear why a delay (with consecutive hysteresis) results from treatment (i.e. after the black dashed line in panels 1A and 1B) but not from the onset of disease (origin of the curves in the left). If hysteresis were a universal phenomenon, one would expect that TSH rises with delay in ensuing hypothyroidism as well (panel 1A) and that it decreases in early Graves' disease with a delay as well. Are the time scales of the figure's two parts different?

Thank you for this comment. The time scales of the two parts of Fig 1A (before and after the dashed lines) are indeed different. We now improved Fig 1A by adding "//" signs to indicate that the disease process is much longer than treatment. We also added to the caption:

"note that the disease develops on a much longer timescale than the treatment part of the

figure."

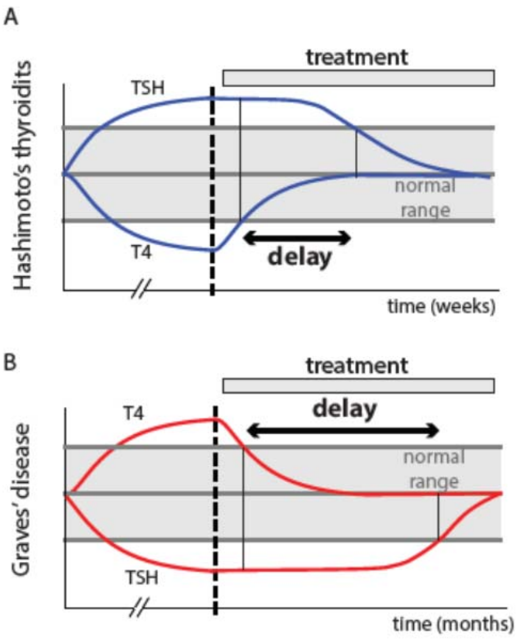

Regarding the delay during disease/treatment: In the disease process, T4 is gradually reduced, causing a gradual increase in TSH and a gradual corresponding increase in thyrotroph pituitary mass. In the treatment phase, T4 is quickly normalized due to the thyroid hormone replacement therapy. However, the pituitary mass takes more time to shrink back, leading to the sustained increased TSH levels – hence the delay in TSH normalization.

The legend of figure 2 should explain the difference between panels E and G and between F and H, respectively.

We now improved the legend of figure 2. In addition, we rearranged the figure panels and reformatted the axes labels to make the difference between panels E,G and F,H more clear:

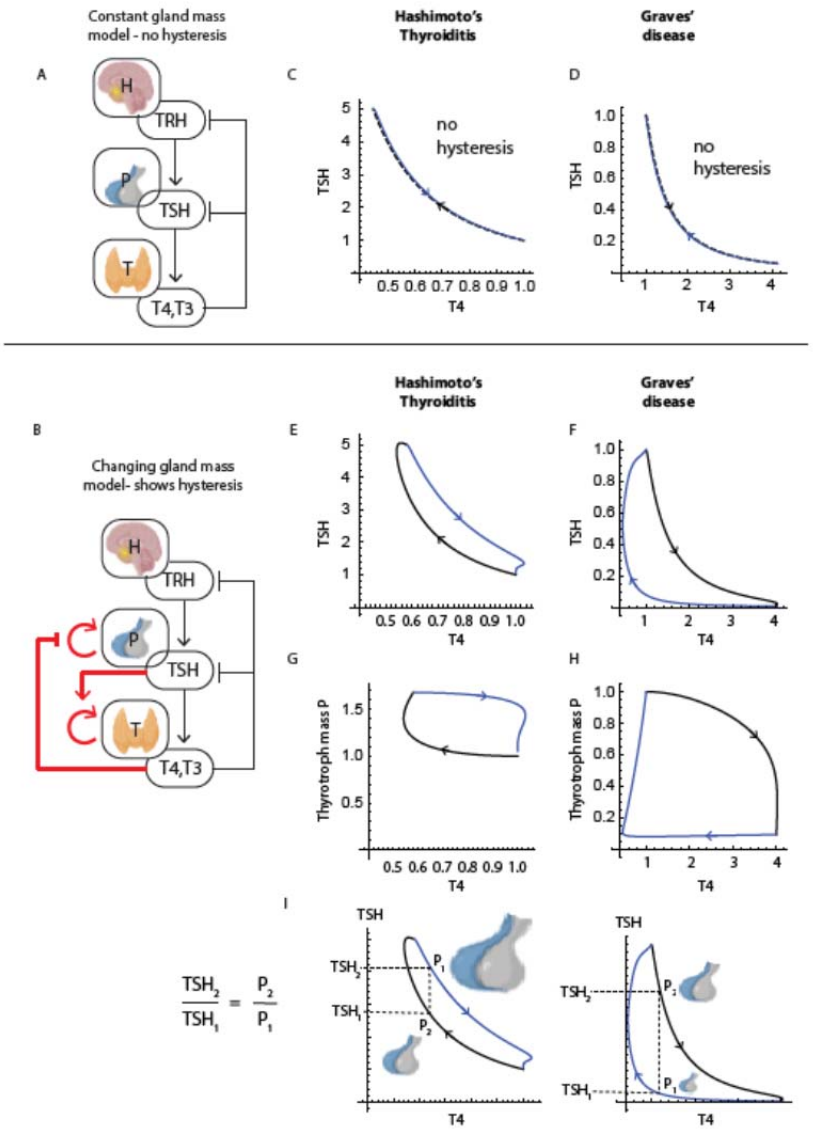

Figure 2: Gland-mass model explains hysteresis by a slow recovery of thyrotroph mass. (A) HPT model with constant gland mass (B) HPT model with gland mass changes, with new interactions in red. Thyrotroph and thyrocyte turnover times are on the order of a month. The constant-mass model does not show hysteresis in simulations of (C) Hashimoto's thyroiditis and (D) Graves' disease. TSH is uniquely determined by TH levels. *(E,F): TSH-T4 trajectories in a model with gland mass changes show TSH delay in Hashimoto's thyroiditis (E) and in Graves' disease (F). (G,H): TSH-thyrotroph mass P trajectories show that the delay is due to an enlarged thyrotroph mass that takes many weeks to shrink back to baseline after T4 is normalized in Hashimoto's thyroiditis (G), or due to atrophied thyrotroph mass that takes months to regrow after T4 is normalized by treatment in Graves' disease (H). (I) Model indicates that the ratio of thyrotroph masses at a given T4 level before and after treatment is equal to the ratio of TSH levels. Black lines represent trajectories during disease, from the normal set point to hypo/hyperthyroidism. Blue lines represent trajectories after diagnosis and during treatment back to the normal set point.*

A recent study found both pituitary and thyroid volume to correlate positively to gestational age in preterm and term infants but to correlate negatively to chronological age [Sayo Otani et al. 2021, PMID 33067897]. The authors should comment on this finding. Could it reflect a changing set point of thyroid homeostasis during and after fetal life? Of note, concentrations of TSH, T3 and T4 are low and rise slowly during the fetal period and peak at birth [see e.g. Morreale de Escobar et al. 1987, PMID 3297961 and Burrow et al. 1994, PMID 8090169].

Thank you for this suggestion. We now added a new SI section to discuss these findings, and mention them in the discussion:

> *"The model can explore changes during gestation. Interestingly, the finding of coordinated P and T volumes changes (Otani et al., 2021) sets constraints on which parameter group might change during gestation(Appendix supplementary text)."*

In the new Appendix section "Changes in fetal thyroid and pituitary volume during gestation" we write:

> ***"Changes in fetal thyroid and pituitary volume during gestation***
> *A recent study found both pituitary and thyroid volume to correlate positively to gestational age in preterm and term infants but to correlate negatively to chronological age (Otani et al., 2021). Changes in volume during gestation may result from many different physiological effects. If we assume that the main change is due to the thyroid axis modeled here, the coordinated changes in P and T offer interesting constraints on which parameter group changes during gestation.*
> *The thyroid and pituitary set points in the model, assuming they are not close to their carrying capacities, are:*
>
> $$T = \frac{a_{TH} b_P b_T}{b_{TH} a_P a_T}, P = \frac{a_{TRH} a_{TSH} a_T {b_P}^2}{b_{TRH} b_{TSH} b_T {a_P}^2 u}$$
>
> *The change in T,P set-point before and after birth suggested by Otani et al. can thus reflect change in parameters in utero and after birth. Interestingly, P and T are observed to change together: they grow together before birth, and shrink together after birth. This fact sets a constraint on which parameter is responsible for this transition, because this parameter should affect P and T in the same direction. Therefore, the model combined with the findings of Otani et al. suggests that the parameter responsible for the opposite trends in gland volume change before and after birth is either b_P and/or a_P: the pituitary thyrotroph production and removal rates. This would be the case if thyrotroph proliferation rate is higher in utero than after birth. Note that these pituitary parameters dictate the volume accumulation of both glands – pituitary and thyroid. Another interesting prediction is the pituitary volume is more sensitive to changes in bp and ap due to the quadratic dependence on b_P and a_P, compared to the linear dependency of T on these parameters. This is in line with Otani et al that showed larger relative volume changes for P than for T for the same time period."*

Several typos should be fixed (e.g. "thyrotorpe" should be replaced in multiple locations by "thyrotrope").

Done, we use thyrotroph throughout.

It would be easier to review this manuscript if it contained line numbers. Therefore, continuous line numbering should be added in order to facilitate subsequent reviewing rounds.

Done.

Reviewer #3:

Overview

This paper proposes a refined mathematical model that captures certain features in the dynamics of thyroid diseases. In particular, an existing three dimensional model is augmented with two additional dimensions to incorporate the dynamics of the gland masses. The augmented dynamics evolve on a slower time scale and act as additional feedback control regulating the hormone levels. The slow time scale appended to the original model gave the possibility of capturing the hysteresis phenomenon that is observed in medical records. It also suggested that one biologically possible source of the observed hysteresis is the mass evolution of the glands. The basic idea of the paper is useful in advancing the mathematical modeling efforts in thyroid diseases. The paper is mostly well written except in the sections where mathematical arguments were brought up. In principle, I believe that the paper should be acceptable for publication at MSB after improving the mathematical presentation. Here are some comments and suggestions:

We thank the reviewer for these comments, which helped us to improve the paper and make it more mathematically rigorous. We added a supplementary section with detailed derivation of the model, analytical computation of the fixed points and nullclines and fixed-point stability analysis. We also made the method sections more detailed and clear and added derivations where needed.

We furthermore derived analytical solutions for the TSH-T4 relationship and compared it directly to data.

Major Points

• The section "Delays and hysteresis in thyroid disorders" is a little vague. The intuitive explanation is clear; however, the math (in combination with the relevant Methods section) is unclear. In particular, the statement: "The ratio between the thyrotroph masses is predicted to equal the ratio between the TSH levels on the two trajectories at a given T4 level" does not appear to be supported by any type of analysis. Mathematically, this seems to be a strong conclusion that should be supported by analytical reasoning or at least extensive simulations if analytical derivations are not possible.

We now added a methods section with a mathematical proof of the statement as follows:

> *"**Ratio between thyrotroph masses on two hysteresis trajectories at a given T4 level is equal to the ratio between the TSH levels.***
> *Assume a hysteresis loop that is composed of two trajectories: (1) before diagnosis and (2) after diagnosis and during treatment. We define TSH levels at a given T4 level on these trajectories, TSH_1 and TSH_2 respectively (Fig 2I). In this section, we provide a mathematical proof that the ratio between these TSH values TSH_1/TSH_2, is equal to the thyrotroph mass ratio at these points P_1/P_2.*
> *From equation (3), for a constant environmental signal u, TRH is uniquely defined by TH:*
>
> $$TRH = \frac{b_{TRH} u}{a_{TRH}} \frac{1}{TH}$$
>
> *From equation (2), at steady-state:*
>
> $$TSH = \frac{b_{TSH}}{a_{TSH}} P \frac{TRH}{TH} = \frac{b_{TSH}}{a_{TSH}} P \frac{1}{TH^2}$$
>
> *Therefore for a constant thyroid hormone level TH_0:*
>
> $$TSH_1 = \frac{b_{TSH}}{a_{TSH}} P_1 \frac{1}{{TH_0}^2}, \quad TSH_2 = \frac{b_{TSH}}{a_{TSH}} P_2 \frac{1}{{TH_0}^2}$$
>
> *Hence:*
>
> $$\frac{TSH_1}{TSH_2} = \frac{P_1}{P_2}."$$

Furthermore, the main message of the ratio equality is not delivered properly. I got the impression that this is useful to locate a patient on the hysteresis curve, but the idea is not conveyed properly and should be explained with more elaborations.

We now address this in the following new sentence (in bold) in the result section "Delays and hysteresis in thyroid disorders:

> *"The essence of hysteresis in the model is thus the changes in pituitary thyrotroph mass*

P during the disease process that take many weeks to recover after treatment begins. ***Having an estimate for the thyrotroph mass during treatment of thyroid disease can thus help to locate a patient on the hysteresis curve in order to guide treatment strategies.*** *The model provides an estimate for relative changes in P based on the hysteresis trajectories. The ratio between the thyrotroph masses is predicted to equal the ratio between the TSH levels on the two trajectories at a given T4 level (Methods, Fig 2I):"*

Also, the math carried out in the Methods Section "Formula for functional thyrotroph mass" is not clear.

How is the time derivative approximated? There should be some sort of 'Delta t' in the approximation. What is the role of the functional mass? What is P^? Why is it useful? More explanations and elaborations are required here.

We thank the reviewer for this comment that helped us to clarify the proof and fix typos.

We revised the Methods section as follows:

"Formula for functional thyrotroph mass:

To derive a formula for the functional mass of the pituitary P based on a TRH test, we use equation (2), with Michaelis-Menten terms for TSH stimulation by TRH and for TSH suppression by T4:

$$dTSH/dt = b_{TSH} P \frac{TRH}{TRH + k_{TRH}} \frac{k_{T4}}{k_{T4} + T4} - a_{TSH} TSH$$

To estimate P requires three measurements (i) basal TSH levels TSH_{basal},(ii) TSH after TRH stimulation (usually after \Delta~30min) $TSH_{stimulated}$, and (iii) basal T4 levels $T4_{basal}$. Given that the TRH test uses a saturating amount of TRH, $TRH \gg k_{TRH}$, we approximate the temporal derivative using the difference divided by \Delatt

$$\frac{TSH_{stimulated} - TSH_{basal}}{\Delta t} \approx b_{TSH} P (1 + \frac{k_{T4}}{T4}) - a_{TSH} TSH_{basal}$$

We define ΔTSH, the rise in TSH following the TRH test when taking into account TSH turnover rate a_{TSH}, as:

$$\Delta TSH = TSH_{stimulated} - TSH_{basal}(1 + a_{TSH} \Delta t),$$

Therefore:

$$P = \frac{1}{b_{TSH} \Delta t} \Delta TSH (1 + \frac{T4}{k_{T4}})$$

Note that b_{TSH}, the maximal TSH secretion rate per thyrotroph per unit TRH and unit T4, is a characteristic of the system that we assume is constant, and Δt is a test parameter, the time for the TSH measurement after TRH stimulation, that can be calibrated to improve the accuracy of this formula for partical use.

Therefore, the formula $P\Delta TSH(1+\frac{T4}{k_{T4}})$ is an estimate for the thyrotroph functional mass (i.e. the secretory capacity of P), that can be used to estimate its change during treatment of thyroid diseases."

• The authors exploit time-scale separation to invoke a quasi-steady state approximation which reduces the dynamics to two dimensions governed by the slow variables P and T. The steady-state analysis of the obtained two dimensional dynamical system is carried out by examining the nullclines. Since this paper is essentially a modeling paper, these mathematical steps should be clearly shown somewhere (as supplementary material for example). In particular, the equations of the nullclines are useful to look at so that one can immediately see the e ects of a_T and Ab on the nullclines.

We thank the reviewer for this comment. We now added a detailed supplementary section with the analytical derivation of the nullclines (the full supplementary is appended to this letter below). We also added the nullcline equations to the revised Methods as follows:

"Computation of nullcline curves for the gland mass model

"To compute the nullclines in Fig 4A,B, and in Fig EV3, EV5 we used the separation of time scales in the model between the hormone turnover times and the slower gland turnover times. We thus assumed that the hormones are in steady-state, and computed the nullclines by setting $dT/dt = 0$ or $dP/dt = 0$. In the simple case where the response function of TH is linear $f(TSH) = TSH$, and that there is no external levothyroxine supply, this gives the following nullclines:

T'=0:

$$\{P = \frac{(T+B_{30}(1-K_T\,T))^2}{(1-K_T\,T)^2}\left(\frac{1}{1-K_T\,T-K_{X2}} - AB\right) \quad OR \quad T = 0\}$$

P'=0:

$$\{T = (1 - B_{30} - K_P P)\frac{\left(1+AB\,K_{X2}\left(1+\frac{P}{(1-K_P P)^2}\right)\right)}{AB+\frac{P}{(1-K_P P)^2}} \quad OR \quad P = 0\}$$

where $K_T = \frac{a_{TH}b_P b_T}{b_{TH}a_P a_T}k_T$, $K_P = \frac{a_{TRH}\,a_{TSH}\,a_T\,b_P^2}{b_{TRH}\,b_{TSH}\,b_T\,a_P^2}k_P$ and $AB = \frac{b_T}{a_T}Ab$.

In the simple case of $B_{30} = K_{X2} = AB = 0$, the nullclines in take the simpler form of:

$$\{\ P = \frac{T^2}{(1-K_T\,T)^3} \quad OR \quad T = 0, \qquad T = \frac{(1-K_P\,P)^3}{P} \quad OR \quad P = 0$$

For details see Appendix Supplementary Text. "

• It is particularly interesting that the appended dynamics are basically two integral feedback controllers realized (when the carrying capacities are infinite). This was touched upon very briefly in the discussion section. I believe that this should be emphasized more in the paper. This is especially the case, when the authors show simulations exhibiting adaptation without mentioning anything about integral control. It would be nice to include in the figure a cartoon explaining that the appended dynamics are non-ideal integral controllers where the non-ideal aspect comes naturally from the finite carrying capacity.

We now added to the text emphasis on the integral feedback structure, and a cartoon showing non-ideal integral controllers.

We write on pg 10:

> ***"****The adaptation of TSH and TH results from the integral feedback nature of Eq (4),(5). When the glands are far from their carrying capacities, the only possibility to get a steady state with nonzero gland sizes is for hormone-controlled proliferation to equal removal, and hence* $TSH = \frac{at}{bt}$ *,* $TH = \frac{bp}{ap}$ *(Fig EV1).****"***

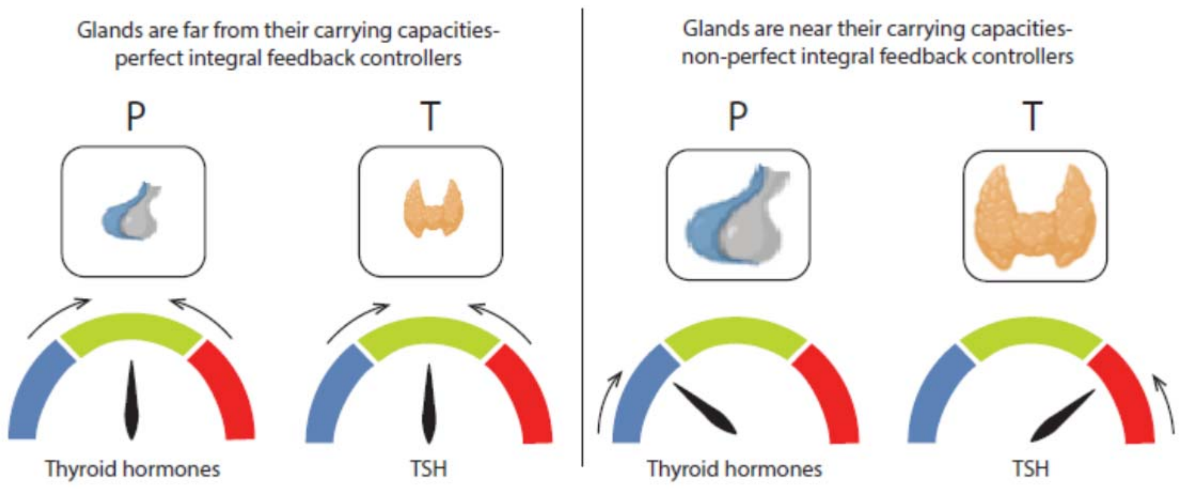

Figure EV1: Thyrotroph mass P and thyrocyte mass T as integral feedback controllers for thyroid hormones and TSH, respectively. Left: When the glands are far from their carrying capacities they provide perfect integral feedback control, keeping the hormones at their homeostatic set points. Right: When the glands approach their carrying capacities they serve as imperfect integral controllers, and can only partially prevent deviation of hormones from their set points.

• It is important to emphasize the use of the proposed model: explain how "locating a patient on the hysteresis curve" can indeed help in the treatment procedure.

We now added to the discussion section on pg 18:

> ***"****For example, estimating thyrotroph mass accumulation rate during treatment in Graves' disease can help to predict the time to recovery and thus help to choose between a conservative to an ablative approach for treatment.****"***

• Suggestion: It would be nice to see a figure where the data and the model results are overlaid. Perhaps some model fitting can be useful here.

This comment helped us compare the new analytical TSH-T4 relation derived in our revisions (see below) to the data of Midgely et al 2013 (DOI 10.1136/jclinpath-2012-201213). The analytical solution with the parameters for the thyroid axis taken form the literature provides a satisfying agreement with the data. This is shown in a new panel in figure 4 (Fig 4D):

D

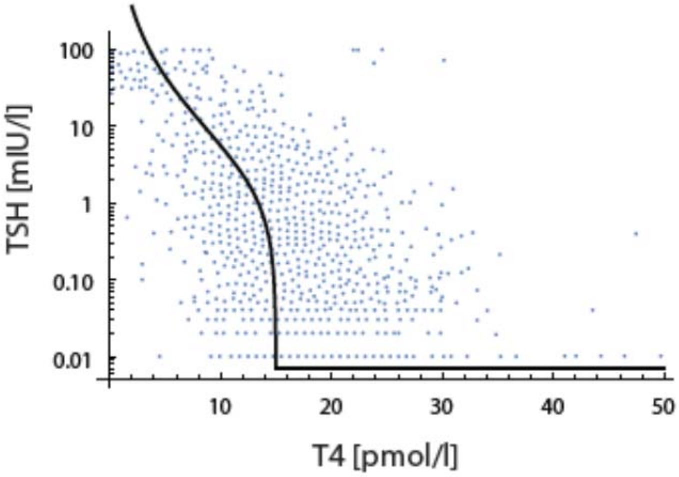

We supply details about the analytical TSH-T4 curve and the data points in the revised Methods section and in a dedicated SI section "The relation between the steady-states of TSH and T4".The Methods section reads as follows:

"Analytical TSH-T4 relation and comparison to data

The TSH-T4 curve in Fig 4C,D was computed from solving the steady-state of the model equations for TRH, TSH and P (eq. 2,3,5) (Appendix Supplementary Text). This gives the following piecewise rational function:

$$\begin{cases} TSH(T4) = \dfrac{b_{TRH}\, b_{TSH}\, u}{a_{TRH}\, a_{TSH}\, k_P} \dfrac{(1 - \frac{a_P}{b_P} T4)}{T4^2} & T4 \leq \dfrac{b_P}{a_P} \\ TSH(T4) \ = \ 0 & T4 > \dfrac{b_P}{a_P} \end{cases}$$

This relation can also be formulated in terms of the steady-state values of T4, TSH and P in the case without carrying capacities, $T4_0, TSH_0, P_0$ (Appendix Supplementary Text):

$$\begin{cases} TSH(T4) = \alpha \dfrac{(1 - \beta\, T4)}{T4^2} & T4 \leq \dfrac{1}{\beta} \\ TSH(T4) \ = \ 0 & T4 > \dfrac{1}{\beta} \end{cases}$$

Where $\alpha = \frac{TSH_0\, T4_0^2}{P_0\, k_P}, \beta = \frac{1}{T4_0}$ *and* $TSH_0 = \frac{a_T}{b_T}, T4_0 = \frac{b_P}{a_P}, P_0 = \frac{a_{TRH}\, a_{TSH}\, a_T\, b_P{}^2}{a_P{}^2\ b_{TRH}\, b_{TSH}\, b_T\, u}$.
To compare the analytical solution to data, we calculated α, β using *typical parameters from the literature. The healthy set-point of the hormones is* $TSH_0 = 1.5 mIU/L$, $T4_0 = 15 pmol/L$. *We use a thyrotroph carrying capacity of 5 times the healthy thyrotroph mass* $P_0 = 5\ Kp$, *following pituitary measurements from Khawaja et al. that showed reduction*

of up to 80% in pituitary volume in hypothyroid patients following treatment (Khawaja, 2006).
Data in Fig 4D was graphically extracted from Midgley et al. (Midgley et al., 2013) using WebPlotDigitizer software. The density of the data points was adjusted for visual clarity.

• Overall, the paper requires some rewriting whenever mathematical arguments are brought up. The detailed derivations should be shown, if not in the main text or methods section, perhaps as supplementary material. It is unfortunate that the authors didn't use a latex-based environment for equations which made them cumbersome to read. The work flow for example can be rewritten as: (1) Write ODEs, (2) show adaptation in ideal cases (KT = KP = infinity), (3) clearly present the assumptions used to make the math easier, (4) divide the fast and slow dynamics and apply quasi-steady state approximation (of course one should prove that the slow manifold is stable to be rigorous), (4) write down the reduced model, (5) write down the nullclines in two dimensions showing the perturbed parameters (aT and Ab), (6) state the new assumptions, compute fixed points, prove stability to explicitly show the bifurcation diagram. It is easy to do all of these rigorously and write them down, and not resort to hand-wavy arguments that leave room for ambiguity and vagueness.

We thank the reviewer for this useful comment that improved the manuscript significantly. We have now addressed all of these points, making the paper more rigorous and complete. In particular, we added a supplementary information text with a detailed derivation and analysis of the model (see below).
We also added and revised Methods sections so that they now contain more details and are more rigorous and clear.
The new Appendix Supplementary Text reads as follows:

Appendix Supplementary Text: Mathematical Model for gland-mass dynamics in the thyroid axis

Model definition

To Model HPT dynamics, we use the following equations:

$$(7)\ \frac{dx_1(t)}{dt} = \frac{b_1\, u}{x_3(t)} - a_1\, x_1(t)$$

$$(8)\ \frac{dx_2(t)}{dt} = \frac{b_2\, P(t) x_1(t)}{x_3(t)} - a_2\, x_2(t)$$

$$(9)\ \frac{dx_3(t)}{dt} = b_{30} + b_3\, T(t) \frac{\left(Ab + x_2(t)\right)}{1 + k_{x2}\left(Ab + x_2(t)\right)} - a_3\, x_3(t)$$

(10) $\frac{dT(t)}{dt} = T(t)(b_T \frac{(Ab+x_2(t))}{1+k_{x2}(Ab+x_2(t))}(1 - k_T\, T(t)) - a_T)$

(11) $\frac{dP(t)}{dt} = P(t)(\frac{b_P}{x_3(t)}(1 - k_P\, P(t)) - a_P)$

Where the variables are:

Table S1: Model variables

x_1	Thyrotropin-releasing hormone (TRH) concentration
x_2	Thyroid-stimulating hormone (TSH) concentration
x_3	Thyroid hormone (TH) concentration, T4 and T3
T	Thyrocyte mass
P	Pituitary thyrotroph mass

We summarize the model parameters in the following table:

Table S2: Model parameters

u	Environmental input
a_1, a_2, a_3	TRH, TSH, TH removal rate respectively
a_T, a_P	Thyroid/pituitary cell removal rate, respectively
b_1, b_2, b_3	TRH, TSH, TH production rate, respectively
b_T, b_P	Thyroid thyrocyte/ Pituitary thyrotroph mass growth rate, respectively
k_T , k_P	Carrying capacity terms for the thyroid/pituitary gland respectively. Note that when $k_T = 0, k_P = 0$, this mean that the glands do not have carrying capacities and can grow indefinitely. Otherwise, T,P maximal masses are $1/k_T$, $1/k_P$ respectively.
Ab	TSH-receptor stimulating antibodies
b_{30}	External thyroid hormone supply.
k_{x2}	Michaelis-Menten coefficient for the response function of TH

Equation (1) describes TRH (x_1) dynamics. TRH production is stimulated by hypothalamic input *u* which represents the integrated effect of cues including temperature, illness and nutritional states, and is inhibited by thyroid hormones T3 and T4 (x_3).
Equation (2) describes TSH (x_2), which is secreted by pituitary thyrotrophs *P*, stimulated by TRH (x_1) and inhibited by TH (x_3).
Equation (3) describes thyroid hormone dynamics (x_3), which is secreted by the thyroid gland, whose functional mass is T, when stimulated by TSH (x_2).
b_{30} represents external thyroid hormone supply. We use this term when simulating levothyroxine treatment of Hashimoto's thyroiditis. Under normal conditions $b_{30} = 0$. Ab represents TSH-receptor stimulating antibodies. We use this term when modeling Grave's disease. Under normal conditions Ab = 0. k_{x2} is a Michaelis-Menten coefficient for the response function of TH. We use this parameter to explore the effect of using a MM response

function for TH, which is more realistic than a linear function. We found that this choice does not affect the qualitative results of the model.

x_1, x_2, x_3 are removed at rates a_1, a_2, a_3 respectively.

Equation (4) for the thyroid functional mass has a removal/turnover term $-a_T\, T$ and a mass growth term activated by TSH (x2). $(1 - k_T\, T)$ is a carrying capacity term, that allows positive growth rate term only for $T < 1/k_T$. The thyroid growth rate is also affected by activating antibodies Ab if they exist, and is generally described by a MM function with MM coefficient k_{x2}.

Equation (5) describes the dynamics of pituitary thyrotroph mass *P*, which follows a similar balance of growth and removal, where we assume that the main control of mass growth is a suppressive effect by TH (x_3). a_P is the removal rate of thyrotrophs, and the carrying capacity (maximal mass) of thyrotrophs is $1/k_P$.

Under normal conditions, both glands are far from their carrying capacities, $kT \cong\ kP \cong 0$. When modeling Hashimoto's thyroiditis, the relevant carrying capacity is that of the pituitary gland (because the thyroid gland is destructed and is thus small), therefore we approximate $kT \cong 0$. When modeling Graves' disease, the relevant carrying capacity is that of the thyroid gland (the pituitary is small due to the negative TH feedback), therefore we approximate $kP \cong 0$.

Steady state of the simple model

In general, the steady-state of the model cannot be computed explicitly. However, we can compute it for a simple model that represents the healthy state, in which there are no TSHR antibodies and no external thyroid hormone supply ($Ab = 0, b_{30} = 0$), the glands are far from their carrying capacities (i.e. infinite carrying capacity $kT =\ kP = 0$), and x_3 depends approximately linearly on x_2 ($k_{x2} = 0$). In this case the steady-state is:

$$\{x_1\ = \frac{a_P\ b_1\ u}{a_1\ b_P}\ , x_2 = \frac{a_T}{b_T}\ , x_3 = \frac{b_P}{a_P}\ , P = \frac{a_1\ a_2\ a_T\ {b_P}^2}{a_P^2\ b_1\ b_2\ b_T\ u}\ \ , T = \frac{a_3\ b_P\ b_T}{a_P\ a_T\ b_3}\}$$

Model rescaling

For the sake of clarity, we begin by a scaling the variables by their steady-state values from the simple model (i.e. $Ab = k_{x2} = b_{30} = k_T = k_P = 0$):

$$\{x_1 \to \frac{a_P\ b_1\ u}{b_P\ a_1}\ , x_2 \to \frac{a_T}{b_T} x_2, x_3 \to \frac{b_P}{a_P} x_3, P \to \frac{b_P^2\ a_1\ a_2\ a_T}{{a_P}^2\ b_1\ b_2\ b_T\ u} P, T \to \frac{a_3\ b_P\ b_T}{b_3\ a_P\ a_T} T\}$$

In addition, we redefine the parameters:

$$Ab = \frac{a_T}{b_T} AB, \qquad k_{x2} = \frac{b_T}{a_T} K_{X2}, \qquad k_T = \frac{a_P\ a_T\ b_3}{b_P\ b_T\ a_3} K_T,$$

$$k_P = \frac{{a_P}^2\ b_1\ b_2\ b_T}{{b_P}^2 a_1\ a_2\ a_T} K_P, \qquad b_{30} = \frac{a_3\ b_P}{a_P} B_{30}$$

Therefore, the model equations transform to the dimensionless form:

(12) $$\frac{1}{a_1} x_1'(t) = \frac{1}{x_3(t)} - x_1(t)$$

(13) $$\frac{1}{a_2} x_2'(t) = P(t) \frac{x_1(t)}{x_3(t)} - x_2(t)$$

(14) $$\frac{1}{a_3} x_3'(t) = B_{30} + \left(T(t) \frac{AB + x_2(t)}{1 + K_{X2}(AB + x_2(t))} - x_3(t)\right)$$

(15) $$\frac{1}{a_T} T'(t) = T(t) \left(\frac{AB + x_2(t)}{1 + K_{X2}(AB + x_2(t))} (1 - K_T\, T(t)) - 1\right)$$

(16) $$\frac{1}{a_P} P'(t) = P(t) \left(\frac{1}{x_3(t)} \left(1 - K_P\, P(t)\right) - 1\right)$$

Note that with the choice $AB = K_{X2} = B_{30} = K_P = K_T = 0$ we recover the simple model.

The model shows exact adaptation only when the glands are far from their carrying capacities

Note that in this simple model, there is exact adaptation for x2 and for x3: the values of these variables depend only on the production and removal rates of the glands. This happens since the glands P and T function as integral feedback controllers, as can be seen in the equations for the simple model below:

$$\frac{dT(t)}{dt} = T(t)(x_2 - 1)$$

$$\frac{dP(t)}{dt} = P(t)\left(\frac{1}{x_3(t)} - 1\right)$$

The gland masses change so that they compensate for any change in the model parameters, except for the production and removal rates of the gland, guaranteeing that $x_2 = x_3 = 1$. Note that x_2 and x_3 were rescaled, so that in terms of the original variables $x_2 = \frac{a_T}{b_T}$ and $x_3 = \frac{b_P}{a_P}$.
Therefore, this simple model cannot account for cases of hypo- or hyperthyroidism.
Adding carrying capacity terms break this perfect adaptation, since the glands can no longer fully compensate for change in the hormone secretion or removal parameters (Fig EV1). In this case, the glands function as non-ideal integral controllers, where the non-ideal aspect comes naturally from the finite carrying capacity.
Adding a carrying capacity term for the pituitary gland breaks the adaptation of x2 (TSH). This can be seen by solving the model steady-state with carrying capacity only for the pituitary, $Ab = AB = K_{X2} = B_{30} = K_P = 0\,, KT \neq 0$:

$$\{x_2 = 1 + K_T, x_3 = 1\}$$

Note that K_T depends on many of the model parameters- $K_T = \frac{a_3\, b_P b_T}{b_3 a_P a_T} K_T$, so that x2 depends now not only on thyrotroph production and removal rates, but also on TH production and removal rates, thyrocyte production and removal rates, and the carrying capacity of the thyroid gland.
Adding a carrying capacity term for the thyroid gland breaks the adaptation of x3 (TH). This can be seen by solving the model steady-state with carrying capacity only for the thyroid, $Ab = B30 = KX2 = AB = KT = 0, KP \neq 0$:

$$\{x_2 = 1, x_3 = \frac{-1 + \sqrt{1 + 4K_P}}{2K_P}\}$$

Again, KP depends on the model parameters, $K_P = \frac{a_1\, a_2\, a_T\, {b_P}^2}{b_1\, b_2\, b_T\, a_P^2} k_P$, making x3 sensitive to change in these parameters.

Computation of nullclines

To compute the nullclines $dT/dt = 0, dP/dt = 0$ we use separation of time scales, using the fact that hormone turnover times are much faster than the gland turnover times. We separate the model to the hormone "fast" equations (6)-(8), and the gland "slow" equations (9)-(10).
We first solve the steady-states for the hormone equations. The solution as a function of P and T can be represented as the roots of a third degree polynomial. For the sake of clarity, we express x1 and x3 using x2, and write an implicit equation for x2:

$$x_1 = \frac{\sqrt{x_2}}{\sqrt{P}}$$
$$x_3 = \frac{\sqrt{P}}{\sqrt{x_2}}$$
$$B_{30} + \left(T\frac{AB + x_2}{1 + K_{X2}(AB + x_2)} - \sqrt{\frac{P}{x_2}}\right) = 0$$

With this, the "slow time scale" system is composed of two ODE's for P and T and one implicit algebraic equation for x2:

(17) $$\frac{1}{a_T}T'(t) = T(t)\left(\frac{AB+x_2(t)}{1+K_{X2}(AB+x_2(t))}(1 - K_T\,T(t)) - 1\right)$$

(18) $$\frac{1}{a_P}P'(t) = P(t)\left(\sqrt{\frac{x_2(t)}{P(t)}}(1 - K_P\,P(t)) - 1\right)$$

(19) $$B_{30} + \left(T\frac{AB+x_2}{1+K_{X2}(AB+x_2)} - \sqrt{\frac{P}{x_2}}\right) = 0$$

In order to find the nullclines we need to solve the equations dT/dt=0 and dP/dt=0. One trivial solution is T=0, P=0.
In order to find the non-trivial solution we solve the equations T'=0 and P'=0 for x2, and substitute the solution into the implicit equation for x2. This gives the following nullclines:
T'=0:

(20) $$\left\{P = \frac{(T+B_{30}(1-K_T\,T))^2}{(1-K_T\,T)^2}\left(\frac{1}{1-K_T\,T-K_{X2}} - AB\right) \quad OR \quad T = 0\right\}$$

P'=0:

(21) $$\left\{T = (1 - B_{30} - K_P P)\frac{\left(1+AB\,K_{X2}\left(1+\frac{P}{(1-K_P P)^2}\right)\right)}{AB+\frac{P}{(1-K_P P)^2}} \quad OR \quad P = 0\right\}$$

The nullclines in the simple case (Fig. EV3A, brought here for convenience), i.e. $B_{30} = K_{X2} = AB = 0$, takes the simple form of:

$$\left\{\ \boldsymbol{P = \frac{T^2}{(1 - K_T\,T)^3} \quad OR \quad T = 0}, \qquad \boldsymbol{T = \frac{(1 - K_P\,P)^3}{P} \quad OR \quad P = 0}\right\}$$

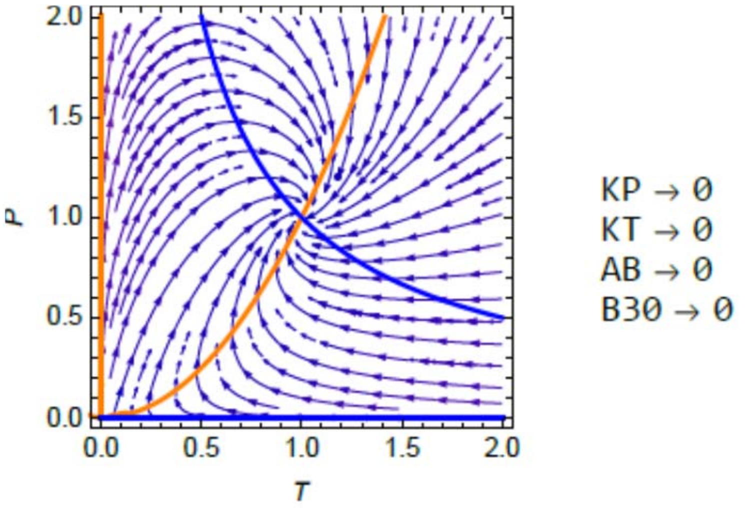

Nullclines and stream plot for the simple model KP=KT=AB=B30=KX2=0

Stability analysis

In order to validate the stability of the slow system we need to find the signs of the eigenvalues of the Jacobian of the reduced system (P,T) (equations (11)-(13)) at the steady state.
The system has four fixed points: (i) one at T>0, P>0, (ii) one at T>0, P=0, and (iii) one at T=0, P=0, and (iv) one at T=0, P>0.
We illustrate the different stability regimes in Figure EV3, which also appears at the end of this section for convenience.

(i) **We first inspect the solution at T>0, P>0** (Fig EV3B,C). To calculate the stability of this fixed point we need to calculate the partial derivative of x2 with respect to P and T.

$$x_2^{(1,0)}(P,T) = \frac{1}{\frac{P}{x_2} + \frac{2\sqrt{P}\,T\sqrt{x_2}}{(1+K_{X2}(AB+x_2))^2}}$$

$$x_2^{(0,1)}(P,T) = -\frac{2x_2^{3/2}(AB+x_2)(1+K_{X2}(AB+x_2))}{2T\,x_2{}^{3/2} + \sqrt{P}(1+K_{X2}(AB+x_2))^2}$$

Calculating the Jacobian and substituting the above, we find that the Jacobian is:

$$J = \begin{pmatrix} at\left(-1+\frac{(1-KT\,T)(AB+x2)}{1+KX2(AB+x2)}-\frac{T(AB+x2)(2x2^{3/2}+KT\sqrt{P}(1+KX2(AB+x2))^2)}{(1+KX2(AB+x2))(2T\,x2^{3/2}+\sqrt{P}(1+KX2(AB+x2))^2)}\right) & -\frac{at\,T(-1+KT\,T)x2}{2\sqrt{P}\,T\,x2^{3/2}+P(1+KX2(AB+x2))^2} \\ \frac{ap\sqrt{P}(-1+KP\,P)x2(AB+x2)(1+KX2(AB+x2))}{2Tx2^{3/2}+\sqrt{P}(1+KX2(AB+x2))^2} & ap\left(-1-KP\sqrt{P}\sqrt{x2}+\frac{(1-KP\,P)\sqrt{x2}}{\sqrt{P}}+\frac{(-1+KP\,P)Tx2^2}{2\sqrt{P}\,T\,x2^{3/2}+P(1+KX2(AB+x2))^2}\right) \end{pmatrix}$$

It is difficult to find the steady-state solution of P,T and x2. However, from (11),(12) we can find a simple expression that connects the steady-state solution of P, T and x2 in this system:

$$\{\frac{(1-K_T\,T)(AB+x_2)}{1+K_{X2}(AB+x_2)} = 1, \qquad (1-K_P\,P)\sqrt{\frac{x_2}{P}} = 1\}$$

Note that from this we learn that a non-negative solution exists only for (1-KT T)>0 and (1-KP P)>0. Substituting this into the Jacobian *J* we find:

$$J = \begin{pmatrix} -\frac{at\,T(AB+x2)(2x2^{3/2}+KT\sqrt{P}(1+KX2(AB+x2))^2)}{(1+KX2(AB+x2))(2T\,x2^{3/2}+\sqrt{P}(1+KX2(AB+x2))^2)} & -\frac{at\,T(-1+KT\,T)x2}{2\sqrt{P}\,T\,x2^{3/2}+P(1+KX2(AB+x2))^2} \\ \frac{ap\sqrt{P}(-1+KP\,P)x2(AB+x2)(1+KX2(AB+x2))}{2Tx2^{3/2}+\sqrt{P}(1+KX2(AB+x2))^2} & ap\left(-KP\sqrt{P}\sqrt{x2}+\frac{(-1+KP\,P)Tx2^2}{2\sqrt{P}\,T\,x2^{3/2}+P(1+KX2(AB+x2))^2}\right) \end{pmatrix}$$

For this steady state solution to be stable, the eigenvalues must all have negative real part. A necessary and sufficient condition for this in 2D systems is that the trace is negative and the determinant is positive. Inspection of *J* shows that this is indeed the case.
Therefore, when this fixed point exists it is stable.

(ii) **We next inspect the stability of the second fixed point at P=0, T>0** (Fig EV3D,E)**.** This fixed point can be computed explicitly:

$$\{x_1 = \frac{K_T(1+AB\,K_{X2})}{-1+AB+B_{30}\,K_T-AB\,K_{X2}+AB\,B_{30}\,K_T\,K_{X2}}, \qquad x_2 = 0,$$
$$x_3 = \frac{-1+AB+B_{30}\,K_T-AB\,K_{X2}+AB\,B_{30}\,K_T\,K_{X2}}{K_T(1+AB\,K_{X2})}, \qquad P = 0,$$
$$T = \frac{-1+AB-AB\,K_{X2}}{AB\,K_T}\}$$

This fixed point is positive only if $AB > \frac{1}{1-K_{X2}}$. If KX2=0 this condition is reduced to **AB>1**.

The eigenvalues of the Jacobian at this fixed point are:

$$\{-1,-1,-1, at + AB\ at(-1+2KX2), \frac{a_P(1+K_T-B_{30}K_T+AB(-1+(1+K_T-B_{30}K_T)K_{X2}))}{-1+AB+B_{30}K_T+AB(-1+B_{30}K_T)K_{X2}}\}$$

The eigenvalues are negative provided that:

$$AB > \frac{1+K_T-B_{30}\,K_T}{1-K_{X2}-K_T\,K_{X2}+B_{30}\,K_T\,K_{X2}}$$

In the simple case when there is no external thyroid hormone supply B30=0, and KX2=0, this condition is reduced to **AB>1+KT**.

(iii) **Third, we inspect the fixed point at (P=0, T=0)** (Fig EV3F)**.** This fixed point can be explicitly computed:

$$\{x_1 = \frac{1}{B_{30}}, x_2 = 0, x_3 = B_{30}, P = 0, T = 0\}$$

Note that this fixed point exists only if there is an external thyroid hormone supply B30>0. The eigenvalues in this case are:

$$\{a_P(-1+\frac{1}{B_{30}}), -1, -1, -1, (-1+AB)a_T\}$$

The eigenvalues are negative providing that **AB<1 and B30>1**.

(iv) Finally, we inspect the stability of the fourth fixed point at **T=0, P>0** (Fig EV3G). This point can be computed explicitly as:

$$\{x_1 = \frac{1}{B_{30}}, x_2 = \frac{1-B_{30}}{B_{30}^2 K_P}, x_3 = B_{30}, P = \frac{1-B_{30}}{K_P}, T = 0\}$$

Note that this fixed point exists only if there is an external thyroid hormone supply B30>0 and that the pituitary gland has a finite carrying capacity $K_P > 0$.

In this case, the eigenvalues of the Jacobian are:

$$\{\frac{a_P(-1+B_{30})}{B_{30}}, -1, -1, -1, a_T(-1+AB+\frac{1-B_{30}}{B_{30}^2\,K_P})\}$$

They are negative providing that **B30<1 and** $\boldsymbol{AB < 1 - \frac{1-B_{30}}{B_{30}^2 K_P}}$

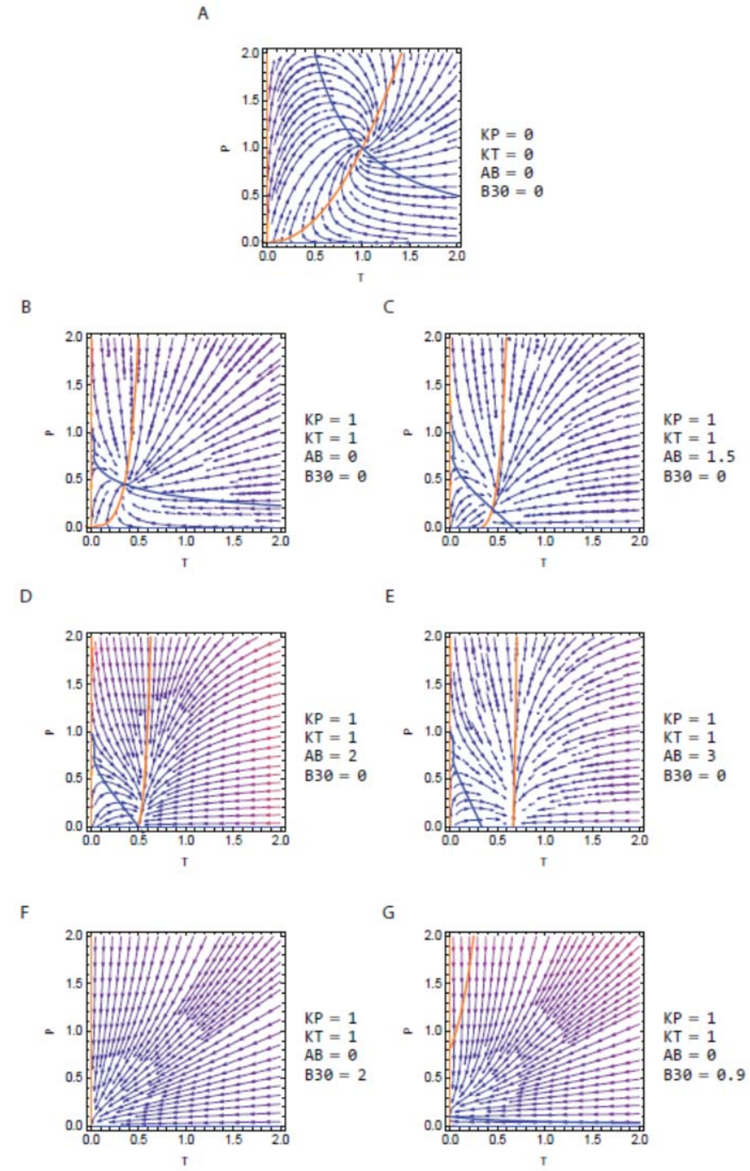

Figure EV3: Nullclines and stream plots for the gland mass model in different parameter regimes. The scaled parameters shown here are $K_T = \frac{a_{TH} b_P b_T}{b_{TH} a_P a_T} k_T$, $K_P = \frac{a_{TRH}\, a_{TSH}\, a_T\, b_P^2}{b_{TRH}\, b_{TSH}\, b_T\, a_P^2} k_P$, $AB = \frac{b_T}{a_T} Ab$, $B_{30} = \frac{a_P}{b_P a_{TH}} b_{30}$. For all graphs $K_{x2} = 0$. For details about the fixed points in the different regimes see Appendix Supplementary Text.

Dependence on antibody parameter in Graves' disease

In this section we analytically explore the model dynamics under perturbation of the Ab parameter in Graves' diseases. We use the results of the stability analysis done above. In

Graves' disease, the pituitary gland is far from its carrying capacity $P \ll \frac{1}{k_P}$, and there is no external thyroid hormone supply $b_{30} = 0$. We also assume for simplicity that $kx2 = 0$. Therefore, the system has two fixed points in the P-T plane (Fig EV4A,B, Fig 4B):

i. $\left\{x_1 = 1, x_2 = 1 - AB + K_T, x_3 = 1, P = 1 - AB + K_T, T = \frac{1}{1+K_T}\right\}$

ii. $\left\{x_1 = \frac{K_T}{AB-1}, x2 = 0, x3 = \frac{AB-1}{K_T}, P = 0, T = \frac{1}{K_T}(1 - \frac{1}{AB})\right\}$

Fig EV4 also appears at the end of this section for convenience.
The black lines in Fig EV4A,B represent fixed point (i), blue lines represent fixed point (ii).
The parameter AB determines the sign and the stability of these two fixed points. When $AB < 1$, we get that only fixed point (i) is positive and stable (black full line in Fig EV4A,B). Above this value, when $1 < AB < 1 + KT$, another unstable positive fixed point arises - fixed point (ii) (blue dashed line in Fig EV4A,B). Up to this point, the thyroid gland T has a constant value: $T = \frac{1}{1+KT}$, which does not depend on AB. The value of thyroid hormones TH (x3) does not depend on AB as well (Fig EV4C). This is possible since the values of P and TSH (x2) do depend on AB - when AB is increased, P shrinks and TSH levels drop (Fig EV4D), compensating for the stimulatory effect of AB. However, when $AB > 1 + KT$, fixed point (i) becomes negative and unstable (black dashed line in Fig EV4A,B) while fixed point (ii) becomes stable (blue full line in Fig EV4A,B), a situation called a transcritical bifurcation. At this point, the pituitary gland has shrunk to zero, TSH=0, and the stimulatory effect of autoantibodies Ab cannot be compensated anymore. From this point on, thyroid gland size T and thyroid hormone level TH rise gradually with Ab: $T = \frac{1}{K_T}(1 - \frac{1}{AB})$, $x_3 = \frac{AB-1}{K_T}$. These dynamics explain the occurrence of subclinical hyperthyroidism, in which TSH levels are low though thyroid hormone levels are kept normal (Fig 4B).

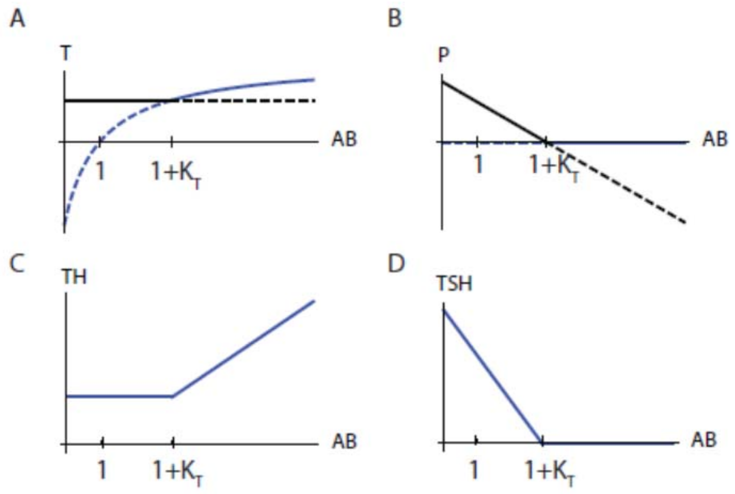

Figure EV4: Graves' disease dynamics under thyroid autoantibodies perturbation. Steady-state values for thyroid gland size T (A), pituitary gland size P (B), thyroid hormone levels TH (C), and TSH levels (D), when perturbing the levels of the normalized thyroid autoantibodies in the system $AB = \frac{b_T}{a_T} Ab$. Black and blue lines in panels A,B are the two fixed points of the system ((1) and (2) respectively, see Appendix Supplementary Text). When $AB < 1 + K_T$ the black fixed-point is stable (full line) while the blue fixed-point is unstable (dashed line). Above this value the fixed point stability switches, in a transcritical bifurcation. When increasing AB up to $1 + K_T$, P shrinks and TSH levels drop, compensating for the autoantibodies stimulatory effect, and allowing for T and TH to remain constant (subclinical hyperthyroidism, Fig 4B, Fig EV3C). Crossing this threshold such that $AB > 1 + K_T$, P and TSH become zero and cannot compensate anymore, and thus T and TH rise together with AB (clinical hyperthyroidism, Fig 4B, Fig EV3E). K_T is the scaled thyroid gland carrying capacity term $K_T = \frac{a_{TH} b_P b_T}{b_{TH} a_P a_T} k_T$.

The relation between the steady-states of TSH and T4

In this section we compute the relation between TSH and T4, here denoted x2 and x3, that appears in Figure 4. Note that in this model, we ignore the complexity of T4 and T3 relation, and treat them as one variable x3.
From the steady-state of the equations for x1, x2 and P (1),(2),(5) we get:

$$x_1 = \frac{b_1 u}{a_1 x_3}$$

$$\begin{cases} P = \dfrac{b_P - a_P\, x_3}{b_P\, k_P} & x_3 < \dfrac{b_P}{a_P} \\ P = 0 & x_3 > \dfrac{b_P}{a_P} \end{cases}$$

Substituting x1 and P in x2 we get the relation x2(x3):

$$\begin{cases} x_2(x_3) = \dfrac{b_1\ b_2\ u}{a_1\ a_2\ k_P}\dfrac{(1-\frac{a_P}{b_P}x_3)}{x_3^2} & x_3 \le \dfrac{b_P}{a_P} \\ x_2(x_3)\ =\ 0 & x_3 > \dfrac{b_P}{a_P} \end{cases}$$

Note that the relation between x2 and x3 can also be written in terms of the steady-state value of these variables and the variable P in the simple model without carrying capacities, (see Appendix section “Steady state of the simple model”):

In terms of these values, we can write:

$$\begin{cases} x_2(x_3) = \alpha\dfrac{(1-\beta\ x_3)}{x_3^2} & x_3 \le \dfrac{1}{\beta} \\ x_2(x_3)\ =\ 0 & x_3 > \dfrac{1}{\beta} \end{cases}$$

Where $\alpha\ = \frac{x2_0\ x3_0{}^2}{P_0\ k_P}, \beta\ = \frac{1}{x30}$, and $x2_0 = \frac{a_T}{b_T}, x3_0 = \frac{b_P}{a_P}, P_0 = \frac{b_P{}^2 a_1\ a_2\ a_T}{a_P{}^2\ b_1\ b_2\ b_T u}$.

Note that $x2_0, x3_0$ are not the steady-state for the full system with carrying capacities. Assuming that in the normal healthy set-point the glands are far from their carrying capacities, we can thus compute α, β. We consider a normal set-point of FT4 $x3_0 = 15pmol/L$, and of TSH $x2_0 = 1.5mIU/L$, and we normalize the pituitary mass units so that in the normal set point $K_P = \frac{P_0}{5}$ following measurements from Khawaja et al. that showed reduction of up to 80% in pituitary volume in hypothyroid patients following treatment. We thus derived a non-parameterized curve for x2(x3). Comparing this curve with data from Midgley et al (Midgley et al., 2013) yield a reasonable agreement (Fig 4D).

Minor Points

• Why aren't the data falling in the normal ranges not shown in Figure 1(G)? Also in the main text, no comment is given on that range and how it compares conceptually to Figure 1(D). The data in Figure 1(G) give the impression that the variation is steeper within the normal range (gray box). Is this correct?

In Figure 1D we show (T4,TSH) measurements that were taken from patients that were diagnosed with either Hashimoto's thyroiditis or with Graves' disease, prior to their diagnosis. In these measurements, the variation is indeed steeper in the normal range. However, these measurements do not reflect TSH-T4 relation in the healthy population. In the healthy population, the variation should approximately fill the normal reference range rectangle shown in the figure (probably forming a diamond shape as suggested by Dietrich et al 2012 (doi: 10.1155/2012/351864). To address this point, we now revised the figure legend (new text in bold):

> *"(G) Pretreatment means and standard errors for Hashimoto (Blue) and Graves (red) patients (same data as in E,F), together with the normal TSH, FT4 range* ***for reference*** *(gray rectangle), forming the inverse "log-linear" TSH-FT4 relation."*

• The causality of these statements: "The one-dimensionality of the problem provides the relation between TSH and T4. This relation has a steep slope at low T4 and high TSH values (Fig 4C, blue line)." is not clear. It is true that as aT (a single parameter) is varied, the fixed point slides on a one-dimensional manifold given by one of the two nullclines that is independent of aT . What does this have to do with providing the relation between TSH and T4? Also, how was the conclusion drawn regarding the slope at low and high TSH values? Statements like these should be proved.

We thank the reviewer for this comment that helped us to explain better the T4-TSH relation in the model. We now removed the statements about the one-dimensionality of the problem. We derive a new analytical solution for TSH(T4). We write the explicit form in the main text, and supply detailed derivations in the Methods section "Computation of the TSH-T4 relation and comparison to data" and in the Appendix Supplementary Text (see above).

We describe this in following new results section:

> ***"The three-regime TSH-T4 relationship***

Analysis of the model explains why different thyroid disorders, such as Hashimoto's thyroiditis, iodine deficiency, Graves' disease, and toxic nodules, cause changes in TSH and T4 that fall on the same predicted curve. The reason for this is that the formula for the curve does not depend on many of the model parameters, and specifically those affected by these disorders. The TSH-T4 curve is a decreasing rational function that reaches zero at high TH and stays there (see Appendix Supplementary Text for details):

$$\begin{cases} TSH(T4) = \dfrac{b_{TRH}\, b_{TSH}\, u}{a_{TRH}\, a_{TSH}\, k_P} \dfrac{(1 - \frac{a_P}{b_P} T4)}{T4^2} & T4 \le \dfrac{b_P}{a_P} \\ TSH(T4) \; = \; 0 & T4 > \dfrac{b_P}{a_P} \end{cases}$$

This relationship does not depend on the production and removal rates of thyroid hormone b_{TH}, a_{TH}, on thyrocyte mass growth rate and removal rates b_T, a_T, on thyroid gland carrying capacity k_T or on antibody level Ab.

Therefore, the model predicts that the same curve for the relation between TSH and T4 remains valid in the conditions that affect any of these parameters. This includes Hashimoto thyroiditis that reduces a_T; Graves' disease which increases Ab; Changing iodine supply which affects b_{TH}, including iodine deficiency in which b_{TH} is low; Hypersecreting nodules which affect b_{TH} and b_T; etc. In all of these conditions, simultaneous measurements of T4 and TSH should fall on the same curve. These parameters would dictate the location of the individual on the curve.

However, conditions which change the pituitary cellular parameters, such as thyrotroph adenoma which affects b_P and b_{TSH}, result in a different curve, and hence measurements from these conditions should fall outside the TSH(T4) curve. The same goes for change in the hypothalamic signal u, for example following change in temperature, nutritional state or health condition.

The relation between TSH and T4 can also be formulated in terms of the steady-state value of these hormones and P in the simple model without carrying capacities (see Appendix Supplementary Text). Using this formulation, we were able to derive a non-parameterized curve for TSH(T4). To do this, we assumed hormone healthy set point of $FT4 \; = 15 pmol/L$, and $TSH \; = 1.5 mIU/L$, and a carrying capacity for the pituitary of 5 times its healthy mass following pituitary measurements from Khawaja et al (Khawaja, 2006). Comparing this curve with data from Midgley et al (Midgley et al., 2013) yield a reasonable agreement (Fig 4D).

In the middle section of the TSH-T4 relation (Fig 4C), which corresponds to hormones in their normal range, the slow time-scale of the glands may play only a secondary role. Remembering the timescale separation between TSH and TH dynamics (hours vs. days), an inverse relation can be explained if TH levels change following tissue demands (Luongo et al., 2019), and TSH reacts accordingly. In this case, $TSH \propto 1/TH^2$ (Fig 4C, green line). The slope of this relation should be parallel to the intra-individual slope of TSH dynamics following T4 treatment, unlike the slopes in the low and high TH regimes, in line with the findings of (Rothacker et al., 2016). "

• When the authors presented equation (2), they used the approximation TH >> K to get the

inverse function model for inhibition. I don't understand why do that if most of the analysis presented is simulation-based and not analytical. This approximation is also inconsistent with the Michaelis-Menten terms used in the Methods Section "Formula for functional thyrotroph mass". If approximations are not needed, it is better not to invoke them.

Indeed, the approximation TH>>K enabled us to carry out an analytical exploration of the model, that allows qualitative understanding of the system behavior under different perturbations. In the Methods section "Formula for functional thyrotroph mass", however, we include a MM term in order to get to a more accurate expression for P. In this section, using such function does not complicate the analytical solution by much, and it can be important if one would like to use this formula to numerically estimate pituitary thyrotroph mass.

*****

In summary, we thank the reviewers for these helpful and insightful comments. The comments helped us revise the paper, which we believe is now more clear, comprehensive and mathematically rigorous.

17th Jun 2022

Manuscript Number: MSB-2022-10919R
Title: Dynamics of thyroid diseases and thyroid-axis gland masses
Author: Uri Alon
Yael Korem Kohanim
Tomer Milo
Moryia Raz
Omer Karin
Alon Bar
Avi Mayo
Netta Mendelson Cohen
Yoel Toledano

Thank you for sending us your revised manuscript. We have now heard back from the two reviewers who agreed to evaluate your study. As you will see, the reviewers are overall satisfied with the modifications made.

Before we can formally accept your manuscript, we would ask you to address the following issues:

1. Please address the remaining concerns of Referee #1 in writing.

2. Please provide up to five keywords and incorporate them into the main manuscript file.

3. Please add corresponding author information to the author list in the manuscript file.

4. Appendix: Change the nomenclature to "Appendix Figure Sx" and update callouts in the manuscript text accordingly.

5. In the Materials and Methods: include a statement confirming that informed consent was obtained from all subjects and that the experiments conformed to the principles set out in the WMA Declaration of Helsinki and the Department of Health and Human Services Belmont Report

6. Reference list: please remove the DOI links.

7. The Author Contribution is incomplete: Tomer Milo, Moriya Raz, Omer Karin, Alon Bar, Yoel Toledano are missing. Please fix it.

8. I have only slightly modified the synopsis text (see attached). Please let me know if it is fine as is or if you would like to introduce further modifications.

9. Our data editors have seen the manuscript, and they have made some comments and suggestions that need to be addressed (see attached file). Please send back a revised version (in track change mode), as we will need to go through the changes.

Click on the link below to submit your revised paper.

https://msb.msubmit.net/cgi-bin/main.plex

As a matter of course, please make sure that you have correctly followed the instructions for authors as given on the submission website.

Thank you for submitting this paper to Molecular Systems Biology.

Kind regards,
Jingyi

Jingyi Hou
Editor
Molecular Systems Biology

Reviewer #1:

Revised Korem's ms has been drastically improved. However I still have a few comments about the rebuttal letter and revised ms, as follows:
1/ In response to one of my comments (rebuttal letter, page 7): "We agree and unfortunately could not find TSH measurements in mice." A few assays allow to measure mouse TSH. Please see doi: 10.1210/en.2016-1788 and also former mTSH RIA from Samuel Refetoff. These data (Endoc 2017) also illustrate how mTSH increases were delayed versus T4 changes upon induced hypothyroidism (Fig.4). Please correct the ms accordingly
2/ About the change in pituitary size in hypothyroidism (rebuttal letter, page 8, first para), the authors should consider the profound changes in the mass of other pituitary cell types. Please see e.g. the elegant studies carried out by Bauer's lab on Pax8-/- mouse model (doi: 10.1210/en.2003-1227)

Reviewer #2:

All issues have been sufficiently addressed. Acceptance may be considered now.

Dear Editor,

Thank you for your positive consideration of our manuscript.

We have now addressed all the remaining concerns of referee #1 in the revised manuscript. We added references and discussion about TSH measurements in rodents and about the effect of thyroid hormones on other pituitary cell types.

On the editorial level, we accepted all the changes that were proposed in the MS and in the synopsis text.

We detail our point-by-point revisions below.

Sincerely,

Uri Alon

Reviewer #1:

Revised Korem's ms has been drastically improved. However I still have a few comments about the rebuttal letter and revised ms, as follows:
1/ In response to one of my comments (rebuttal letter, page 7): "We agree and unfortunately could not find TSH measurements in mice." A few assays allow to measure mouse TSH. Please see doi: 10.1210/en.2016-1788 and also former mTSH RIA from Samuel Refetoff. These data (Endoc 2017) also illustrate how mTSH increases were delayed versus T4 changes upon induced hypothyroidism (Fig.4). Please correct the ms accordingly

We thank the reviewer for this comment which pointed us to important references. We now write in the MS:

> *"It would be important to measure thyrotroph and thyrocyte masses as well as TSH levels as a function of time after thyroid perturbations in humans and rodents (Nolan et al., 2004;* ***Pohlenz et al., 1999; Turgeon et al., 2017****) in order to test the model predictions, such as delays in thyrotroph mass increase after an initial TSH rise.* ***For example, Turgeon et al show a reduction in T4 and an elevation of TSH in mice under low iodine diet. It would be interesting to measure the delay in TSH normalization after returning to normal iodine diet, and test whether high TSH levels correlate with increased thyrotroph mass."***

2/ About the change in pituitary size in hypothyroidism (rebuttal letter, page 8, first para), the authors should consider the profound changes in the mass of other pituitary cell types. Please see e.g. the elegant studies carried out by Bauer's lab on Pax8-/- mouse model (doi: 10.1210/en.2003-1227)

We thank the reviewer for this comment which helped us to add a reference and elaborate on the effect of thyroid hormones on other pituitary cell types.

“In Hashimoto's thyroiditis, the transition from subclinical to clinical disease occurs in the model when the autoimmune killing rate is so high that thyrotroph mass approaches its carrying capacity, and can no longer compensate by increased TSH secretion for the loss of thyroid function. Such a carrying capacity might be due to the confined anatomical position of the pituitary, together with the need for other pituitary cell types. Some experimental reports show a 5-fold increase in pituitary volume in hypothyroidism (Khawaja, 2006) while others show a smaller increase. The thyrotroph carrying capacity requires further study; it may not result in observable volume growth if carrying capacity is imposed by other cell types. Alternatively, a low level of thyroid hormones may be required for thyrotroph proliferation, ***in addition to their effect on other pituitary cell types (Friedrichsen et al., 2004)****.”*

30th Jun 2022
Manuscript number: MSB-2022-10919RR
Title: Dynamics of thyroid diseases and thyroid-axis gland masses

Dear Dr. Alon,

Thank you again for sending us your revised manuscript. We are now satisfied with the modifications made and I am pleased to inform you that your paper has been accepted for publication.

*** PLEASE NOTE *** As part of the EMBO Publications transparent editorial process initiative (see our Editorial at https://dx.doi.org/10.1038/msb.2010.72), Molecular Systems Biology publishes online a Review Process File with each accepted manuscripts. This file will be published in conjunction with your paper and will include the anonymous referee reports, your point- by-point response and all pertinent correspondence relating to the manuscript. If you do NOT want this File to be published, please inform the editorial office at msb@embo.org within 14 days upon receipt of the present letter.

Should you be planning a Press Release on your article, please get in contact with msb@wiley.com as early as possible, in order to coordinate publication and release dates.

LICENSE AND PAYMENT:
All articles published in Molecular Systems Biology are fully open access: immediately and freely available to read, download and share.

Molecular Systems Biology charges an article processing charge (APC) to cover the publication costs. You, as the corresponding author for this manuscript, should have already received a quote with the article processing fee separately. Please let us know in case this quote has not been received.

Once your article is at Wiley for editorial production you will receive an email from Wiley's Author Services system, which will ask you to log in and will present you with the publication license form for completion. Within the same system the publication fee can be paid by credit card, an invoice or pro forma can be requested.

Payment of the publication charge and the signed Open Access Agreement form must be received before the article can be published online.

Molecular Systems Biology articles are published under the Creative Commons licence CC BY, which facilitates the sharing of scientific information by reducing legal barriers, while mandating attribution of the source in accordance to standard scholarly practice.

Proofs will be forwarded to you within the next 2-3 weeks.

Thank you very much for submitting your work to Molecular Systems Biology.

Sincerely,
Jingyi

Jingyi Hou
Editor
Molecular Systems Biology

EMBO Press Author Checklist

Corresponding Author Name: Uri Alon	
Journal Submitted to: MSB	
Manuscript Number: MSB-2022-10919	

USEFUL LINKS FOR COMPLETING THIS FORM
The EMBO Journal - Author Guidelines
EMBO Reports - Author Guidelines
Molecular Systems Biology - Author Guidelines
EMBO Molecular Medicine - Author Guidelines

Reporting Checklist for Life Science Articles (updated January 2022)
This checklist is adapted from Materials Design Analysis Reporting (MDAR) Checklist for Authors. MDAR establishes a minimum set of requirements in transparent reporting in the life sciences (see Statement of Task: 10.31222/osf.io/9sm4x). Please follow the journal's guidelines in preparing your manuscript.
Please note that a copy of this checklist will be published alongside your article.

Abridged guidelines for figures
1. Data
The data shown in figures should satisfy the following conditions:
- the data were obtained and processed according to the field's best practice and are presented to reflect the results of the experiments in an accurate and unbiased manner.
- ideally, figure panels should include only measurements that are directly comparable to each other and obtained with the same assay.
- plots include clearly labeled error bars for independent experiments and sample sizes. Unless justified, error bars should not be shown for technical replicates.
- if n<5, the individual data points from each experiment should be plotted. Any statistical test employed should be justified.
- Source Data should be included to report the data underlying figures according to the guidelines set out in the authorship guidelines on Data Presentation.

2. Captions
Each figure caption should contain the following information, for each panel where they are relevant:
- a specification of the experimental system investigated (eg cell line, species name).
- the assay(s) and method(s) used to carry out the reported observations and measurements.
- an explicit mention of the biological and chemical entity(ies) that are being measured.
- an explicit mention of the biological and chemical entity(ies) that are altered/varied/perturbed in a controlled manner.
- the exact sample size (n) for each experimental group/condition, given as a number, not a range;
- a description of the sample collection allowing the reader to understand whether the samples represent technical or biological replicates (including how many animals, litters, cultures, etc.).
- a statement of how many times the experiment shown was independently replicated in the laboratory.
- definitions of statistical methods and measures:
  - common tests, such as t-test (please specify whether paired vs. unpaired), simple χ2 tests, Wilcoxon and Mann-Whitney tests, can be unambiguously identified by name only, but more complex techniques should be described in the methods section;
  - are tests one-sided or two-sided?
  - are there adjustments for multiple comparisons?
  - exact statistical test results, e.g., P values = x but not P values < x;
  - definition of 'center values' as median or average;
  - definition of error bars as s.d. or s.e.m.

Please complete ALL of the questions below.
Select "Not Applicable" only when the requested information is not relevant for your study.

Materials

Newly Created Materials	Information included in the manuscript?	In which section is the information available? (Reagents and Tools Table, Materials and Methods, Figures, Data Availability Section)
New materials and reagents need to be available; do any restrictions apply?	Not Applicable	

Antibodies	Information included in the manuscript?	In which section is the information available? (Reagents and Tools Table, Materials and Methods, Figures, Data Availability Section)
For **antibodies** provide the following information:<br>- Commercial antibodies: RRID (if possible) or supplier name, catalogue number and or/clone number<br>- Non-commercial: RRID or citation	Not Applicable	

DNA and RNA sequences	Information included in the manuscript?	In which section is the information available? (Reagents and Tools Table, Materials and Methods, Figures, Data Availability Section)
**Short novel DNA or RNA including primers, probes**: provide the sequences.	Not Applicable	

Cell materials	Information included in the manuscript?	In which section is the information available? (Reagents and Tools Table, Materials and Methods, Figures, Data Availability Section)
**Cell lines:** Provide species information, strain. Provide accession number in repository **OR** supplier name, catalog number, clone number, and/**OR** RRID.	Not Applicable	
**Primary cultures:** Provide species, strain, sex of origin, genetic modification status.	Not Applicable	
Report if the cell lines were recently **authenticated** (e.g., by STR profiling) and tested for mycoplasma contamination.	Not Applicable	

Experimental animals	Information included in the manuscript?	In which section is the information available? (Reagents and Tools Table, Materials and Methods, Figures, Data Availability Section)
**Laboratory animals or Model organisms:** Provide species, strain, sex, age, genetic modification status. Provide accession number in repository **OR** supplier name, catalog number, clone number, **OR** RRID.	Not Applicable	
**Animal observed in or captured from the field:** Provide species, sex, and age where possible.	Not Applicable	
Please detail **housing and husbandry conditions**.	Not Applicable	

Plants and microbes	Information included in the manuscript?	In which section is the information available? (Reagents and Tools Table, Materials and Methods, Figures, Data Availability Section)
**Plants:** provide species and strain, ecotype and cultivar where relevant, unique accession number if available, and source (including location for collected wild specimens).	Not Applicable	
**Microbes:** provide species and strain, unique accession number if available, and source.	Not Applicable	

Human research participants	Information included in the manuscript?	In which section is the information available? (Reagents and Tools Table, Materials and Methods, Figures, Data Availability Section)
If collected and within the bounds of privacy constraints report on age, sex and gender or ethnicity for all study participants.	Yes	Materials and Methods

Core facilities	Information included in the manuscript?	In which section is the information available? (Reagents and Tools Table, Materials and Methods, Figures, Data Availability Section)
If your work benefited from core facilities, was their service mentioned in the acknowledgments section?	Not Applicable	

Design

Study protocol	Information included in the manuscript?	In which section is the information available? (Reagents and Tools Table, Materials and Methods, Figures, Data Availability Section)
If study protocol has been **pre-registered, provide DOI in the manuscript**. For clinical trials, provide the trial registration number **OR** cite DOI.	Not Applicable	
Report the **clinical trial registration number** (at ClinicalTrials.gov or equivalent), where applicable.	Not Applicable	

Laboratory protocol	Information included in the manuscript?	In which section is the information available? (Reagents and Tools Table, Materials and Methods, Figures, Data Availability Section)
Provide DOI OR other citation details if **external detailed step-by-step protocols** are available.	Not Applicable	

Experimental study design and statistics	Information included in the manuscript?	In which section is the information available? (Reagents and Tools Table, Materials and Methods, Figures, Data Availability Section)
Include a statement about **sample size** estimate even if no statistical methods were used.	Yes	Materials and Methods
Were any steps taken to minimize the effects of subjective bias when allocating animals/samples to treatment (e.g. **randomization procedure**)? If yes, have they been described?	Not Applicable	
Include a statement about **blinding** even if no blinding was done.	Not Applicable	
Describe **inclusion/exclusion criteria** if samples or animals were excluded from the analysis. Were the criteria pre-established?<br><br>If sample or data points were omitted from analysis, report if this was due to attrition or intentional exclusion and provide justification.	Select response	Materials and Methods
For every figure, are **statistical tests** justified as appropriate? Do the data meet the assumptions of the tests (e.g., normal distribution)? Describe any methods used to assess it. Is there an estimate of variation within each group of data? Is the variance similar between the groups that are being statistically compared?	Yes	Materials and Methods, Figures

Sample definition and in-laboratory replication	Information included in the manuscript?	In which section is the information available? (Reagents and Tools Table, Materials and Methods, Figures, Data Availability Section)
In the figure legends: state number of times the experiment was **replicated** in laboratory.	Not Applicable	
In the figure legends: define whether data describe **technical or biological replicates**.	Not Applicable	

Ethics

Ethics	Information included in the manuscript?	In which section is the information available? (Reagents and Tools Table, Materials and Methods, Figures, Data Availability Section)
Studies involving **human participants**: State details of **authority granting ethics approval** (IRB or equivalent committee(s), provide reference number for approval.	Yes	Materials and Methods
Studies involving **human participants**: Include a statement confirming that **informed consent** was obtained from all subjects and that the experiments conformed to the principles set out in the WMA Declaration of Helsinki and the Department of Health and Human Services Belmont Report.	Not Applicable	
Studies involving **human participants:** For publication of **patient photos**, include a statement confirming that consent to publish was obtained.	Not Applicable	
Studies involving experimental **animals**: State details of **authority granting ethics approval** (IRB or equivalent committee(s), provide reference number for approval. Include a statement of compliance with ethical regulations.	Not Applicable	
Studies involving **specimen and field samples:** State if relevant **permits** obtained, provide details of authority approving study; if none were required, explain why.	Not Applicable	

Dual Use Research of Concern (DURC)	Information included in the manuscript?	In which section is the information available? (Reagents and Tools Table, Materials and Methods, Figures, Data Availability Section)
Could your study fall under dual use research restrictions? Please check biosecurity documents and list of **select agents and toxins** (CDC): https://www.selectagents.gov/sat/list.htm	Not Applicable	
If you used a select agent, is the security level of the lab appropriate and reported in the manuscript?	Not Applicable	
If a study is subject to dual use research of concern regulations, is the name of the **authority granting approval and reference number** for the regulatory approval provided in the manuscript?	Not Applicable	

Reporting

The MDAR framework recommends adoption of discipline-specific guidelines, established and endorsed through community initiatives. Journals have their own policy about requiring specific guidelines and recommendations to complement MDAR.

Adherence to community standards	Information included in the manuscript?	In which section is the information available? (Reagents and Tools Table, Materials and Methods, Figures, Data Availability Section)
State if relevant guidelines or checklists (e.g., **ICMJE, MIBBI, ARRIVE, PRISMA**) have been followed or provided.	Not Applicable	
For **tumor marker prognostic studies,** we recommend that you follow the **REMARK** reporting guidelines (see link list at top right). See author guidelines, under 'Reporting Guidelines'. Please confirm you have followed these guidelines.	Not Applicable	
For **phase II and III randomized controlled trials**, please refer to the **CONSORT** flow diagram (see link list at top right) and submit the CONSORT checklist (see link list at top right) with your submission. See author guidelines, under 'Reporting Guidelines'. Please confirm you have submitted this list.	Not Applicable	

Data Availability

Data availability	Information included in the manuscript?	In which section is the information available? (Reagents and Tools Table, Materials and Methods, Figures, Data Availability Section)
Have **primary datasets** been deposited according to the journal's guidelines (see 'Data Deposition' section) and the respective accession numbers provided in the Data Availability Section?	Not Applicable	
Were **human clinical and genomic datasets** deposited in a public access-controlled repository in accordance to ethical obligations to the patients and to the applicable consent agreement?	Not Applicable	
Are **computational models** that are central and integral to a study available without restrictions in a machine-readable form? Were the relevant accession numbers or links provided?	Yes	Materials and Methods
If publicly available data were reused, provide the respective **data citations in the reference list**.	Yes	References, Materials and Methods, Figures
